# Synthesis of Thiazole-methylsulfonyl Derivatives, X-ray Study, and Investigation of Their Carbonic Anhydrase Activities: *In Vitro* and *In Silico* Potentials

**DOI:** 10.1021/acsomega.5c00509

**Published:** 2025-03-27

**Authors:** Zahra Maryam, Ayşen Işık, Emine Rana Bağcı, Maksut Yıldız, Hakan Ünver, Ümit M. Kocyigit, Burak Kırılmaz, Ismail Celik, Ulviye Acar Çevik, Yusuf Özkay, Zafer Asım Kaplancıklı

**Affiliations:** 1 Department of Pharmaceutical Chemistry, Faculty of Pharmacy, Anadolu University, Eskişehir 26470, Turkey; 2 Department of Biochemistry, Faculty of Science, Selçuk University, Konya 42250, Turkey; 3 Department of Pharmaceutical Chemistry, Faculty of Pharmacy, Afyonkarahisar Health Sciences University, Afyonkarahisar 03030, Turkey; 4 Department of Biochemistry, Faculty of Pharmacy, Cumhuriyet University, Sivas 58140, Turkey; 5 Department of Chemistry, Faculty of Science, Eskisehir Technical University, Eskişehir 26470, Turkey; 6 Department of Pharmaceutical Chemistry, Faculty of Pharmacy, Erciyes University, Kayseri 38039, Turkey; 7 Department of Pharmaceutical Chemistry, Graduate School, Anadolu University, Eskişehir 26470, Turkey; 8 The Rectorate of Bilecik Şeyh Edebali University, Bilecik 11230, Turkey

## Abstract

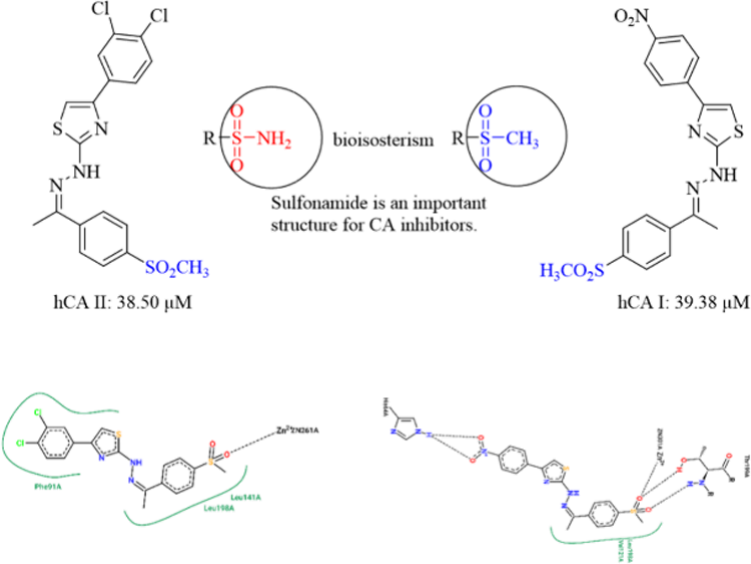

This study focused on the design, synthesis, chemical characterization, and potential inhibitory study of thiazole-methylsulfonyl derivatives against carbonic anhydrase enzymes. The synthesized compounds, with the characteristics of both the thiazole ring and methyl sulfonyl group, were synthesized through a two-step scheme, and their structures were confirmed through NMR spectroscopy and HRMS. Additionally, the structure of compound **2b** was elucidated by an X-ray study. An enzyme inhibition assay was performed to assess their biological activity against carbonic anhydrases, and the compounds showed promising results against carbonic anhydrases I and II, highlighting their potential for specificity and targeted therapy. The effects of these molecules on *in vitro* enzyme activities were investigated by spectrophotometric methods. For this purpose, the concentrations (IC_50_ values) of compounds that inhibited the biological activities of carbonic anhydrase isoenzymes (hCA I and hCA II) by 50% were calculated. The IC_50_ values were found between 39.38–198.04 μM (AAZ IC_50_ = 18.11 μM) for hCA I and 39.16–86.64 μM (AAZ IC_50_ = 20.65 μM). Molecular docking studies have shown that compounds **2a** and **2h** exhibit stable interaction networks with targeted enzymes. The combinations of both studies, enzyme inhibition assay and molecular docking studies, thus enlighten the significance of these compounds for further optimization for pharmacological profiling and for developing therapeutic agents against carbonic anhydrase. Moreover, the study provides insight for future research on the synthesis of heterocyclic compounds against carbonic anhydrase for therapeutic applications.

## Introduction

Carbonic anhydrase (CA) is a metalloenzyme that catalyzes the hydration of CO_2_ to bicarbonate anion (HCO^3–^) and a proton (H^+^).^1−3^ The enzyme usually contains Zn^2+^ ions in its active side.^1−3^ Through these active side inhibitors of CA, isoenzymes are used as many therapeutic targets.^4^ The enzyme is also available in mammals, other animals, and plants. Among mammals and humans, there are 16 different isoforms of α-CA with different locations and metabolization functions in the human body.^5^ Among these isoforms, in particular, hCA I and hCA II play a role in many therapeutic applications, and hCA I is involved in retinal and brain tissues. CA II is implicated in a number of illnesses, including epilepsy, glaucoma, and most likely altitude sickness.^6^ According to studies in the literature, hCA I and hCA II are located at high levels in some human malignant tumors.^7−9^ In addition, high levels of those enzymes cause some diseases like glaucoma and edema.^10−12^ Because of that, the literature focuses on how to inhibit those enzymes.^13^ Additionally, due to the widespread expression of various isoforms of hCAs and cytosolic isoforms hCA I and II in the human body, controlling selectivity toward target isoforms (hCA I and II) is very important.^14^ Because of the competition between different CA isoforms, both reduce the efficacy of inhibitors and lead to unwanted side effects.^15^

Heterocyclic compounds are generally used in chemistry, especially heterocyclic compounds with sulfur and nitrogen atoms, which are essential in targeting enzyme inhibition, such as thiazole and some sulfonyl groups.^16^

**Figure 1:**
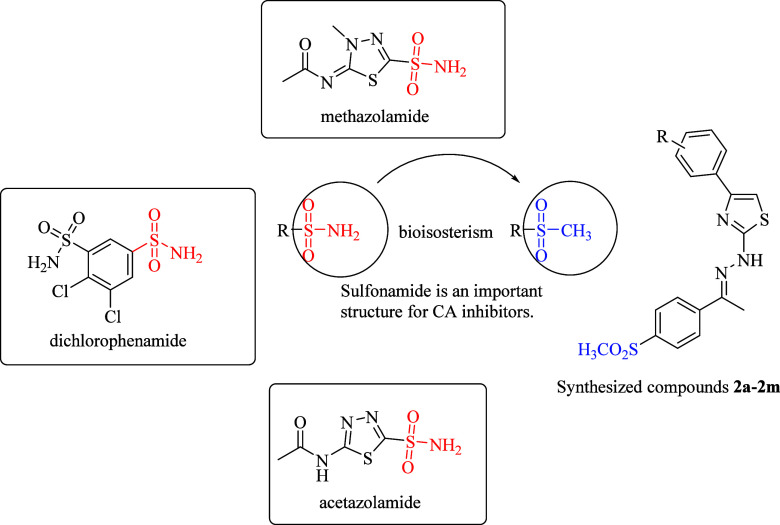
Some carbonic anhydrase inhibitors and synthesized compounds.

Isosterism and bioisosterism are frequently used in active research areas because they can be applied in studies to optimize directly.^17^ When the methyl sulfonyl group is examined in terms of structure, it is thought that it is similar to the structures of sulfonamides and may show similar activity.^18^ When we look at these structures, which contain the sulfo group carrying the sulfur element, we see that they are the most frequently used molecule class due to their wide range of biological activities in the drug development phase.^19−21^ This group of compounds can directly coordinate with the Zn^2+^ ion, one of the metal ions in the active site of the carbonic anhydrase enzyme, representing the most suitable class of carbonic anhydrase inhibitors.^15,22,23^ Some drugs bearing the sulfonamide structure and the general structure of the designed compounds are shown in Figure 1.

Thiazole has a five-membered ring that contains N and S atoms in the chemical structure, and this ring with sulfonyl systems like sulfonamides and their bioisosteres like some sulfonyl alkyl groups are used in pharmaceutical industries due to biological activities.^24−26^ Because thiazole and some sulfonyl groups such as methyl sulfonyl and their bioisosteres as sulfonamides groups have an excellent pharmacophore nucleus, that nucleus is used in various diseases^27^ like anticancer,^28,29^ antioxidant, antiparasitic,^30,31^ antimalarial,^32,33^ antimicrobial,^34,35^ anti-inflammatory,^36,37^ analgesic,^38^ and antianxiety activities^39^ and targeting enzyme inhibition such as cholinesterase^6^ and carbonic anhydrase enzymes, especially CA I and CA II enzyme groups.

In our previous study, compounds containing thiazole structure were synthesized and their effects on the carbonic anhydrase enzyme were examined. Their structures appear to have significant interactions with the active site amino acids leading to inhibition of the enzyme.^4^ It was concluded that both the thiazole ring and the methyl sulfonyl group are important for carbonic anhydrase enzyme inhibition. In this study, compounds containing these two structures that are thought to be effective were designed.

In this study, compounds containing the thiazole group with a methyl sulfonyl moiety scaffold together were synthesized, and their structures were elucidated using ^1^H NMR, ^13^C NMR, and HRMS. The molecular structure of the compound **2b** was confirmed by the single-crystal X-ray diffraction study. The inhibitory effects of the synthesized target compounds on hCAI and hCA II enzymes were tested *in vitro* and *in silico* with molecular docking and molecular dynamics simulations. Additionally, to predict the pharmacokinetic profile of synthesized drugs, an *in silico* ADME study was performed.

## Materials and Methods

### Chemistry

All the chemicals employed in the synthetic procedure were purchased from Sigma-Aldrich Chemicals (Sigma-Aldrich Corp., St. Louis, MO, USA) or Merck Chemicals (Merck KGaA, Darmstadt, Germany). Melting points of the obtained compounds were determined by an MP90 digital melting point apparatus (Mettler Toledo, OH, USA) and were uncorrected. ^1^H NMR and ^13^C NMR spectra of the synthesized compounds were performed by a Bruker 400 and 100 MHz digital FT-NMR spectrometer (Bruker Bioscience, Billerica, MA, USA) in DMSO-*d*_6_, respectively. Splitting patterns were designated as follows: s, singlet; d, doublet; t, triplet; m, multiplet in the NMR spectra. Coupling constants (*J*) were reported as Hertz. All reactions were monitored by thin-layer chromatography (TLC) using silica gel 60 F254 TLC plates (Merck KGaA, Darmstadt, Germany).

#### 2-(1-(4-(Methylsulfonyl)phenyl)ethylidene)hydrazine-1-carbothioamide (**1**)

4′-(Methylsulfonyl) acetophenone (4 g, 0.018 mol) was dissolved in ethanol (50 mL), and thiosemicarbazide (1.67 g, 0.018 mol) was added. The reaction mixture was refluxed for 4–5 h. The completion of the reaction was checked by TLC. The precipitated product was filtered while hot. Yield: 78%.

#### 2-(2-(1-(4-(Methylsulfonyl)phenyl)ethylidene)hydrazinyl)-4-(substitutedphenyl)thiazole (**2a**–**2m**)

The appropriate 2-bromoacetophenone derivative (0.001 mol) and compound (**1**) (0.271 g, 0.001 mol) were dissolved in ethanol (40 mL). The mixture was refluxed for 4 h. After TLC screening, the precipitated product was filtered while hot. Derivatives that did not precipitate when hot were filtered after cooling. The precipitated product was washed three times with cold ethanol (10 mL).

#### 2-(2-(1-(4-(Methylsulfonyl)phenyl)ethylidene)hydrazinyl)-4-(4-nitrophenyl)thiazole (**2a**)

M.p. = 296.1 °C. ^1^H NMR (400 MHz, DMSO-*d*_6_): δ 2.39 (3H, s, CH_3_), 3.25 (3H, s, CH_3_), 7.80 (1H, s, thiazole CH), 7.97 (2H, d, *J* = 7.56 Hz, 1,4-disubstituted benzene), 8.01 (2H, d, *J* = 7.56 Hz, 1,4-disubstituted benzene), 8.15 (2H, d, *J* = 7.64 Hz, 1,4-disubstituted benzene), 8.30 (2H, d, *J* = 7.72 Hz, 1,4-disubstituted benzene), 11.68 (1H, s, NH). ^13^C NMR (100 MHz, DMSO-*d*_6_): δ 14.56 (CH_3_), 44.01 (CH_3_), 109.92, 124.64, 126.83, 126.88, 127.66, 136.09, 140.84, 141.18, 142.97, 146.71, 161.33, 170.36. HRMS (*m*/*z*): [M + H]^+^ calcd for C_18_H_16_N_4_O_4_S_2_, 417.0686; found, 417.0676.

#### 2-(2-(1-(4-(Methylsulfonyl)phenyl)ethylidene)hydrazinyl)-4-(4-methoxyphenyl)thiazole (**2b**)

M.p. = 247.9 °C. ^1^H NMR (400 MHz, DMSO-*d*_6_): δ 2.38 (3H, s, CH_3_), 3.24 (3H, s, CH_3_), 3.79 (3H, s, OCH_3_), 6.98 (2H, d, *J* = 7.96 Hz, 1,4-disubstituted benzene), 7.20 (1H, s, thiazole CH), 7.80 (2H, d, *J* = 8.04 Hz, 1,4-disubstituted benzene), 7.96 (2H, d, *J* = 7.68 Hz, 1,4-disubstituted benzene), 8.01 (2H, d, *J* = 8.00 Hz, 1,4-disubstituted benzene). ^13^C NMR (100 MHz, DMSO-*d*_6_): δ 14.52 (CH_3_), 44.03 (CH_3_), 55.62 (OCH_3_), 102.88, 114.49, 126.84, 127.41, 127.63, 129.30, 129.73, 140.72, 143.06, 145.49, 159.37, 169.82. HRMS (*m*/*z*): [M + H]^+^ calcd for C_19_H_19_N_3_O_3_S_2_, 402.0941; found, 402.0947.

#### 2-(2-(1-(4-(Methylsulfonyl)phenyl)ethylidene)hydrazinyl)-4-(4-cyanophenyl)thiazole (**2c**)

M.p. = 287.2 °C. ^1^H NMR (400 MHz, DMSO-*d*_6_): δ 2.38 (3H, s, CH_3_), 3.25 (3H, s, CH_3_), 7.70 (1H, s, thiazole CH), 7.88 (2H, d, *J* = 7.64 Hz, 1,4-disubstituted benzene), 7.96 (2H, d, *J* = 7.60 Hz, 1,4-disubstituted benzene), 8.00 (2H, d, *J* = 7.68 Hz, 1,4-disubstituted benzene), 8.06 (2H, d, *J* = 7.60 Hz, 1,4-disubstituted benzene), 11.62 (1H, s, NH). ^13^C NMR (100 MHz, DMSO-*d*_6_): δ 14.55 (CH_3_), 44.02 (CH_3_), 108.92, 110.09, 119.44, 126.60, 126.86, 127.65, 133.19, 139.29, 140.79, 143.00, 145.48, 149.56, 170.26. HRMS (*m*/*z*): [M + H]^+^ calcd for C_19_H_16_N_4_O_2_S_2_, 397.0787; found, 397.0776.

#### 2-(2-(1-(4-(Methylsulfonyl)phenyl)ethylidene)hydrazinyl)-4-(4-fluorophenyl)thiazole (**2d**)

M.p. = 253.6 °C. ^1^H NMR (400 MHz, DMSO-*d*_6_): δ 2.38 (3H, s, CH_3_), 3.24 (3H, s, CH_3_), 7.25 (2H, t, *J* = 8.24 Hz, 1,4-disubstituted benzene), 7.37 (1H, s, thiazole CH), 7.95–7.98 (2H, m, 1,4-disubstituted benzene), 8.01 (2H, d, *J* = 7.88 Hz, 1,4-disubstituted benzene), 11.55 (1H, s, NH). ^13^C NMR (100 MHz, DMSO-*d*_6_): δ 14.47 (CH_3_), 44.02 (CH_3_), 104.78, 115.83, 116.05, 126.80, 127.64, 127.94, 128.03, 140.71, 143.10, 160.87, 163.30, 170.03. HRMS (*m*/*z*): [M + H]^+^ calcd for C_18_H_16_N_3_O_2_FS_2_, 390.0741; found, 390.0753.

#### 4-([1,1′-Biphenyl]-4-yl)-2-(2-(1-(4-(methylsulfonyl)phenyl)ethylidene)hydrazinyl)thiazole (**2e**)

M.p. = 217.9 °C. ^1^H NMR (400 MHz, DMSO-*d*_6_): δ 2.39 (3H, s, CH_3_), 3.25 (3H, s, CH_3_), 7.39 (1H, d, *J* = 7.24 Hz, aromatic CH), 7.46–7.50 (3H, m, aromatic CH), 7.74 (4H, d, *J* = 6.92 Hz, aromatic CH), 7.96–8.03 (6H, m, aromatic CH). ^13^C NMR (100 MHz, DMSO-*d*_6_): δ 14.48 (CH_3_), 44.03 (CH_3_), 105.38, 126.59, 126.82, 126.94, 127.25, 127.34, 127.65, 127.87, 127.96, 129.45, 139.56, 140.10, 140.71, 143.12, 170.02. HRMS (*m*/*z*): [M + H]^+^ calcd for C_24_H_21_N_3_O_2_S_2_, 448.1148; found, 448.1159.

#### 2-(2-(1-(4-(Methylsulfonyl)phenyl)ethylidene)hydrazinyl)-4-(4-bromophenyl)thiazole (**2f**)

M.p. = 244.8 °C. ^1^H NMR (400 MHz, DMSO-*d*_6_): δ 2.38 (3H, s, CH_3_), 3.24 (3H, s, CH_3_), 7.47 (1H, s, thiazole CH), 7.61 (2H, d, *J* = 7.80 Hz, 1,4-disubstituted benzene), 7.84 (2H, d, *J* = 7.72 Hz, 1,4-disubstituted benzene), 7.96 (2H, d, *J* = 7.88 Hz, 1,4-disubstituted benzene), 8.01 (2H, d, *J* = 7.76 Hz, 1,4-disubstituted benzene), 11.56 (1H, s, NH). ^13^C NMR (100 MHz, DMSO-*d*_6_): δ 14.49 (CH_3_), 44.02 (CH_3_), 105.91, 121.03, 126.82, 127.65, 128.04, 132.04, 134.34, 140.74, 143.06, 145.28, 149.99, 170.05. HRMS (*m*/*z*): [M + H]^+^ calcd for C_18_H_16_N_3_O_2_S_2_Br, 449.9940; found, 449.9956.

#### 2-(2-(1-(4-(Methylsulfonyl)phenyl)ethylidene)hydrazinyl)-4-(4-methylphenyl)thiazole (**2g**)

M.p. = 270.9 °C. ^1^H NMR (400 MHz, DMSO-*d*_6_): δ 2.33 (3H, s, CH_3_), 2.38 (3H, s, CH_3_), 3.24 (3H, s, CH_3_), 7.23 (2H, t, *J* = 7.44 Hz, 1,4-disubstituted benzene), 7.30 (1H, s, thiazole CH), 7.77 (2H, d, *J* = 7.44 Hz, 1,4-disubstituted benzene), 7.96 (2H, d, *J* = 7.88 Hz, 1,4-disubstituted benzene), 8.01 (2H, t, *J* = 8.04 Hz, 1,4-disubstituted benzene). ^13^C NMR (100 MHz, DMSO-*d*_6_): δ 14.46 (CH_3_), 21.27 (CH_3_), 44.03 (CH_3_), 104.03, 125.98, 126.80, 127.64, 128.26, 129.37, 129.67, 137.35, 140.69, 143.12, 150.56, 169.84. HRMS (*m*/*z*): [M + H]^+^ calcd for C_19_H_19_N_3_O_2_S_2_, 386.0991; found, 386.0997.

#### 2-(2-(1-(4-(Methylsulfonyl)phenyl)ethylidene)hydrazinyl)-4-(3,4-dichlorophenyl)thiazole (**2h**)

M.p. = 261.3 °C. ^1^H NMR (400 MHz, DMSO-*d*_6_): δ 2.38 (3H, s, CH_3_), 3.24 (3H, s, CH_3_), 7.61 (1H, s, thiazole CH), 7.68 (1H, d, *J* = 8.28 Hz, aromatic CH), 7.87 (1H, d, *J* = 8.28 Hz, aromatic CH), 7.96 (2H, d, *J* = 7.88 Hz, 1,4-disubstituted benzene), 8.00 (2H, d, *J* = 8.00 Hz, 1,4-disubstituted benzene), 8.13 (1H, s, aromatic CH), 11.58 (1H, s, NH). ^13^C NMR (100 MHz, DMSO-*d*_6_): δ 14.50 (CH_3_), 44.02 (CH_3_), 107.26, 126.01, 126.84, 127.64, 127.69, 130.17, 131.36, 131.93, 135.71, 140.77, 143.00, 145.40, 148.67, 170.15. HRMS (*m*/*z*): [M + H]^+^ calcd for C_18_H_15_N_3_O_2_S_2_Cl_2_, 440.0056; found, 440.0062.

#### 2-(2-(1-(4-(Methylsulfonyl)phenyl)ethylidene)hydrazinyl)-4-(2,4-difluorophenyl)thiazole (**2i**)

M.p. = 214.9 °C. ^1^H NMR (400 MHz, DMSO-*d*_6_): δ 2.38 (3H, s, CH_3_), 3.24 (3H, s, CH_3_), 7.20 (1H, t, *J* = 8.32 Hz, aromatic CH), 7.27 (1H, s, aromatic CH), 7.36–7.39 (1H, m, aromatic CH), 7.96 (2H, d, *J* = 7.64 Hz, 1,4-disubstituted benzene), 8.00 (2H, d, *J* = 8.60 Hz, 1,4-disubstituted benzene), 8.07 (1H, d, *J* = 8.24 Hz, aromatic CH). ^13^C NMR (100 MHz, DMSO-*d*_6_): δ 14.31 (CH_3_), 44.02 (CH_3_), 105.05, 109.23, 112.41, 119.54, 126.83, 127.65, 127.83, 129.51, 130.75, 130.90, 140.76, 143.03, 145.35, 169.41.

#### 2-(2-(1-(4-(Methylsulfonyl)phenyl)ethylidene)hydrazinyl)-4-(3-nitrophenyl)thiazole (**2j**)

M.p. = 290.2 °C. ^1^H NMR (400 MHz, DMSO-*d*_6_): δ 2.39 (3H, s, CH_3_), 3.25 (3H, s, CH_3_), 7.72 (1H, s, thiazole CH), 7.98–8.00 (4H, m, aromatic CH), 8.32–8.34 (2H, m, aromatic CH), 8.73 (1H, s, aromatic CH), 11.64 (1H, s, NH). ^13^C NMR (100 MHz, DMSO-*d*_6_): δ 14.50 (CH_3_), 44.02 (CH_3_), 107.76, 120.48, 122.52, 124.10, 126.86, 127.64, 128.03, 130.72, 132.01, 136.70, 140.84, 142.99, 148.77, 170.27. HRMS (*m*/*z*): [M + H]^+^ calcd for C_18_H_16_N_4_O_4_S_2_, 417.0686; found, 417.0691.

#### 2-(2-(1-(4-(Methylsulfonyl)phenyl)ethylidene)hydrazinyl)-4-(4-chlorophenyl) thiazole (**2k**)

M.p. = 235.1 °C. ^1^H NMR (400 MHz, DMSO-*d*_6_): δ 2.38 (3H, s, CH_3_), 3.24 (3H, s, CH_3_), 7.46–7.47 (2H, m, aromatic CH), 7.49 (1H, s, thiazole CH), 7.90 (2H, d, *J* = 7.52 Hz, 1,4-disubstituted benzene), 7.96 (2H, d, *J* = 7.80 Hz, 1,4-disubstituted benzene), 8.01 (2H, d, *J* = 8.12 Hz, 1,4-disubstituted benzene). ^13^C NMR (100 MHz, DMSO-*d*_6_): δ 14.50 (CH_3_), 44.02 (CH_3_), 105.83, 126.83, 127.64, 127.73, 129.13, 129.52, 132.44, 133.97, 140.74, 143.06, 145.29, 170.05. HRMS (*m*/*z*): [M + H]^+^ calcd for C_18_H_16_N_3_O_2_S_2_Cl, 406.0445; found, 406.0448.

#### 2-(2-(1-(4-(Methylsulfonyl)phenyl)ethylidene)hydrazinyl)-4-(2,4-dichlorophenyl)thiazole (**2l**)

M.p. = 211.3 °C. ^1^H NMR (400 MHz, DMSO-*d*_6_): δ 2.37 (3H, s, CH_3_), 3.24 (3H, s, CH_3_), 7.46 (1H, s, aromatic CH), 7.52 (1H, d, *J* = 8.52 Hz, aromatic CH), 7.70 (1H, s, thiazole CH), 7.93 (1H, s, aromatic CH), 7.96 (2H, d, *J* = 7.76 Hz, 1,4-disubstituted benzene), 8.01 (2H, d, *J* = 7.92 Hz, 1,4-disubstituted benzene), 11.59 (1H, s, NH). ^13^C NMR (100 MHz, DMSO-*d*_6_): δ 14.49 (CH_3_), 44.03 (CH_3_), 110.55, 126.82, 127.65, 127.96, 130.24, 132.05, 132.63, 132.69, 133.00, 140.75, 143.05, 145.23, 146.51, 169.14. HRMS (*m*/*z*): [M + H]^+^ calcd for C_18_H_15_N_3_O_2_S_2_Cl_2_, 440.0056; found, 440.0062.

#### 2-(2-(1-(4-(Methylsulfonyl)phenyl)ethylidene)hydrazinyl)-4-phenylthiazole (**2m**)

M.p. = 227.8 °C. ^1^H NMR (400 MHz, DMSO-*d*_6_): δ 2.38 (3H, s, CH_3_), 3.24 (3H, s, CH_3_), 7.30–7.33 (1H, m, aromatic CH), 7.39–7.44 (3H, m, aromatic CH), 7.89 (2H, d, *J* = 7.36 Hz, aromatic CH), 7.96 (2H, d, *J* = 7.80 Hz, 1,4-disubstituted benzene), 8.01 (2H, d, *J* = 7.72 Hz, 1,4-disubstituted benzene). ^13^C NMR (100 MHz, DMSO-*d*_6_): δ 14.33 (CH_3_), 44.03 (CH_3_), 104.97, 126.01, 126.80, 127.64, 128.05, 129.11, 135.08, 140.69, 143.12, 145.12, 150.81, 169.95. HRMS (*m*/*z*): [M + H]^+^ calcd for C_18_H_17_N_3_O_2_S_2_, 372.0835; found, 372.0835.

### Crystallographic Studies

The single-crystal X-ray diffraction measurements were carried out at 273.15 K on a Bruker D8 QUEST diffractometer with a rotation anode using graphite monochromated Mo Kα radiation at λ = 0.71073 Å. The data reduction was achieved with the Bruker SMART program.^40^ The crystal structure solution was performed using Olex2 software.^41^ Both structures were solved by least squares using the program SHEXL.^42^ All nonhydrogen atoms were refined anisotropically, and hydrogen atoms were added at calculated positions. The compounds’ molecular structure and geometrical parameters were acquired using the MERCURY program.^43^ Crystallographic data have been deposited with the Cambridge Crystallographic Data Center with CCDC No. 2411417.

### Carbonic Anhydrase I/II Inhibition Assay

The esterase method was used to investigate the effects of the molecule on hCA isoenzymes.^44^ This method is based on the activity of hCA for the hydrolysis of *p*-nitrophenyl acetate as a substrate for the reaction mechanism of *p*-nitrophenol or *p*-nitrophenolate. *P*-nitrophenol and *p*-nitrophenolate show the same absorbance at 348 nm. Since *p*-nitrophenyl acetate has very little absorption at this wavelength, it will be used as a blank. The *p*-nitrophenyl acetate substrate solution used in the experiments will be prepared daily.^45,46^

In this method, first, the cuvette contents were prepared as given in Table 1. Then, the 348 nm spectrophotometer device was zeroed by preparing the cuvette content in accordance with the blank. Then, the cuvette content containing enzymes, unlike the blank cuvette, was prepared according to Table 1 and placed in the spectrophotometer device. The absorbance at 348 nm was measured at the zeroth minute and the third minute. The absorbance difference between them gave the activity of the enzyme, that is, the control activity. Then, molecules at different concentrations were added one by one, the absorbances at 0 and 3 min were measured again, and their differences were taken.^45,46^ The absorbance differences obtained from different concentrations of molecules according to the control activity were compared. The enzyme activity values obtained were converted to % activity, their graphs were drawn according to the different concentrations of the molecules, and the IC_50_ value, that is, the concentration of molecules that reduced the enzyme activity by half, was found. The values were interpreted.

**Table 1:** Contents of a 1 mL Cuvette Used in Esterase Activity Studies for hCA Isoenzymes

substances used in the experiment	content of the control tube (blank) (μL)	sample tube contents (μL)
Tris-SO_4_ (0,5 M) pH value 7,4	400	400
*p*-nitrophenol acetate	360	360
H_2_O	240	220
enzyme solution		20
total final volume	1000	1000

### Molecular Docking

This study analyzed potentially effective compounds **2a** and **2h** by molecular docking toward the identified targets hCA I (PDB ID 1AZM)^47^ and hCA II (PDB ID 7RNZ)^48^ enzymes. As in the enzyme assays, acetazolamide was used as the standard compound. Docking was performed using the open-source SwissDock web server and AutoDock Vina^49^ for scoring. Docking results were analyzed and visualized with Discovery Studio Visualizer v24, ProteinsPlus structure-based modeling support server, and PyMOL v3 Molecular Graphics System. To prove the accuracy of the molecular docking procedures, redocking was performed on the cocrystal ligands of both proteins, and the root-mean-square deviation (RMSD) value was calculated separately.

### Molecular Dynamics Simulation Study

Compounds **2a** and **2h** were subjected to molecular dynamics simulations to evaluate their complexes’ dynamic properties and stability with target enzymes. These systems were prepared through the CHARMM-GUI^50^ server, and the simulations were carried out using Gromacs^51^ v2023 software and AMBER ff14SB force fields. The simulation box was set to a cubic geometry with a 10 Å buffer around the solute, and the TIP3P water model defined the solvents. To ensure electrical neutralization of the system, 0.15 M KCl ions were added, and the temperature was fixed at 310 K. In the initial stage, steric clashes and negative atomic interactions were eliminated by applying energy minimization. Then, equilibration procedures were performed in two different ensembles to bring the system to equilibrium. First, the system was brought to equilibrium using an NVT ensemble under constant temperature (*T*), constant volume (*V*), and constant atomic number (*N*) conditions, and then, the simulation was continued by switching to an NPT ensemble with constant temperature (*T*), constant pressure (*P*), and constant atomic number (*N*) conditions. After these preparatory steps, the production simulations were started with a time step of 0.002 ps, and the system was analyzed for 100 ns. Root-mean-square deviation (RMSD) and root-mean-square fluctuation (RMSF) were analyzed using graphs.

### ADMET Studies

For this study, the pharmacokinetic suitability of the targeted compounds was analyzed online using the SwissADME^52^ platform, and the findings were used to evaluate the potential of the candidate molecules in the drug development process (http://www.swissadme.ch/).

## Results and Discussion

### Chemistry

**Scheme 1:**
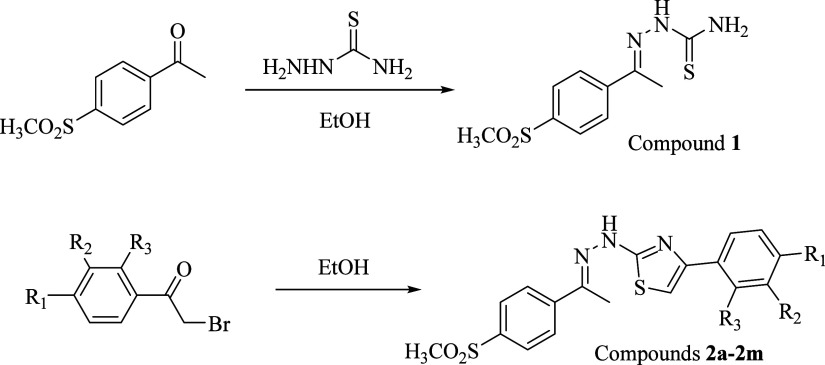
Synthesis Procedure for Obtaining Target Compounds (**2a**–**2m**)

A novel series of thiazole-methylsulfonyl derivatives was synthesized in a two-step strategy in Scheme 1. (4′-Methylsulfonyl) acetophenone was used as the starting precursor. It was refluxed with thiosemicarbazide in ethanol, and the resultant compound was treated with an appropriate 2-bromoacetophenone derivative to synthesize a series of thiazole-methylsulfonyl derivatives. The structures and functional groups of the synthesized compounds were confirmed using NMR spectroscopy, revealing the characteristic peaks for thiazole and methylsulfonyl moieties. HRMS analysis was performed to verify molecular weight, and the melting points of the synthesized compounds were checked for purity and stability determination.

In ^1^H NMR results, protons of methyl sulfonyl were detected as singlets between 3.24 and 3.25 ppm. The signals of the −CH_3_ proton were observed around 2.39 ppm. The protons on the thiazole ring were recorded between 7.20 and 7.80 ppm. The NH protons of compounds **2a**–**2m** appeared as a singlet signal at a range of 11.55–11.68 ppm. In the ^13^C NMR spectra, aliphatic carbons belonging to the methyl sulfonyl were observed in the range of 44.01–44.03 ppm. Methyl carbon atoms resonated at the δ range of 14.31–14.56 ppm.

### Crystallographic Studies

**Figure 2:**
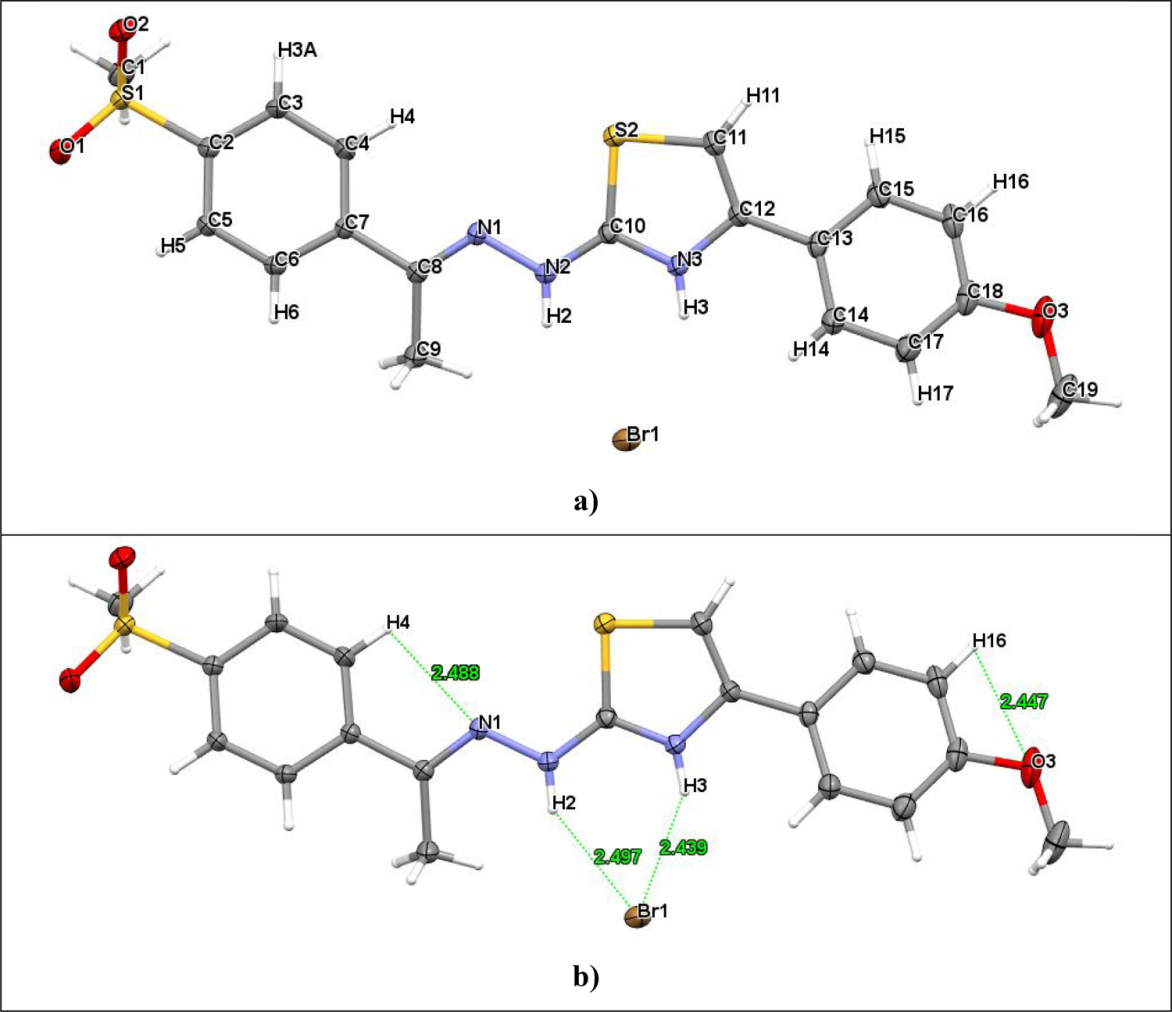
(a) Crystal structure for the HBr salt of compound **2b** showing 20% probability displacement ellipsoids and atom numbering scheme. (b) Inter- and intrahydrogen bonding interactions.

X-ray suitable crystals of compound **2b** were obtained by layer diffusion of diethyl ether into the methanolic solution of the compound after a few weeks. MERCURY structures of compounds are given in Figure 2. Crystal parameters, bond distances, and angles are given in Tables 2 and 3.

**Table 2:** Crystal Data and Structure Refinement for Compound **2b**

**compound**	**compound 2b**
CCDC number	2411417
empirical formula	C_19_H_19_N_3_O_3_S_2_·HBr
formula weight	482.41
temperature/K	273.15
crystal system	triclinic
space group	
*a*/Å	10.1474(5)
α/°	107.911(2)
*b*/Å	10.4862(5)
β/°	98.479(2)
*c*/Å	11.3119(6)
γ/°	103.891(2)
volume/Å^3^	1079.47(9)
*Z*	2
ρcalcg/cm^3^	1.484
μ/mm^–1^	2.122
*F*(000)	492.0
crystal size/mm^3^	0.05 × 0.02 × 0.01
2Θ range for data collection/°	5.038 to 56.75
index ranges	–13 ≤ *h* ≤ 13, −13 ≤ *k* ≤ 13, and −15≤ *l* ≤ 15
reflections collected	36,583
completeness to theta	28.375, 99.4%
absorption correction	multiscan
refinement method	full-matrix least squares on *F*^2^
independent reflections	5356, [Rint = 0.0744; Rsigma = 0.0741]
data/restraints/parameters	5356/0/257
goodness-of-fit on *F*^2^	1.007
final *R* indexes [*I* ≥ 2σ (*I*)]	*R*_1_ = 0.0460, *wR*_2_ = 0.0822
final *R* indexes [all data]	*R*_1_ = 0.1107, *wR*_2_ = 0.0985
largest diff. peak/hole/e Å^–3^	0.38/–0.39

**Table 3:** Selected Bond Distances (Å) and Angles (°) for Compound **2b**

**compound 2b**	
**bond distances (Å)**	**bond angles (°)**
S(1)–O(1) 1.438(2)	O(1)–S(1)–O(2) 117.85(1)
S(1)–O(2) 1.437(2)	O(2)–S(1)–C(1) 108.76(1)
S(1)–C(1) 1.741(4)	C(1)–S(1)–O(1) 108.41(1)
C(8)–N(1) 1.289(0)	C(7)–C(8)–N(1) 115.33(3)
C(10)–S(2) 1.703(0)	C(8)–N(1)–N(2) 116.61(2)
C(10)–N(3) 1.331(0)	N(1)–N(2)–C(10) 117.32(2)
N(1)–N(2) 1.382(0)	N(2)–C(10)–N(3) 121.56(2)
N(2)–C(10) 1.329(0)	C(10)–N(3)–C(12) 114.60(2)
S(2)–C(11) 1.745(0)	C(10)–S(2)–C(11) 88.98(1)
N(3)–C(12) 1.389(0)	N(3)–C(12)–C(13) 119.56(2)
C(18)–O(3) 1.366(0)	C(16)–C(18)–O(3) 114.96(3)
O(3)–C(19) 1.428(0)	C(18)–O(3)–C(19) 117.68(3)

X-ray data and spectral analysis were found to be consistent with the molecular structure of compound **2b**. Compound crystallized in the  space group of the triclinic system. The compound unit cell was composed of one complete molecule and a hydrogen bromide (HBr). Hydrogen bromide is bonded to nitrogen (N3) via the hydrogen (H3) atom to form the HBr salt of the compound. This structure is also consistent with the literature data.^53,54^ Unit cell dimensions are *a* = 10.1474(5) Å, α = 107.911(2)°, *b* = 10.4862(5) Å, β = 98.479(2)°, *c* = 11.3119(6) Å, γ = 103.891(2)°, and *V* = 1079.47(9) Å^3^. It was determined that the aromatic ring and the NH group were positioned opposite each other, and the molecule was stacked in the E isomer structure. The C=N double bond length (C(8)–N(1)) was measured as 1.289 Å, and the C–N single bond lengths of C(10)–N(2) and C(10)–N(3) were determined as 1.329 and 1.331 Å, respectively. The average length of the C–S bonds (C(10)–S(2) and S(2)–C(11)) in the thiazole ring was found to be 1.724 Å. The selected bond angles range between 88.98(1) and 121.56(2)°. In addition, some intra- and intermolecular hydrogen bonding interactions ranging from 2.439 to 2.497 Å were also observed.

### Carbonic Anhydrase I/II Inhibition Assay

CA enzymes play important roles in pH regulation, body fluid balance, calcification, lipogenesis, urea cycle, bicarbonate synthesis, and many other physiological events. In particular, CA inhibitors have been used clinically for antiglaucoma, diuretic, and antiepileptic treatments for the past 60 years.^4,55,56^

In this study, the esterase method determined the inhibitory effects of morin on the activities of hCA I and hCA II isoenzymes purified from human erythrocytes under *in vitro* conditions. The principle of this method is based on the hydrolysis of *p*-nitrophenylacetate used as a substrate in the presence of the CA enzyme to *p*-nitrophenol or *p*-nitrophenolate at 348 nm.^46^ For the inhibition study, five different inhibitor concentrations of molecules were used at a fixed substrate concentration, and the inhibitor concentration (IC_50_) values that reduced the enzyme’s activity by half were determined by drawing % activity–[*I*] graphs. Although various antiepileptic drugs target the inhibition of hCA enzymes, these drugs have many side effects. In this context, discovering new synthetic drugs that inhibit hCA are specific, selective, and more effective is important.^57−60^

**Figure 3:**
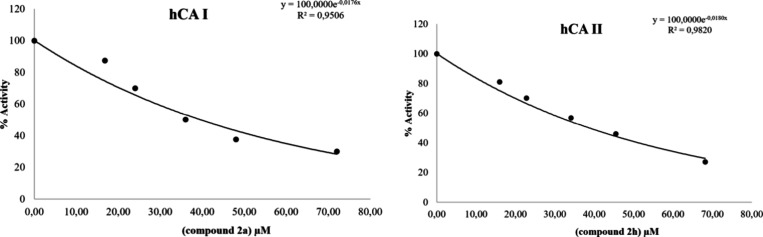
% Activity–inhibitor concentration graphs of the best inhibiting compounds.

The effects of the molecule and the common medicine acetazolamide molecule on the carbonic anhydrase I and II (hCA I–II) isoenzymes were investigated *in vitro* using the esterase method. Graphs showing the variations in percentage activity–inhibitory drug concentrations were produced, and IC_50_ values were calculated (Table 4 and Figure 3). The IC_50_ values were found between 39.38 and 198.04 μM (AAZ IC_50_ = 18.11 μM) for hCA I and 39.16–86.64 μM (AAZ IC_50_ = 20.65 μM). These results showed that they had less inhibition than the standard medication, although they had an inhibitory potential.

**Table 4:** IC_50_ Values of Molecules that Inhibit hCA I and II Isoenzymes

	**IC**_**50**_**(μM)**						
**comp.**	**hCA I**	***r***^**2**^	**hCA II**	***r***^**2**^	**R**_**1**_	**R**_**2**_	**R**_**3**_
**2a**	39.38	0.9506	70.01	0.9885	–NO_2_	–H	–H
**2b**	59.75	0.9224	76.17	0.9439	–OCH_3_	–H	–H
**2c**	63.01	0.9654	39.16	0.9381	–CN	–H	–H
**2d**	103.45	0.9556	54.15	0.9756	–F	–H	–H
**2e**	77.88	0.9268	40.53	0.9748	–phenyl	–H	–H
**2f**	133.29	0.9212	51.34	0.8971	–Br	–H	–H
**2g**	198.04	0.9058	78.76	0.9936	–CH_3_	–H	–H
**2h**	60.80	0.8464	38.50	0.9820	–Cl	–Cl	–H
**2i**	52.91	0.9828	86.64	0.9459	–F	–H	–F
**2j**	80.59	0.9511	64.78	0.9411	–H	–NO_2_	–H
**2k**	58.74	0.9702	86.64	0.9613	–Cl	–H	–H
**2l**	67.95	0.9629	71.45	0.8974	–Cl	–H	–Cl
**2m**	44.43	0.9623	53.31	0.9559	–H	–H	–H
**AAZ**	18.11	0.9387	20.65	0.9756			

According to the results presented in Table 4, compound **2a** exhibited the highest inhibitory potential, with an IC_50_ value of 39.38 μM for hCA I, and compound **2h** exhibited the highest inhibitory potential, with an IC_50_ value of 38.50 μM for hCA II. When the inhibition potentials of other molecules were examined according to the IC_50_ data in Table 4, it was seen that they inhibited the enzymes, although lower than the standard substance AAZ.

When the structures of the compounds are examined, it is seen that they are derived using different substituents on the phenyl ring. When the carbonic anhydrase I/II inhibition result was evaluated, it was seen that hCA I inhibitory activity increased with the electron-withdrawing group at the *para* position of the phenyl ring (compound **2a**). It was determined that the activity decreased when the nitro group was in the *meta* position (compound **2j**). It was concluded that the nitro group at the *para* position is important for hCA I inhibitory activity. For hCA II inhibitory activity, the presence of the 3,4-dichloro substituent on the phenyl ring increased the activity (compound **2h**). While low activity was observed in compound **2k** with chlorine substitution at the *para* position, the activity increased slightly with the second chlorine at the *ortho* position (compound **2l**). However, it is seen that the activity increases significantly with the introduction of the chlorine substituent in the *meta* position (compound **2h**).

These results are further supported by the graphical depiction of enzyme activity vs inhibitor concentration (Figure 3), which makes it evident that hCA I and II activity decreases concentration-dependently as inhibitor concentration rises. The relative inhibitory power of each chemical is indicated by the trend in the IC_50_ values, with compounds **2a** and **2h** exhibiting the highest inhibition and compounds **2g**, **2i**, and **2k** the weakest. The table’s *r*^2^ values further support the validity of the IC_50_ values by showing a strong match between the experimental data and the anticipated dose–response curve.

The findings imply that every examined molecule has some level of hCA inhibitory activity, which may be useful in the development of novel medications to treat cholinergic dysfunction-related disorders like epilepsy. The mechanism of inhibition, pharmacokinetic characteristics, and *in vivo* effectiveness of these drugs require more investigation.

In conclusion, because they showed the highest hCA inhibition, compounds **2a** and **2h** are the most promising candidates for more research. Future investigations into the possible development of these chemicals as epilepsy treatment agents are made possible by this study.

### Molecular Docking

**Figure 4:**
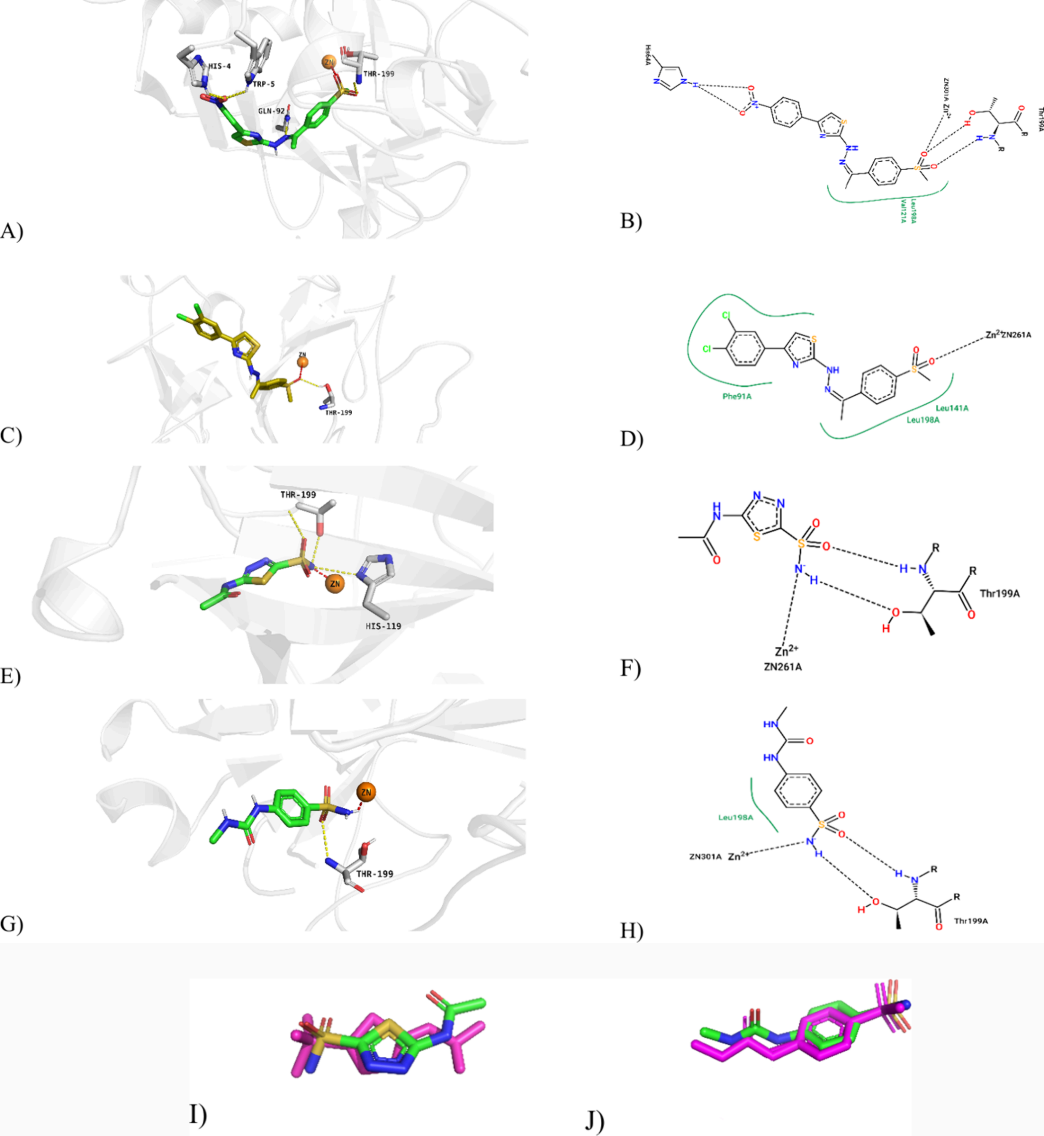
2D and 3D ligand–enzyme interaction profile; (A, B) **2a**-hCA I, (C, D) **2h**-hCA II, (E, F) AZM-hCA I, and (G, H) 64W-hCA II. Comparative representations obtained by superimposing the cocrystallized pose of the ligand with its resident conformation in the active sites of the proteins: (I) 1AZM and (J) 7RNZ. The magenta color represents the X-ray crystallized pose.

The analysis revealed that compounds **2a** and **2h** exhibited stronger binding energies and stable interaction networks compared to the other compounds studied in this work. The RMSD value for the hCA I cocrystal was found to be 1.4 Å and 0.9 Å for the hCA II cocrystal as a result of the reassembly analysis performed to evaluate the accuracy of the molecular docking process. Compound **2a** has a docking score of −7.41 kcal/mol in hCA I protein and −7.75 kcal/mol in hCA II protein. Compound **2h** stands out as one of the compounds with the strongest binding energies with values of −8.467 kcal/mol in hCA I protein and −7.653 kcal/mol in hCA II protein. Coordination with Zn^2+^ ion played a critical role in the interaction with the protein active site, strengthening the binding and reinforcing the inhibitory properties of these compounds. Compound **2a** formed a hydrogen bond and π–π stacked interaction with His64 and hydrophobic interaction with Val62 in their interaction with hCA I protein. It also provided strong coordination by forming a hydrogen bond with the amino acid Thr199 and a metallic bond with Zn^2+^. It also formed hydrophobic interactions with the amino acids Phe91, Ala121, Leu198, and His94. In its interactions with the HCA II protein, it formed hydrogen bonds with Gln92, Trp5, and His4 and coordinated with Zn^2+^. This strong and stable binding structure significantly enhanced the inhibitory properties of compound **2a**. Compound **2h** formed hydrogen bonds with amino acids Thr199 in interactions with HCA I protein and exhibited hydrophobic interactions with Leu198, Ala135, Phe91, Leu131, and Ala121. In interactions with HCA II protein, it showed hydrogen bonding with Thr199, π–π stacking interaction with His94, and hydrophobic binding with amino acids Leu198, Val121, Phe131, Pro202, and Trp5. Moreover, metallic interactions with Zn^2+^ further strengthened the binding stability of compound **2h**. The binding positions of the compounds are presented in Figure 4. The fact that the cocrystals exhibited higher inhibitory potential despite showing fewer interactions in the enzyme pocket compared to **2a** and **2h** is most likely due to the superiority of the strong chelation effect of the NH group with Zn^2+^ ions over the weak coordination ability of sulfone oxygen, which is consistent with the critical role of metal ion chelation on affinity and activity.^61^

*In silico* analyses showed that electron-withdrawing groups (e.g., −NO_2_ and −CN) in the *para* position added to the phenyl group at the fourth position of the thiazole ring enhanced hCA I inhibition, while the −NO_2_ group in the *meta* position decreased the activity. In terms of hCA II, it was determined that the 3,4-dichloro substituent provided the highest inhibition by establishing optimal hydrophobic interactions, while large halogens (e.g., *para*-bromine) decreased the activity due to steric hindrance.

### Molecular Dynamics Simulation

**Figure 5:**
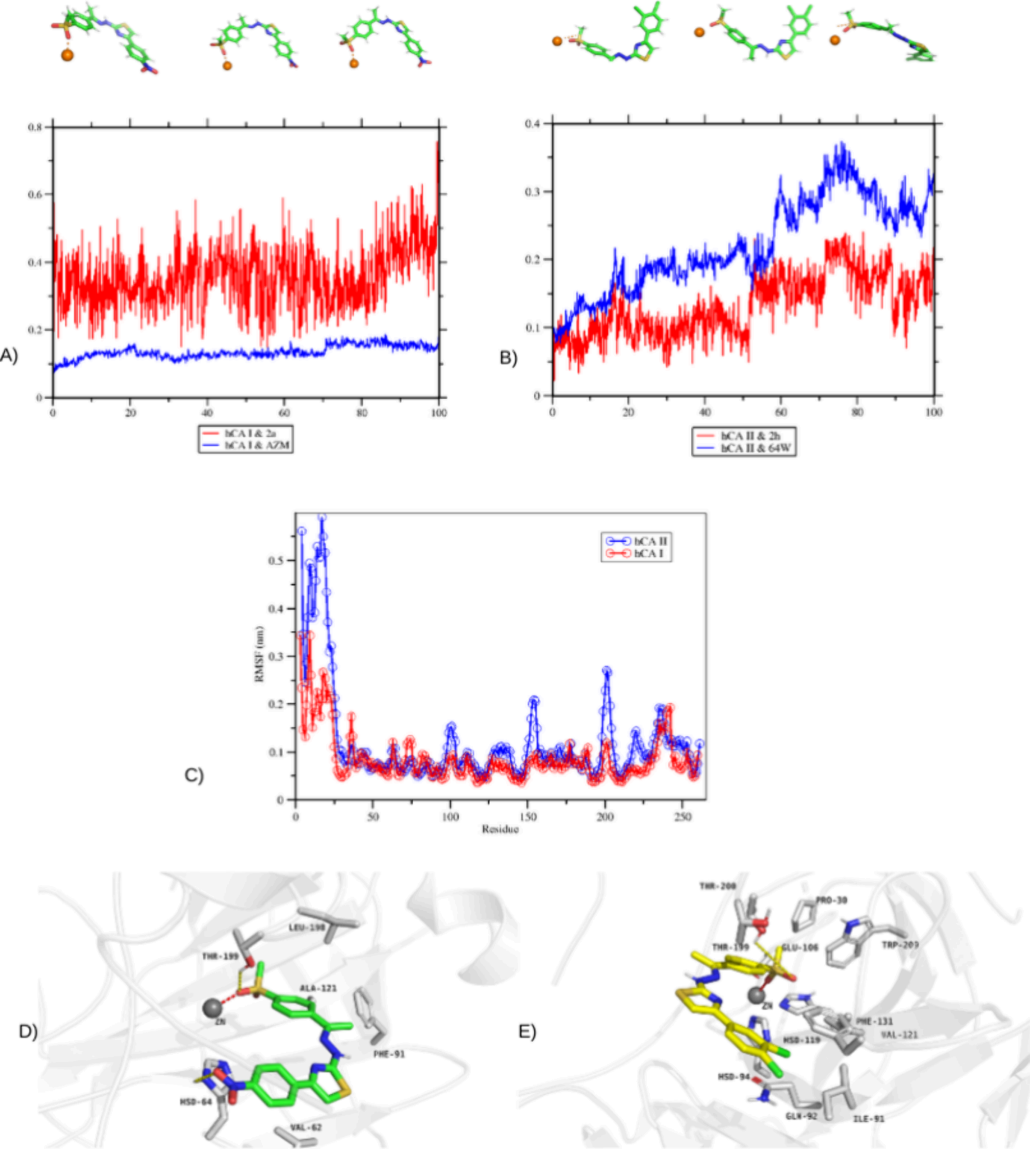
Molecular dynamics trajectory analysis. (A) RMSD changes of hCA I protein (blue) and ligand (red). (B) RMSD changes of hCA II protein (blue) and ligand (red). Both graphs visualize the dynamic behavior of proteins and ligands over 100 ns. (C) RMSF plot of hCA I (red) and hCA II (blue) proteins showing residue-wise flexibility changes. (D, E) Protein–ligand (**2a** and **2h**, respectively) contact analysis of the MD trajectory.

The stability of the protein–ligand complex during MD simulations was studied in detail by RMSD and RMSF analyses. Ligand stability at enzyme active sites visualized the behavior of the RMSD values, which were calculated for compounds **2a**, **2h**, and cocrystals fit protein. RMSD plots (Figure 5A,B) of the hCA I and AZM and hCA II and 64W complexes exhibited a stable structure after a short initial tuning period. While the RMSD values of the hCA I and AZM complex fluctuated around 1 Å, they reached a more stable profile after 20 ns and remained in this range until the end of the simulation. Similarly, the RMSD values of the hCA II and 64W complex stabilized in the range of 2–3.5 Å after initial fluctuations. RMSD analysis of the ligand–protein complexes showed that the stabilization was maintained after the initial fluctuations. In particular, hCA II and **2h** exhibited a constant value of about 1.8 Å after 50 ns, indicating a stable ligand positioning in the binding site. These results emphasize the ligands’ dynamic stability and compatibility in the binding site. The RMSF analysis results (Figure 5C) reveal the levels of flexibility in different regions of both proteins. The catalytic regions of hCA I and hCA II were shown to be structurally conserved with low RMSF values. In contrast, higher RMSF values were recorded at some loop regions on the surface and these regions exhibited more mobility throughout the simulation. This mobility may be due to the flexibility associated with ligand-binding sites. The data obtained confirm that the ligands retain their basic interactions throughout the simulation and occupy a stable position in the binding site. The interactions of compounds **2a** and **2h** with the Zn^2+^ complex show that the system maintains stability throughout the simulation. In addition, as shown in Figure 5D,E, the hCA I and **2a** complex interacted with Ala121, His94, Thr199, and Val62 residues, and these bonds remained stable throughout the simulation. The hCA II and **2h** complex also showed hydrogen bonding and hydrophobic interactions with similar amino acid residues. Interestingly, the bonding pattern of the compound **2h** changed at the beginning of the simulation. While hydrogen initially bonded through the O1 atom attached to the sulfonyl group, it started hydrogen bonding with the O2 atom after 100 ns. These bonds remained intact and stable throughout the simulation, confirming the reliability of the docking results. In both docking and MD results, it was observed that the bonds between amino acid residues and ligands exhibited close or identical lengths. For example, a stable hydrophobic interaction between Leu198 and compound **2a** was found throughout the simulation.

In conclusion, the findings obtained from 100 ns MD simulations confirm the stable binding of the selected compounds to the hCA I and hCA II enzymes. The stability and dynamic behaviors observed in the simulations agree with docking analyses. This study highlights the potentially inhibitory activities of the compounds and provides an important basis for further biomolecular design studies.

### ADMET Results

Predicting pharmacokinetic parameters in the drug discovery process is important in identifying potential drug candidates. In this context, the study of ADME (absorption, distribution, metabolism, and excretion) properties of newly developed molecules by computational methods can reduce the risk of failure in the early stages of the drug design process. This study evaluated the pharmacokinetic properties of compounds **2a** and **2h** based on ADME parameters. The lipophilicity level was determined as log *P* o/w values of 3.05 for compound **2a** and 4.74 for compound **2h**. Both compounds were found to have low gastrointestinal absorption potential and did not show the ability to cross the blood–brain barrier. When the metabolic effects were analyzed, both compounds inhibited CYP2C19 and CYP2C9 enzymes, but they did not affect CYP1A2 and CYP2D6 enzymes. Furthermore, both compounds have the potential to inhibit the CYP3A4 enzyme. Log *K*_p_ values calculated for skin permeability were −6.11 cm/s for compound **2a** and −5.24 cm/s for compound **2h**. This result shows that compound **2h** has a higher potential for skin permeability than compound **2a**. In addition, the observed properties indicate that both molecules conform to Lipinski’s rule of five, supporting their potential for oral bioavailability with drug similarity. These results provide an analysis of the pharmacokinetic properties of the compounds and support their potential use in the drug development process.

## Conclusions

In this study, a novel series of thiazole-methylsulfonyl derivatives was successfully designed and synthesized, and their potential was determined against human carbonic anhydrase enzymes I and II. The target compounds were characterized by ^1^H NMR, ^13^C NMR, and HRMS. Additionally, the structure of compound **2b** was elucidated by an X-ray study. Compound **2a** demonstrated potent inhibition of hCA I, with an IC_50_ value of 39.38 μM. Similarly, compound **2h** showed significant inhibition of hCA II, with an IC_50_ value of 38.50 μM. Docking results revealed specific interactions with the active sites of hCA I and hCA II, which was supported by the stability observed in dynamic simulations. According to molecular docking, compounds **2a** and **2h** were found to fit into the active pocket region of the protein, establish similar interactions with Phe91, Ala121, Leu198, His94, and Thr199, the amino acid residues with which the cocrystal compounds interact, and provide a strong binding affinity through zinc–ligand interaction. Furthermore, the low inhibitory activity of compound **2h** despite the high docking score for hCA I can be explained by the weakness in its binding kinetics and binding in a conformation that cannot effectively block the substrate binding site. In addition, the ADME properties of these compounds were analyzed and found to exhibit appropriate pharmacokinetic profiles and druglike properties. This study highlights the importance of thiazole-methylsulfonyl derivatives in inhibiting carbonic anhydrase enzymes with possible therapeutic applications.

## Supporting Information Available

Crystallographic information for C_19_H_19_BrN_3_O_3_S_2_ (CIF)
^1^H NMR, ^13^C NMR, and HRMS spectra of **2a**–**2m** compounds (PDF)

## Supplementary Material

ao5c00509_si_001.cif
