# Supplementary material for: Synthesis of Thiazole-methylsulfonyl Derivatives, X-ray Study, and Investigation of Their Carbonic Anhydrase Activities: In Vitro and In Silico Potentials
Source: ACS Omega. 2025 Mar 27;10(13):13583–94. doi: 10.1021/acsomega.5c00509 (PMC11983343; doi:10.1021/acsomega.5c00509)
Supplement: Supplementary file 2 — ao5c00509_si_002.pdf [file ao5c00509_si_002.pdf]

Synthesis of thiazole-methylsulfonyl derivatives, X-Ray study and investigation of their carbonic anhydrase activities: *in vitro* and *in silico* potentials

Zahra Maryam[1], Ayşen Işık[2], Emine Rana Bağcı[3,7], Maksut Yıldız[4], Hakan Ünver[5], Ümit M. Kocyigit[4], Burak Kırılmaz[6], Ismail Celik[6], Ulviye Acar Çevik[1], Yusuf Özkay[1*], Zafer Asım Kaplancıklı[1,8]

[1]Department of Pharmaceutical Chemistry, Faculty of Pharmacy, Anadolu University, Eskişehir 26470, Turkey.

[2]Department of Biochemistry, Faculty of Science, Selçuk University, 42250 Konya, Turkey.

[3]Department of Pharmaceutical Chemistry, Faculty of Pharmacy, Afyonkarahisar Health Sciences University, Afyonkarahisar 03030, Turkey.

[4]Department of Biochemistry, Faculty of Pharmacy, Cumhuriyet University, Sivas 58140, Turkey.

[5]Department of Chemistry, Faculty of Science, Eskisehir Technical University, Eskisehir 26470, Turkey.

[6]Department of Pharmaceutical Chemistry, Faculty of Pharmacy, Erciyes University, Kayseri 38039, Turkey.

* Corresponding Author. E-mail: yozkay@anadolu.edu.tr; Tel. +90-222-335-0580/3775
Address: Anadolu University, Faculty of Pharmacy, Department of Pharmaceutical Chemistry, 26470, Eskişehir, Turkey.

[7] Department of Pharmaceutical Chemistry, Graduate School, Anadolu University, Eskişehir, Turkey.
[8]The Rectorate of Bilecik Şeyh Edebali University, Bilecik 11230, Turkey

Author Information

Corresponding Authors

Yusuf Özkay-Department of Pharmaceutical Chemistry, Faculty of Pharmacy, Anadolu University, Eskişehir 26470, Turkey. E-mail; yozkay@anadolu.edu.tr

Authors

Zahra Maryam- Department of Pharmaceutical Chemistry, Faculty of Pharmacy, Anadolu University, Eskişehir 26470, Turkey. E-mail; zahra.maryam489@gmail.com

Ayşen Işık- Department of Biochemistry, Faculty of Science, Selçuk University, 42250 Konya, Turkey. E-mail; isik.aysen@selcuk.edu.tr

Emine Rana Bağcı- Department of Pharmaceutical Chemistry, Faculty of Pharmacy, Afyonkarahisar Health Sciences University, Afyonkarahisar 03030, Turkey; Department of Pharmaceutical Chemistry, Graduate School, Anadolu University, Eskişehir 26470, Turkey. E-mail; rana.bagci@afsu.edu.tr

Maksut Yıldız Department of Biochemistry, Faculty of Pharmacy, Cumhuriyet University, Sivas 58140, Turkey. E-mail: maske58@gmail.com

Hakan Ünver- Department of Chemistry, Faculty of Science, Eskisehir Technical University, Eskisehir 26470, Turkey. E-mail: hakanunver@eskisehir.edu.tr

Ümit M. Kocyigit- Department of Biochemistry, Faculty of Pharmacy, Cumhuriyet University, Sivas 58140, Turkey. E-mail: ukocyigit@cumhuriyet.edu.tr

Burak Kırılmaz- Department of Pharmaceutical Chemistry, Faculty of Pharmacy, Erciyes University, Kayseri 38039, Turkey; E-mail; burakkirilmaz@erciyes.edu.tr

İsmail Celik-Department of Pharmaceutical Chemistry, Faculty of Pharmacy, Erciyes University, Kayseri 38039, Turkey; E-mail; ismailcelik@erciyes.edu.tr

Ulviye Acar Çevik-Department of Pharmaceutical Chemistry, Faculty of Pharmacy, Anadolu University, Eskişehir 26470, Turkey. E-mail; uacar@anadolu.edu.tr

Zafer Asım Kaplancıklı- Department of Pharmaceutical Chemistry, Faculty of Pharmacy, Anadolu University, Eskişehir 26470, Turkey; The Rectorate of Bilecik Şeyh Edebali University, Bilecik 11230, Turkey. E-mail; zakaplan@anadolu.edu.tr

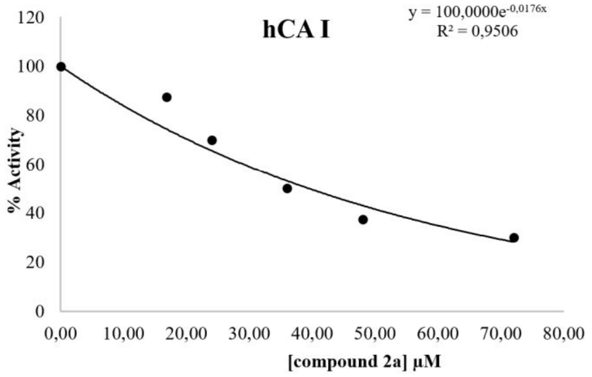
hCA I
y = 100,0000e-0,0176x
R² = 0,9506
% Activity
[compound 2a] µM

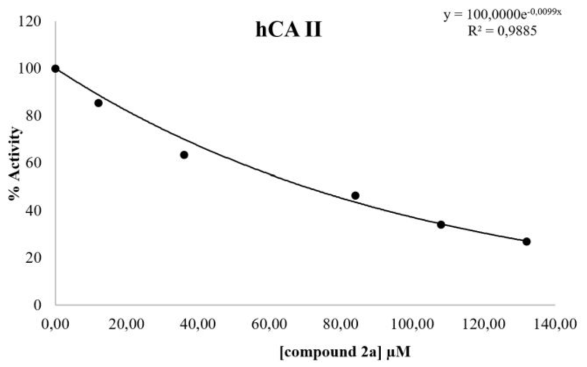
hCA II
y = 100,0000e-0,0099x
R² = 0,9885
% Activity
[compound 2a] µM

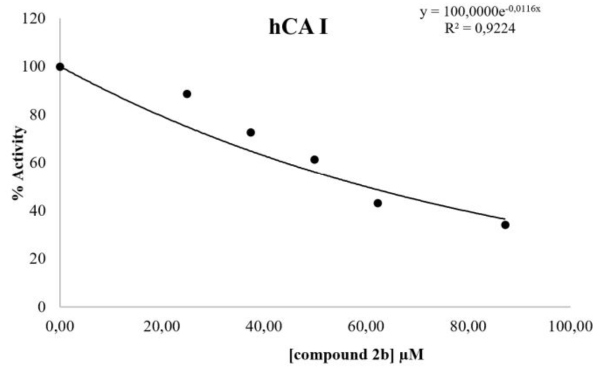
hCA I
y = 100,0000e-0,0116x
R² = 0,9224
% Activity
[compound 2b] µM

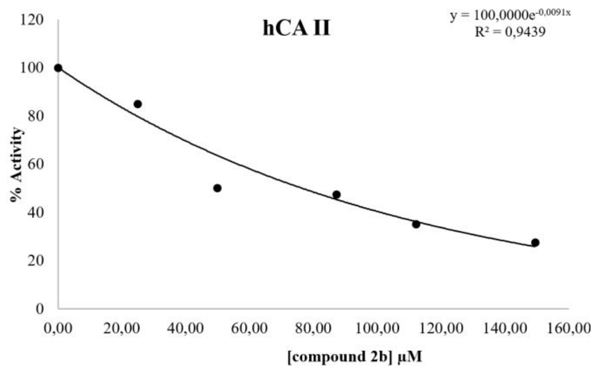
hCA II
y = 100,0000e-0,0091x
R² = 0,9439
% Activity
[compound 2b] µM

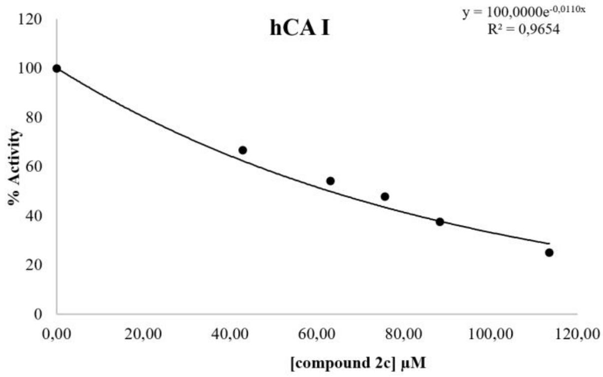
hCA I
y = 100,0000e-0,0110x
R² = 0,9654
% Activity
[compound 2c] µM

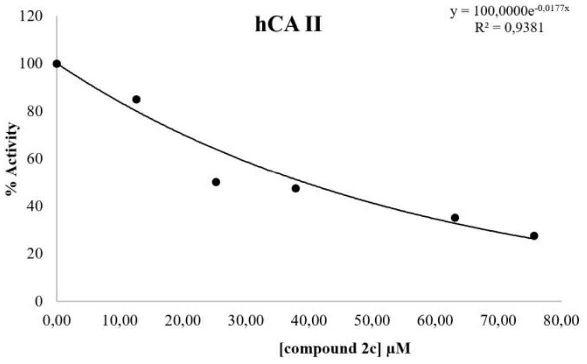
hCA II
y = 100,0000e-0,0177x
R² = 0,9381
% Activity
[compound 2c] µM

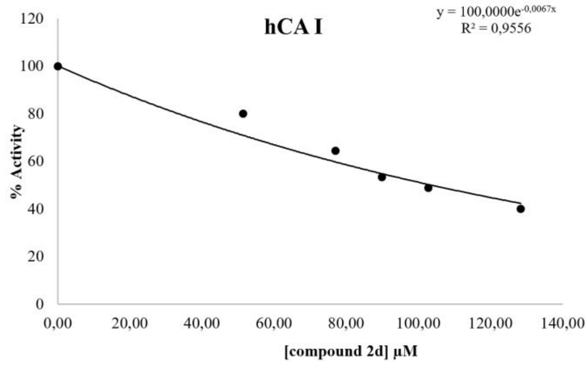
hCA I
y = 100,0000e-0,0067x
R² = 0,9556
% Activity
[compound 2d] µM

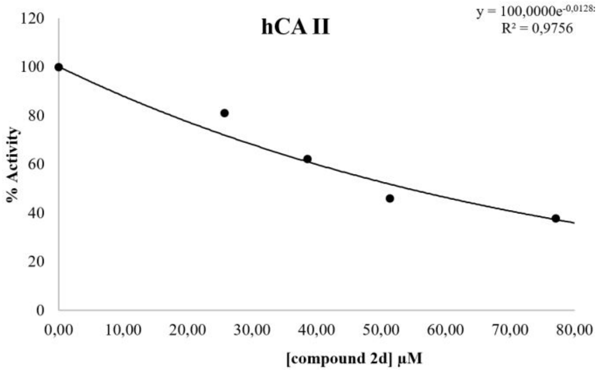
hCA II
y = 100,0000e-0,0128
R² = 0,9756
% Activity
[compound 2d] µM

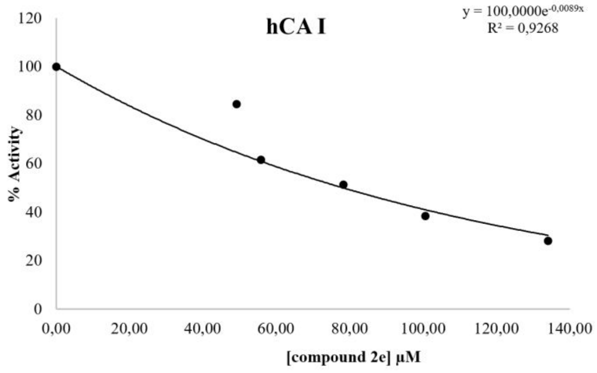
hCA I
y = 100,0000e-0,0089x
R² = 0,9268
% Activity
[compound 2e] µM

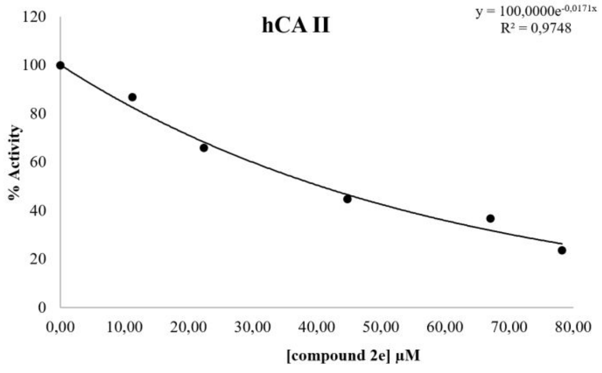
hCA II
y = 100,0000e-0,0171x
R² = 0,9748
% Activity
[compound 2e] µM

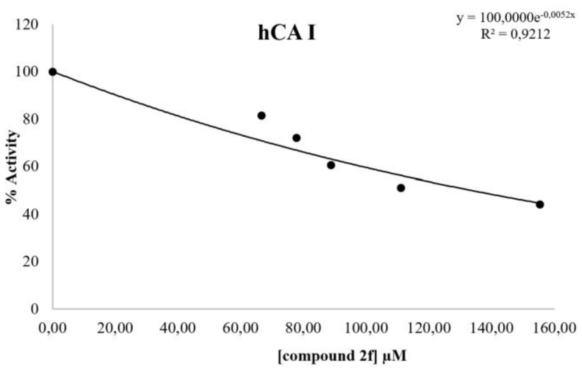
hCA I
y = 100,0000e-0,0052x
R² = 0,9212
% Activity
[compound 2f] µM

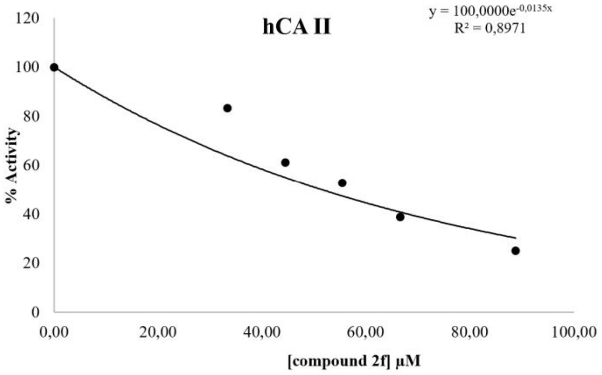
hCA II
y = 100,0000e-0,0135x
R² = 0,8971
% Activity
[compound 2f] µM

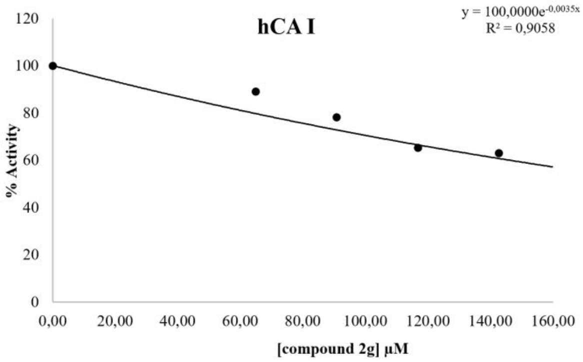
hCA I
y = 100,0000e-0,0035x
R² = 0,9058
% Activity
[compound 2g] µM

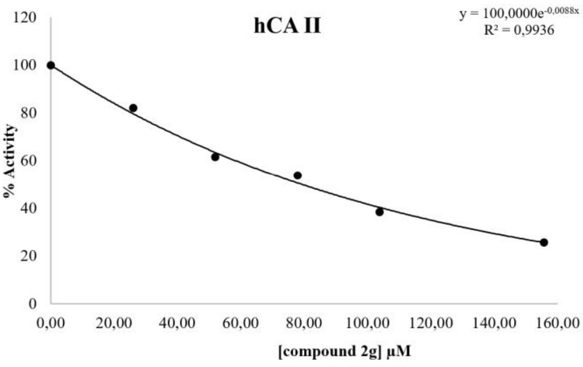
hCA II
y = 100,0000e-0,0088x
R² = 0,9936
% Activity
[compound 2g] µM

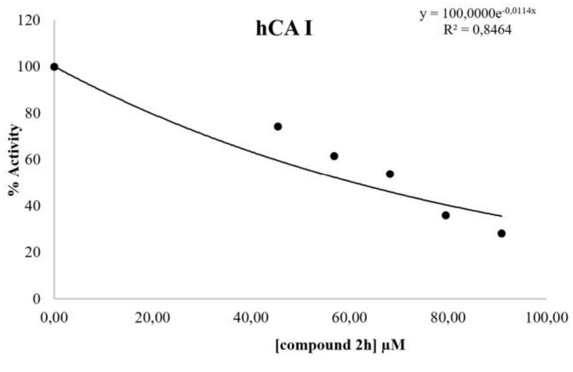
hCA I
y = 100,0000e-0,0114x
R² = 0,8464
% Activity
[compound 2h] µM

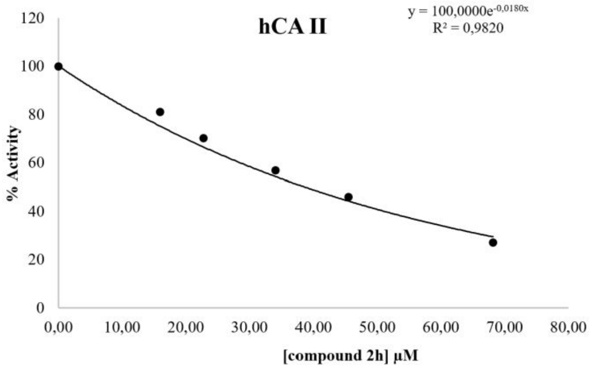
hCA II
y = 100,0000e-0,0180x
R² = 0,9820
% Activity
[compound 2h] µM

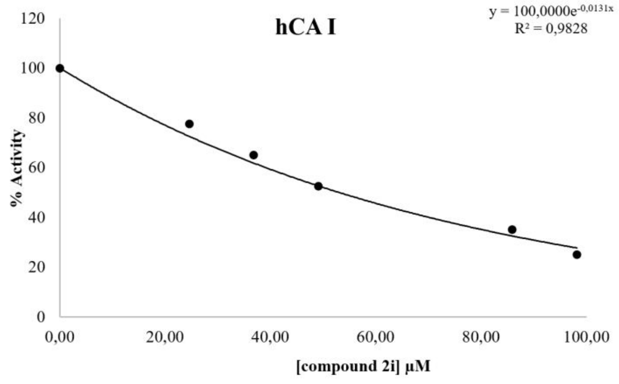
hCA I
y = 100,0000e-0,0131x
R² = 0,9828
% Activity
[compound 2i] µM
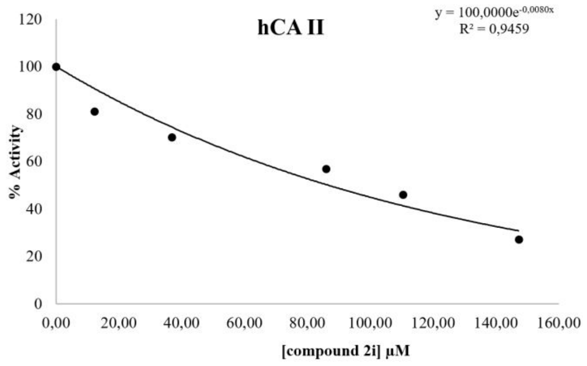
hCA II
y = 100,0000e-0,0080x
R² = 0,9459
% Activity
[compound 2i] µM
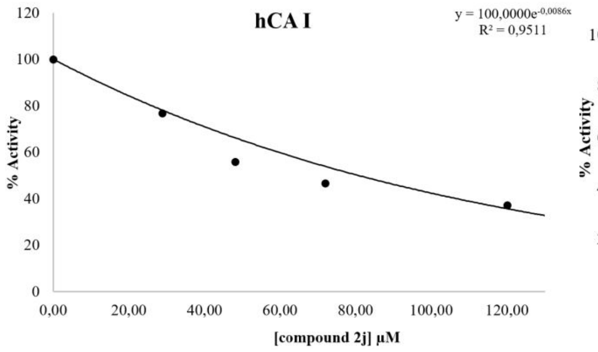
hCA I
y = 100,0000e-0,0086x
R² = 0,9511
% Activity
[compound 2j] µM
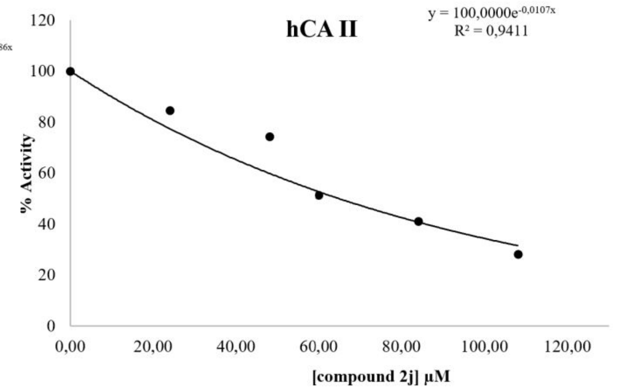
hCA II
y = 100,0000e-0,0107x
R² = 0,9411
% Activity
[compound 2j] µM
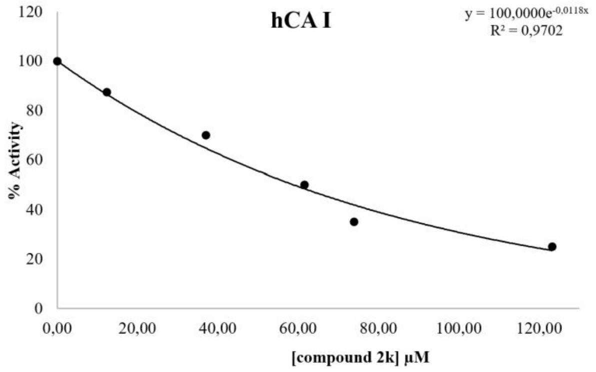
hCA I
y = 100,0000e-0,0118x
R² = 0,9702
% Activity
[compound 2k] µM
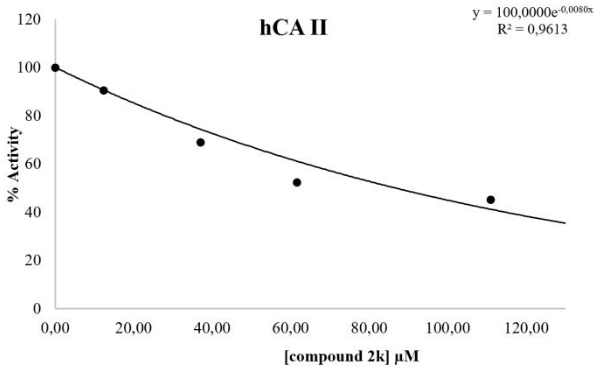
hCA II
y = 100,0000e-0,0080x
R² = 0,9613
% Activity
[compound 2k] µM
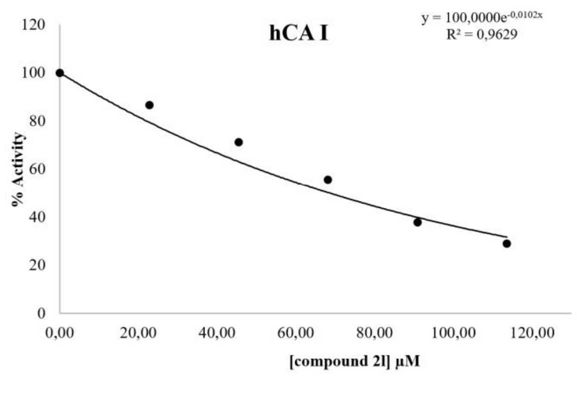
hCA I
y = 100,0000e-0,0102x
R² = 0,9629
% Activity
[compound 2l] µM
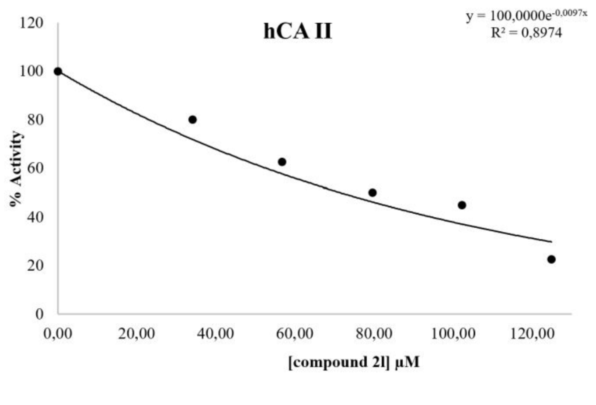
hCA II
y = 100,0000e-0,0097x
R² = 0,8974
% Activity
[compound 2l] µM

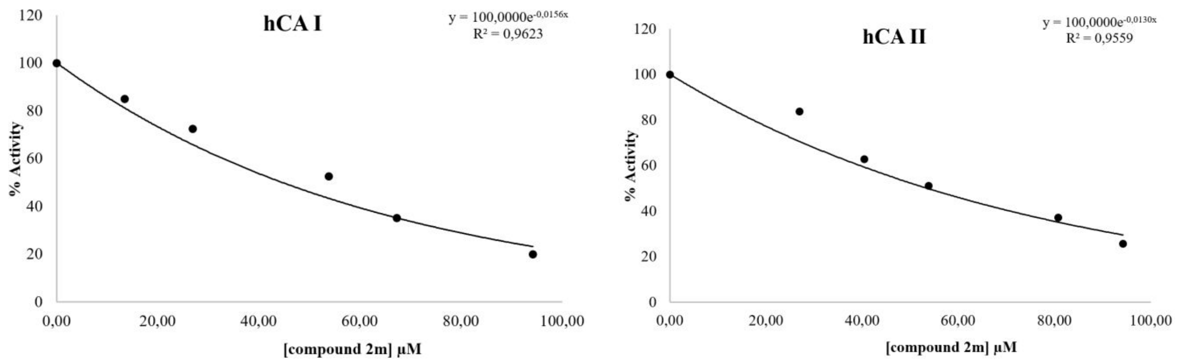

Figure S1. % Activity - inhibitor concentration graphs of the compounds **2a-2m**.

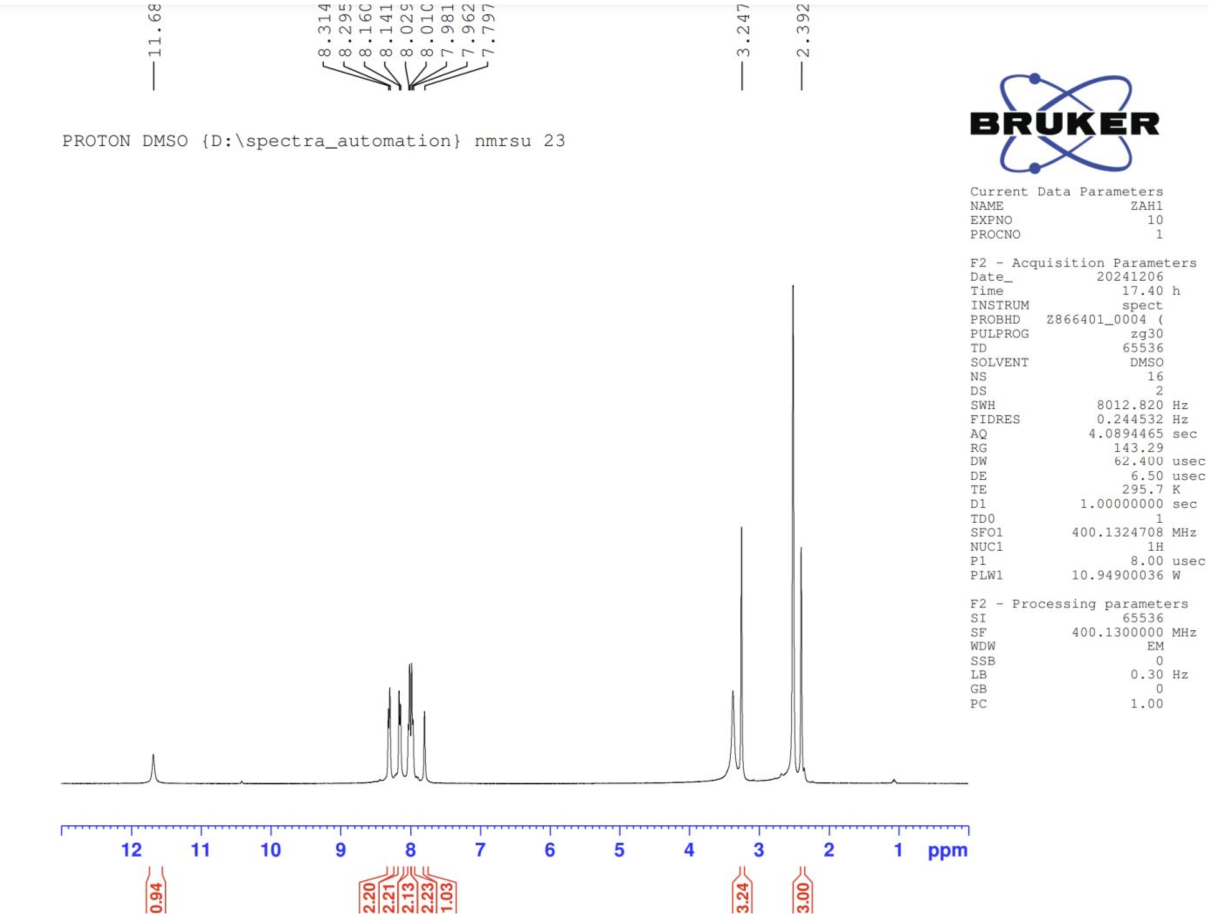

Figure S2. ^{1}H-NMR spectrum of compound **2a**

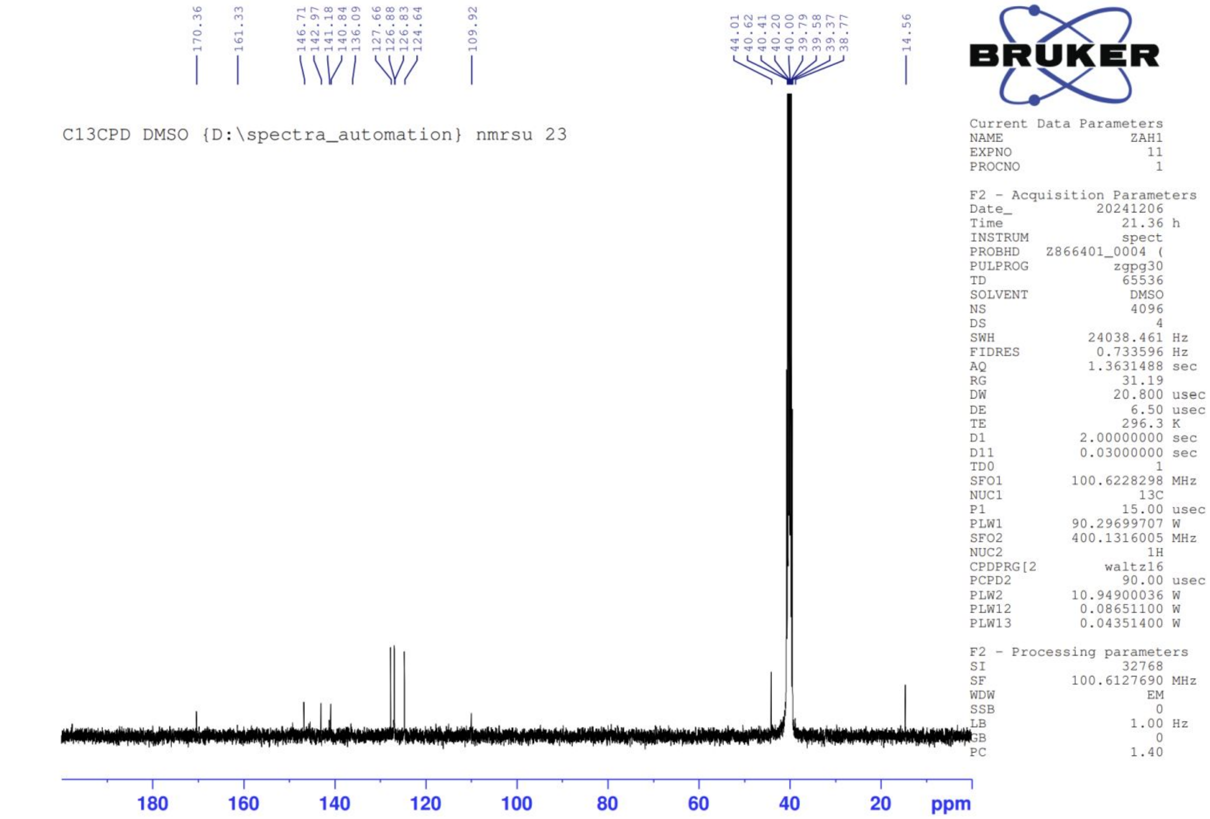

Figure S3. ^{13}C-NMR spectrum of compound **2a**

Data File: C:\LabSolutions\Data\Analiz\uac\ZAH-1_95.lcd

Elmt	Val.	Min	Max	Elmt	Val.	Min	Max	Elmt	Val.	Min	Max	Elmt	Val.	Min	Max	Use Adduct
H	1	5	40	O	2	0	4	S	2	2	2	Ru	2	0	0	H
C	4	5	30	F	1	0	0	Cl	1	0	0	Pd	2	0	0	
N	3	0	5	P	3	0	0	Br	1	0	0	I	3	0	0	

Error Margin (ppm): 10
HC Ratio: unlimited
Max Isotopes: 3
MSn Iso RI (%): 10.00

DBE Range: 12.0 - 25.0
Apply N Rule: yes
Isotope RI (%): 1.00
MSn Logic Mode: AND

Electron Ions: both
Use MSn Info: yes
Isotope Res: 9000
Max Results: 200

Event#: 1 MS(E+) Ret. Time : 3.453 Scan# : 519

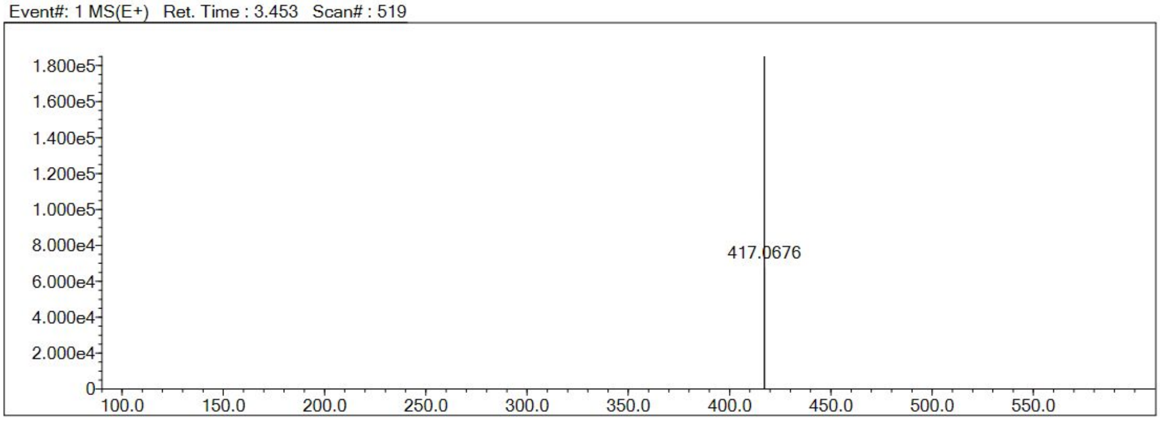

Measured region for 417.0676 m/z

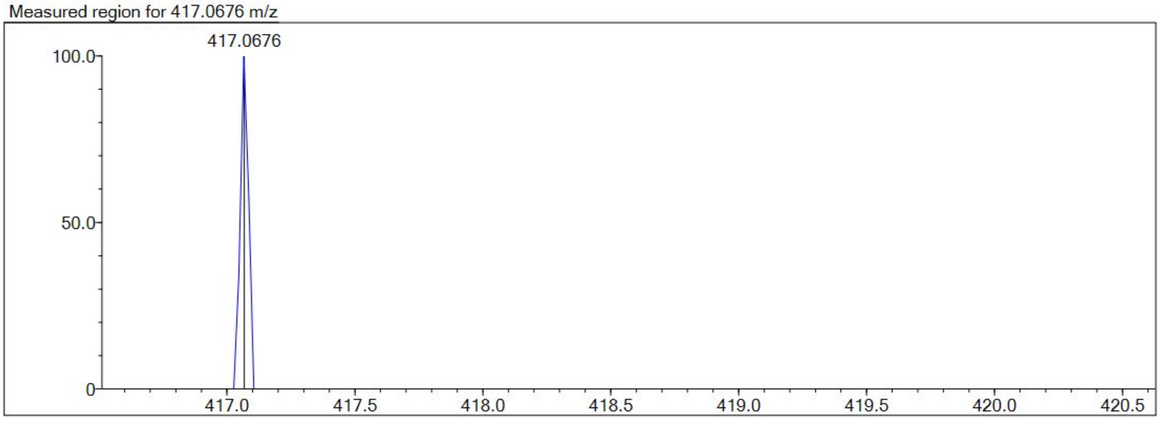

C18 H16 N4 O4 S2 [M+H]+ : Predicted region for 417.0686 m/z

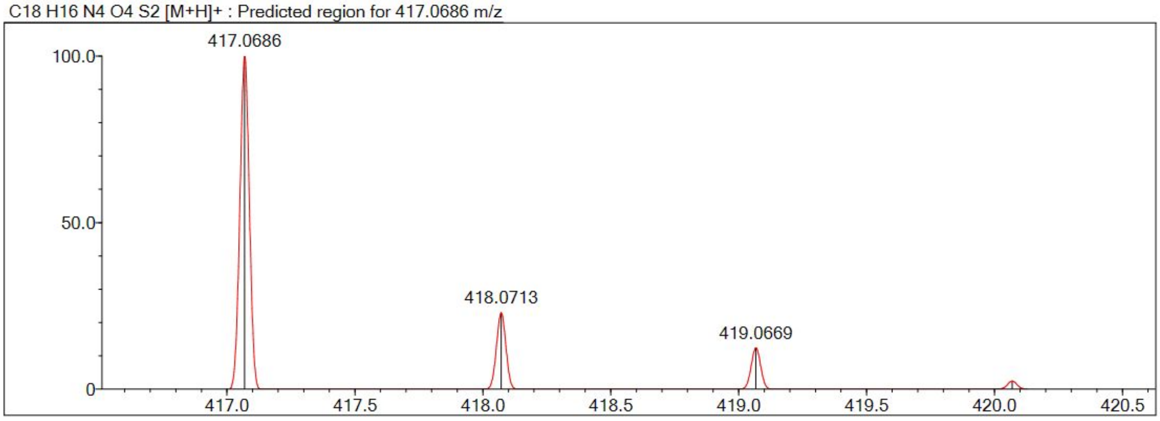

Rank	Score	Formula (M)	Ion	Meas. m/z	Pred. m/z	Df. (mDa)	Df. (ppm)	Iso	DBE
1	0.00	C18 H16 N4 O4 S2	[M+H]+	417.0676	417.0686	-1.0	-2.40	0.00	13.0

Figure S4. Mass spectrum of compound **2a**

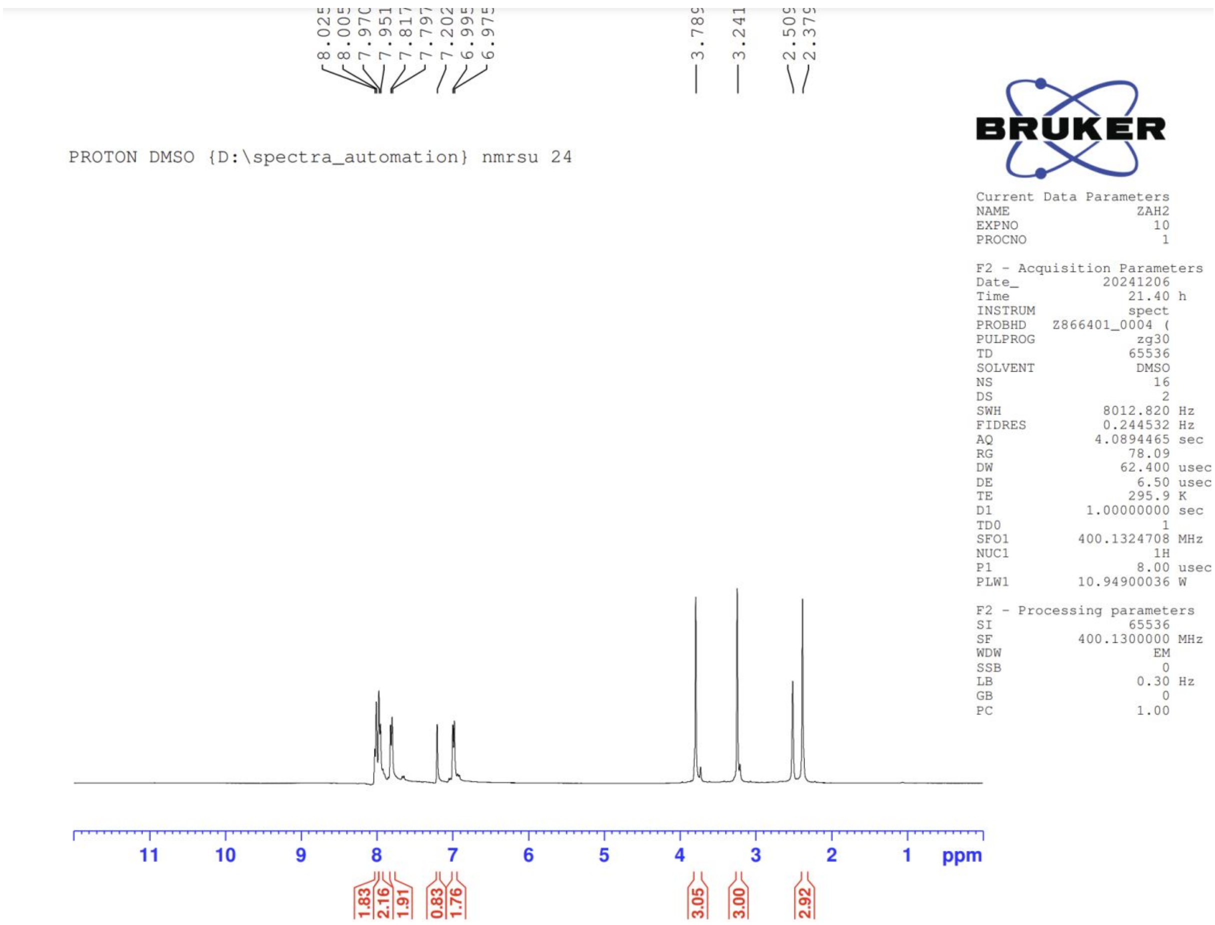

Figure S5. ^{1}H-NMR spectrum of compound **2b**

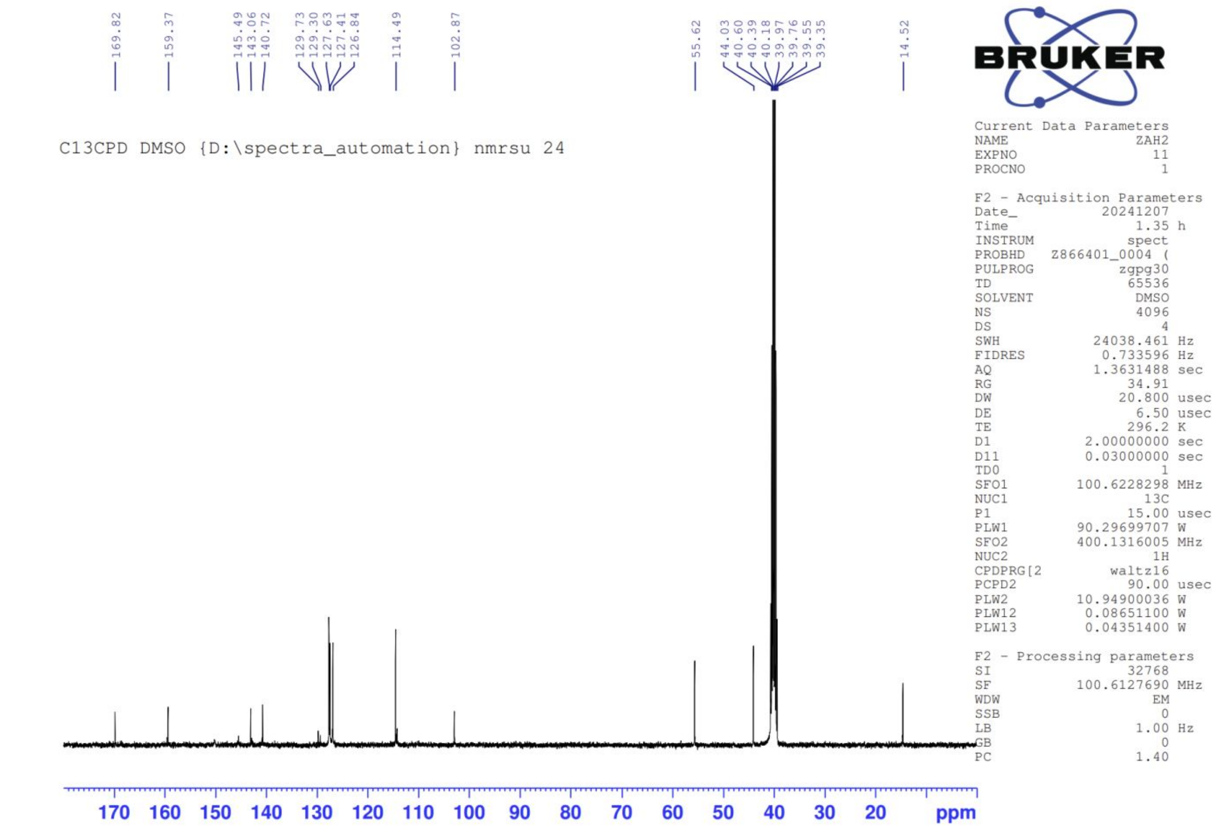

Figure S6. ^{13}C-NMR spectrum of compound **2b**

Data File: C:\LabSolutions\Data\Analiz\uac\ZAH-2_96.lcd

Elmt	Val.	Min	Max	Elmt	Val.	Min	Max	Elmt	Val.	Min	Max	Elmt	Val.	Min	Max	Use Adduct
H	1	5	40	O	2	3	4	S	2	2	2	Ru	2	0	0	H
C	4	5	30	F	1	0	0	Cl	1	0	0	Pd	2	0	0	
N	3	0	5	P	3	0	0	Br	1	0	0	I	3	0	0	

Error Margin (ppm):	5	DBE Range:	12.0 - 25.0	Electron Ions:	both
HC Ratio:	unlimited	Apply N Rule:	yes	Use MSn Info:	yes
Max Isotopes:	3	Isotope RI (%):	1.00	Isotope Res:	9000
MSn Iso RI (%):	10.00	MSn Logic Mode:	AND	Max Results:	200

Event#: 1 MS(E+) Ret. Time : 3.227 -> 3.227 Scan# : 485 -> 485

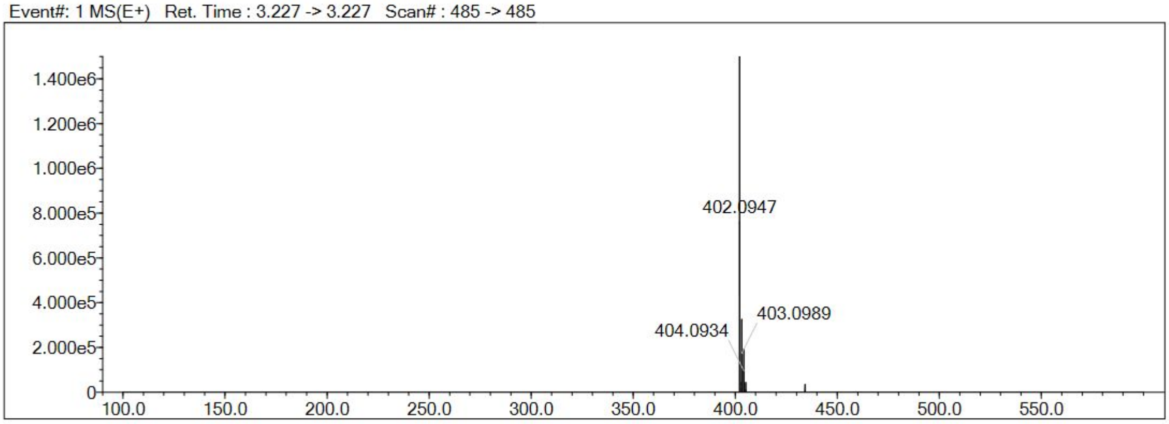

Measured region for 402.0947 m/z

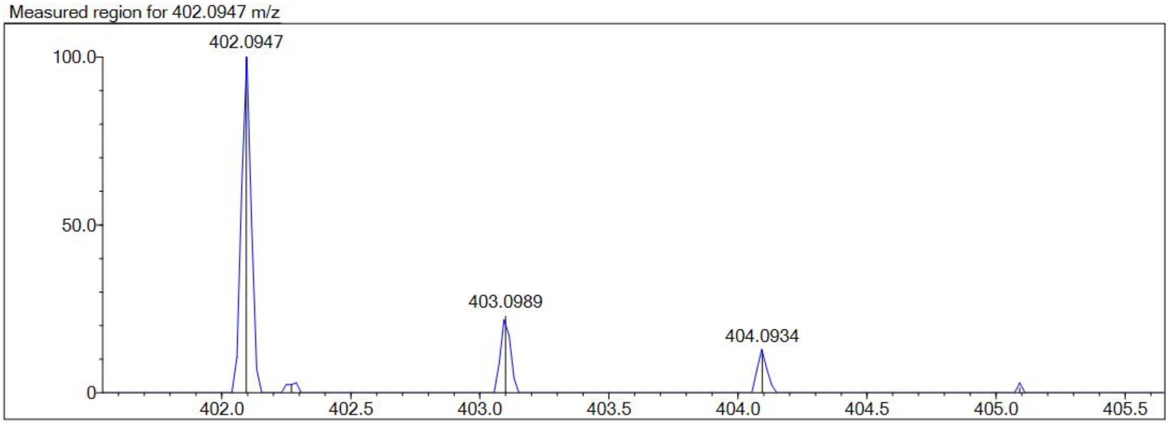

C19 H19 N3 O3 S2 [M+H]+ : Predicted region for 402.0941 m/z

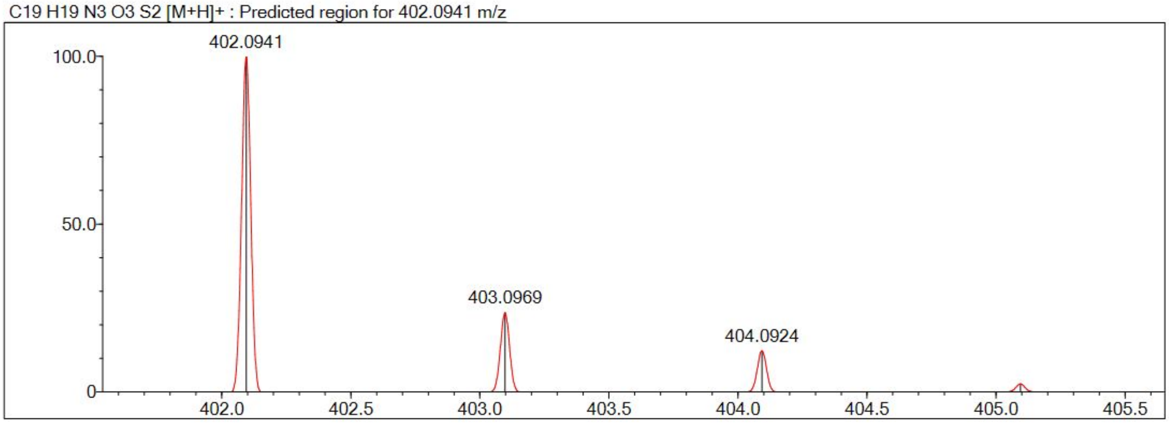

Rank	Score	Formula (M)	Ion	Meas. m/z	Pred. m/z	Df. (mDa)	Df. (ppm)	Iso	DBE
1	92.04	C19 H19 N3 O3 S2	[M+H]+	402.0947	402.0941	0.6	1.49	93.18	12.0

Figure S7. Mass spectrum of compound **2b**

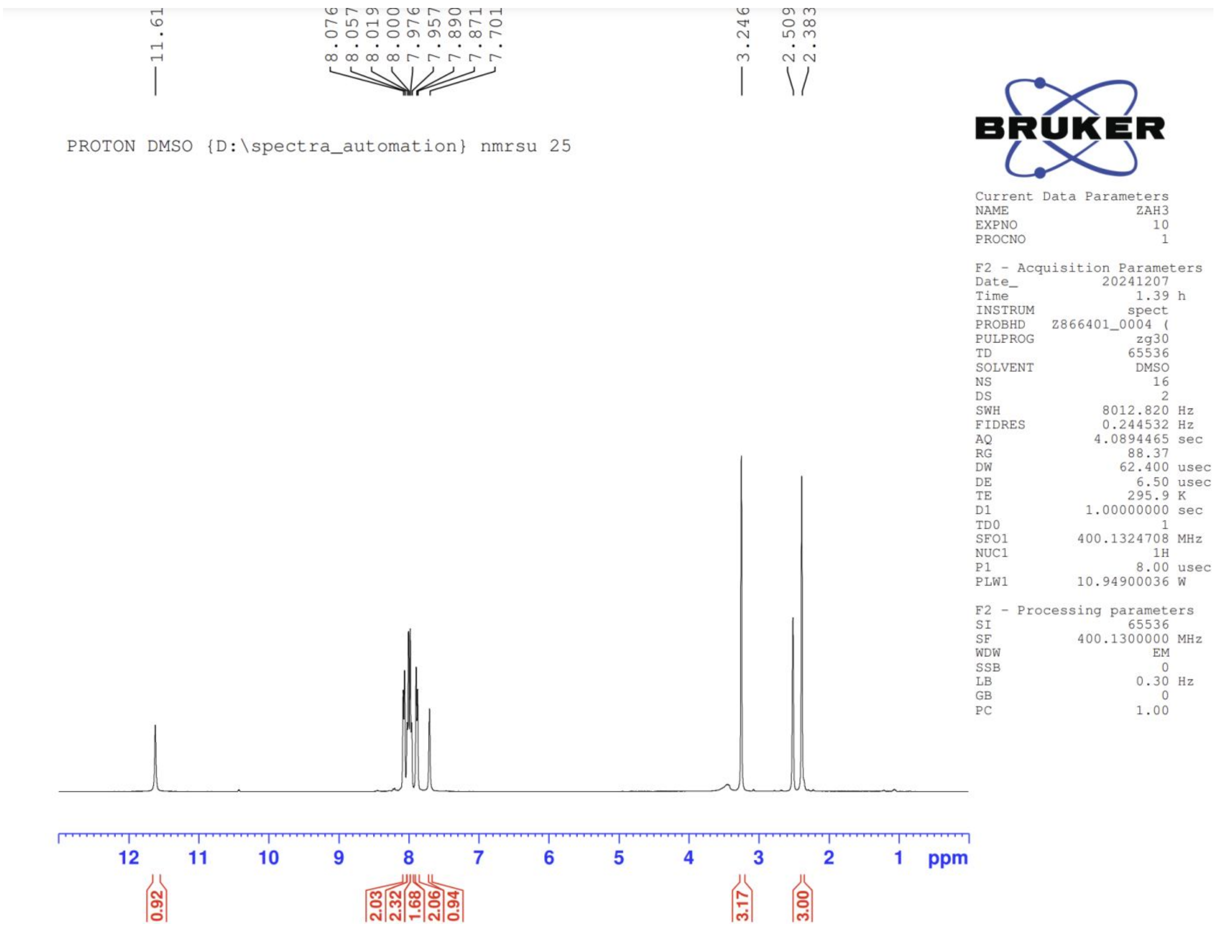

Figure S8. ^{1}H-NMR spectrum of compound **2c**

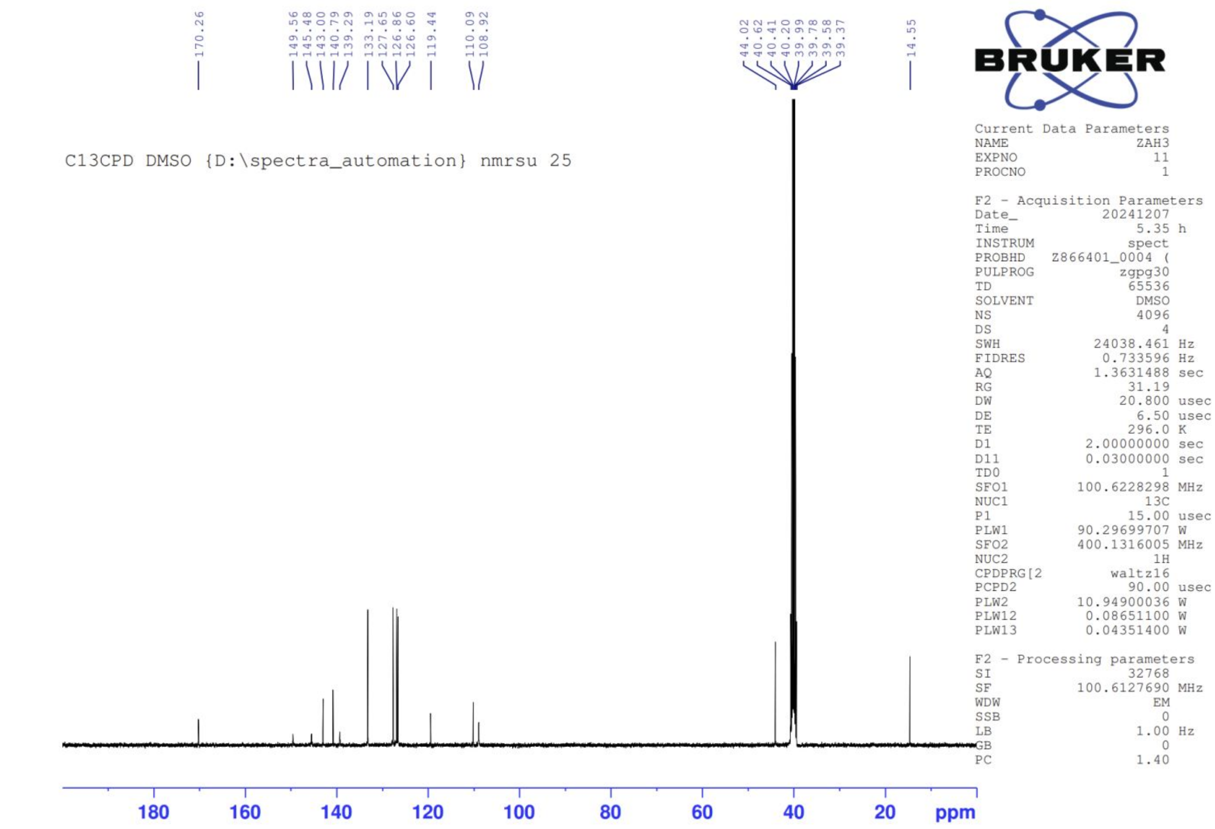

Figure S9. ^{13}C-NMR spectrum of compound **2c**

Formula Predictor Report - ZAH-3_97.lcd

Page 1 of 1

Data File: C:\LabSolutions\Data\Analiz\uac\ZAH-3_97.lcd

Elmt	Val.	Min	Max	Elmt	Val.	Min	Max	Elmt	Val.	Min	Max	Elmt	Val.	Min	Max	Use Adduct
H	1	5	40	O	2	0	4	S	2	2	2	Ru	2	0	0	H
C	4	5	30	F	1	0	0	Cl	1	0	0	Pd	2	0	0	
N	3	0	5	P	3	0	0	Br	1	0	0	I	3	0	0	

Error Margin (ppm):	5	DBE Range:	12.0 - 25.0	Electron Ions:	both
HC Ratio:	unlimited	Apply N Rule:	yes	Use MSn Info:	yes
Max Isotopes:	3	Isotope RI (%):	1.00	Isotope Res:	9000
MSn Iso RI (%):	10.00	MSn Logic Mode:	AND	Max Results:	200

Event#: 1 MS(E+) Ret. Time : 3.200 Scan# : 481

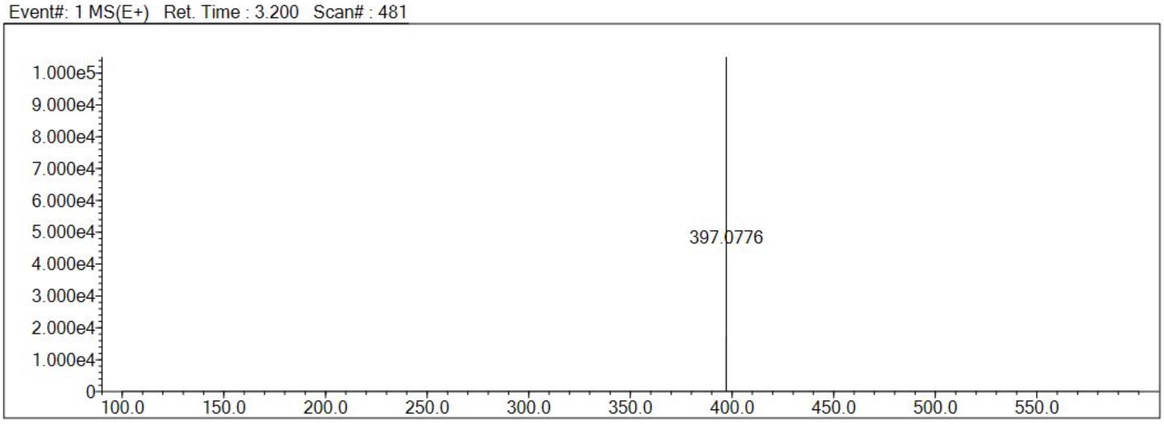

Measured region for 397.0776 m/z

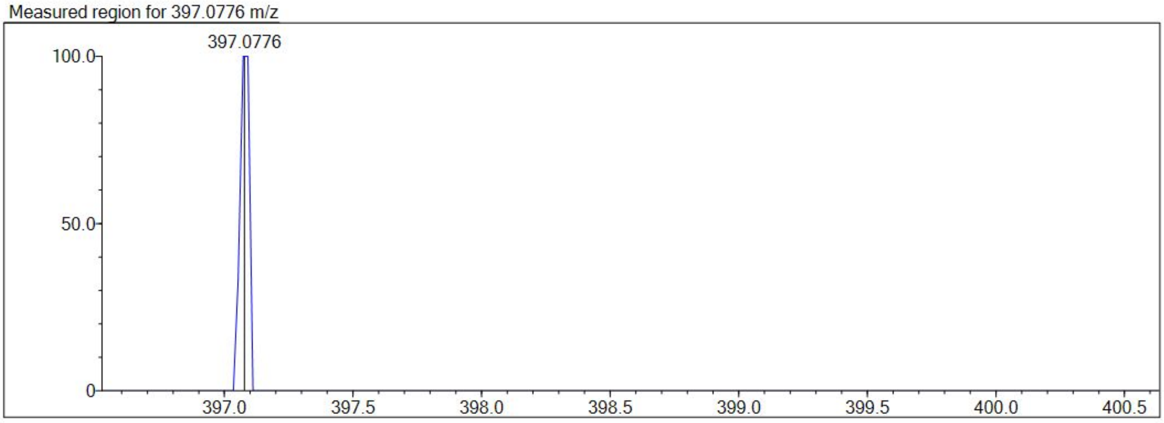

C19 H16 N4 O2 S2 [M+H]+ : Predicted region for 397.0787 m/z

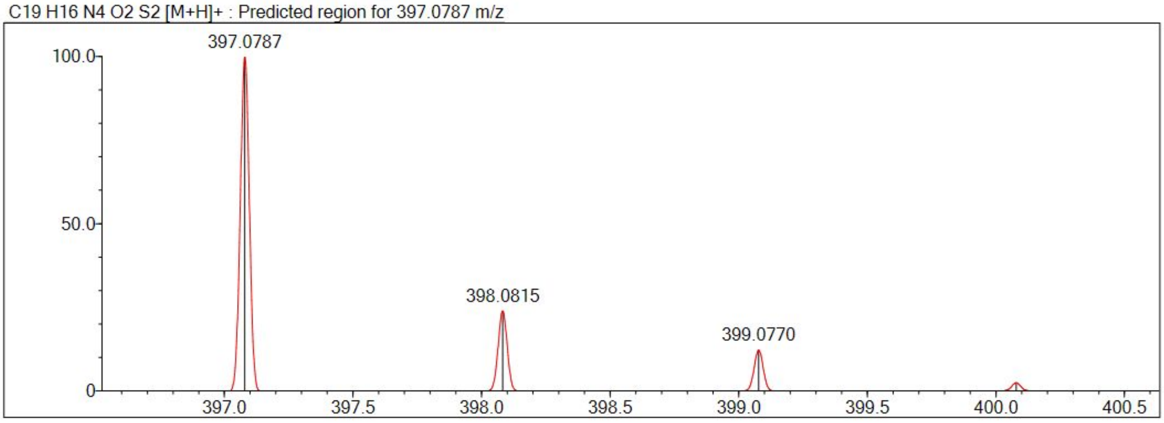

Rank	Score	Formula (M)	Ion	Meas. m/z	Pred. m/z	Df. (mDa)	Df. (ppm)	Iso	DBE
1	0.00	C19 H16 N4 O2 S2	[M+H]+	397.0776	397.0787	-1.1	-2.77	0.00	14.0

Figure S10. Mass spectrum of compound **2c**

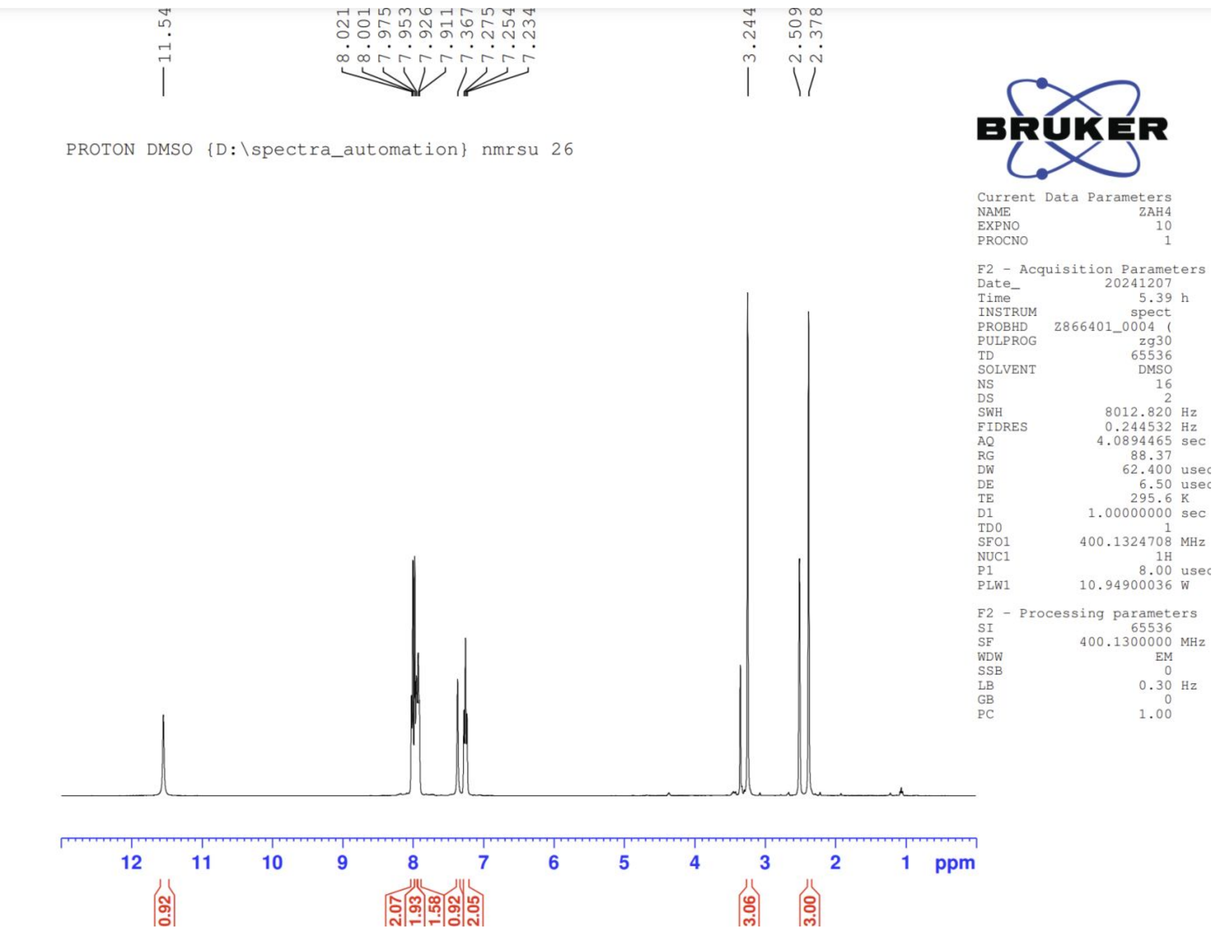

Figure S11. ^{1}H-NMR spectrum of compound **2d**

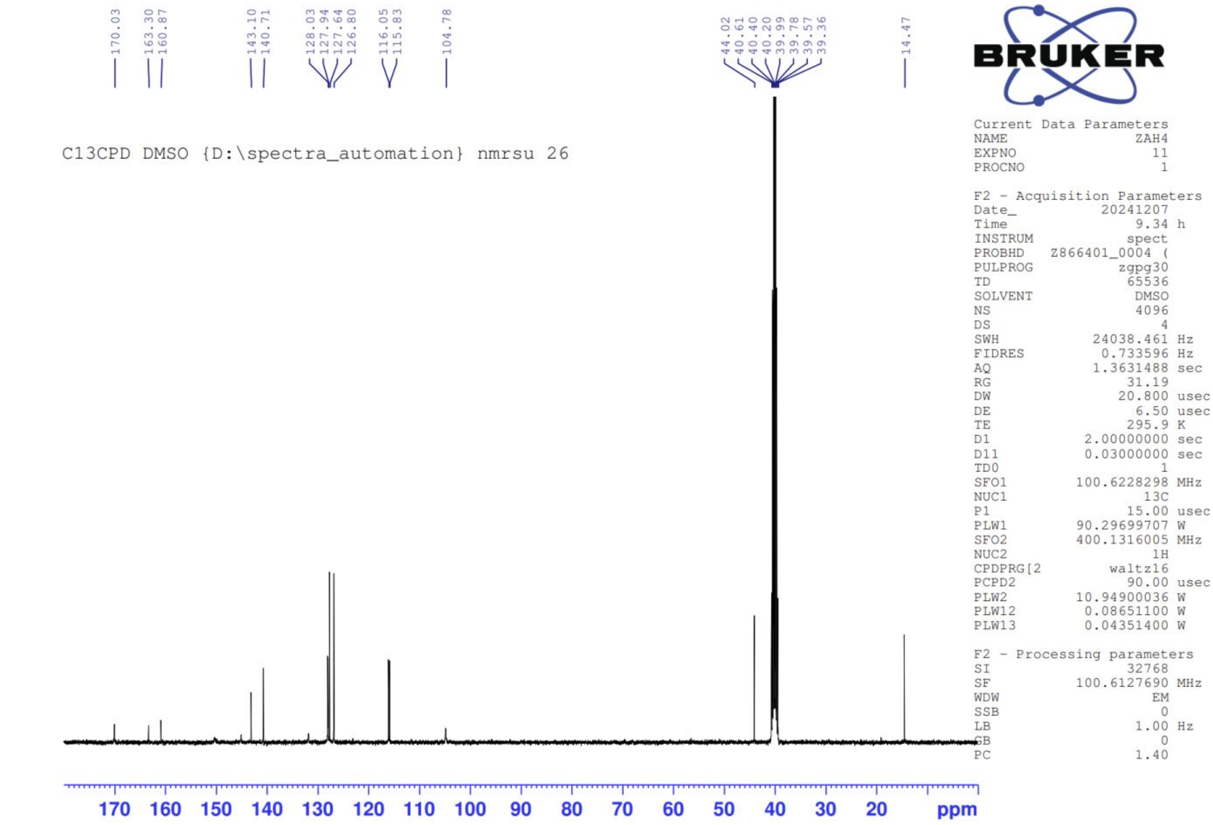

Figure S12. ^{13}C-NMR spectrum of compound **2d**

Data File: C:\LabSolutions\Data\Analiz\uac\ZAH-4_98.lcd

Elmt	Val.	Min	Max	Elmt	Val.	Min	Max	Elmt	Val.	Min	Max	Elmt	Val.	Min	Max	Use Adduct
H	1	5	40	O	2	0	4	S	2	2	2	Ru	2	0	0	H
C	4	5	30	F	1	1	1	Cl	1	0	0	Pd	2	0	0	
N	3	0	5	P	3	0	0	Br	1	0	0	I	3	0	0	

Error Margin (ppm): 5
HC Ratio: unlimited
Max Isotopes: 3
MSn Iso RI (%): 10.00

DBE Range: 12.0 - 25.0
Apply N Rule: yes
Isotope RI (%): 1.00
MSn Logic Mode: AND

Electron Ions: both
Use MSn Info: yes
Isotope Res: 9000
Max Results: 200

Event#: 1 MS(E+) Ret. Time : 3.373 -> 4.093 Scan# : 507 -> 615

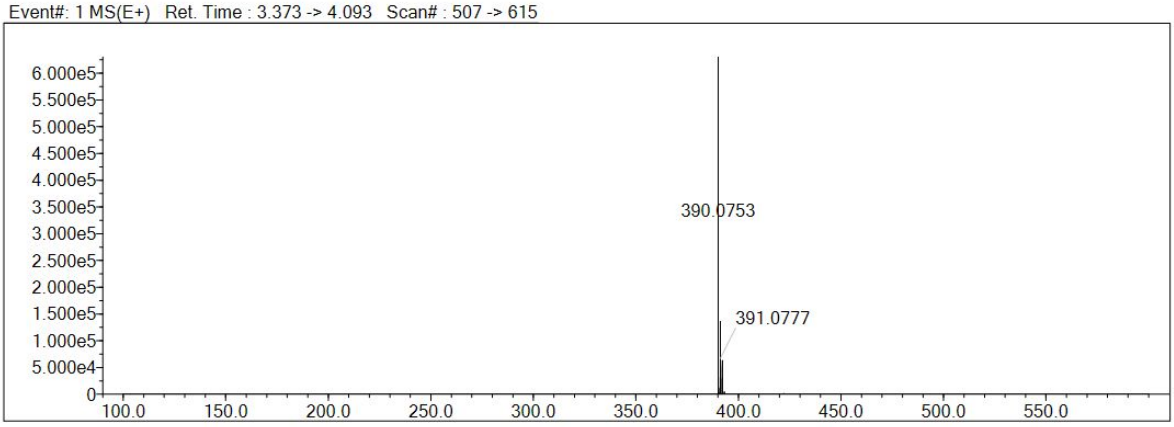

Measured region for 390.0753 m/z

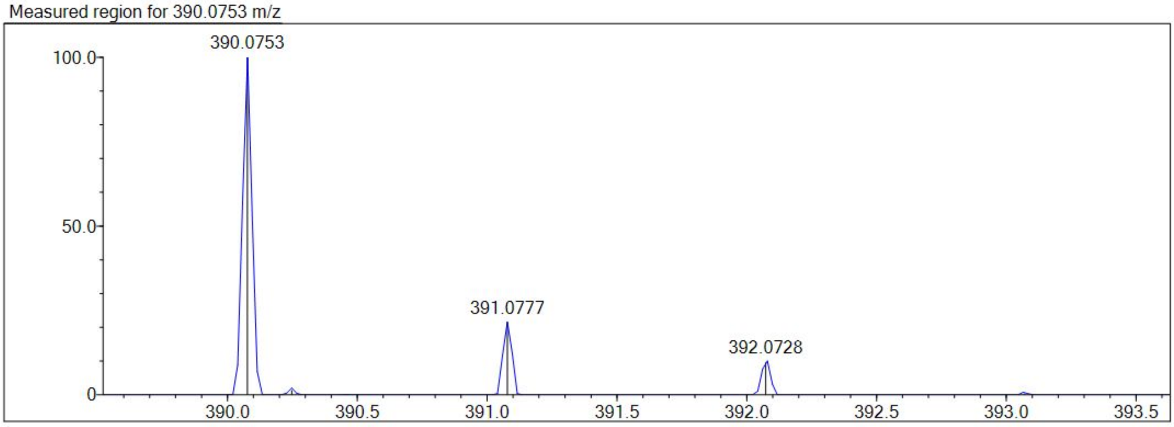

C18 H16 N3 O2 F S2 [M+H]+ : Predicted region for 390.0741 m/z

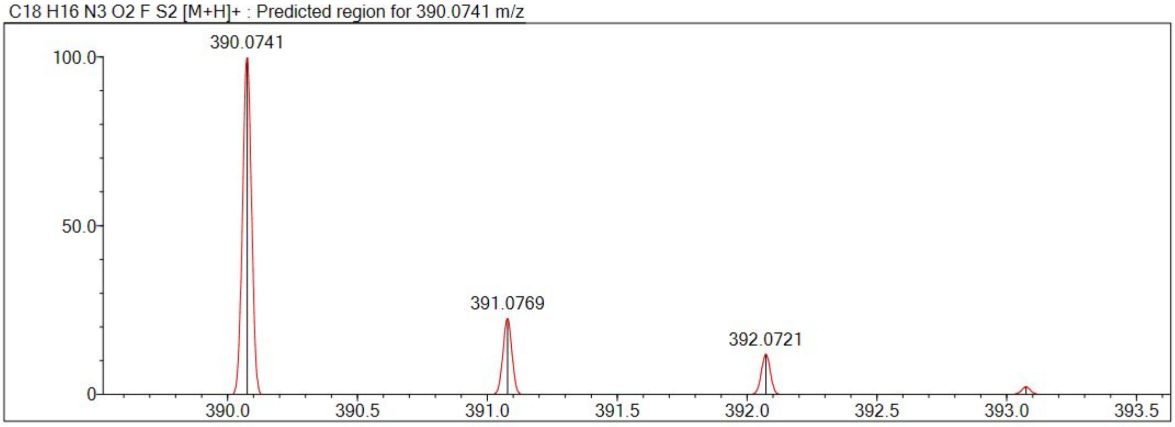

Rank	Score	Formula (M)	Ion	Meas. m/z	Pred. m/z	Df. (mDa)	Df. (ppm)	Iso	DBE
1	73.25	C18 H16 N3 O2 F S2	[M+H]+	390.0753	390.0741	1.2	3.08	77.26	12.0

Figure S13. Mass spectrum of compound **2d**

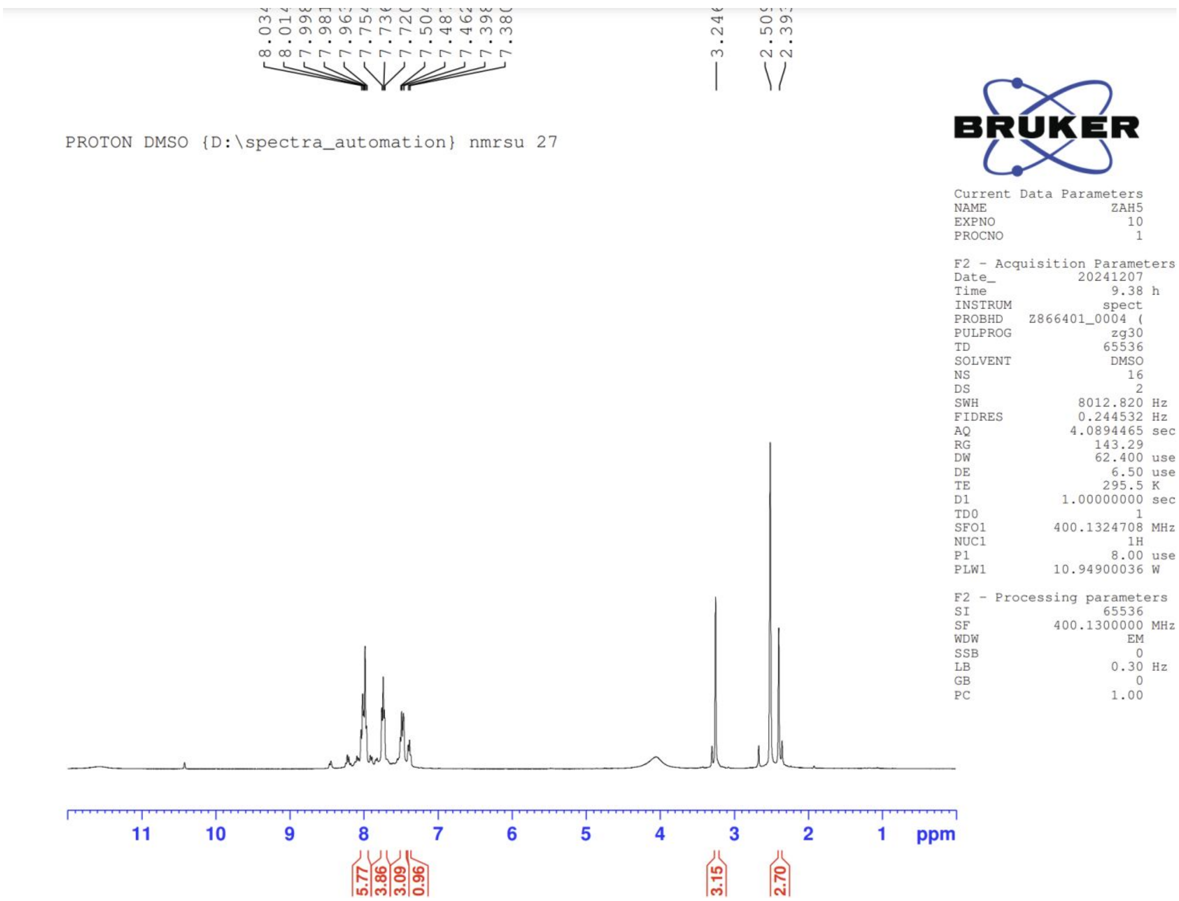

Figure S14. ^{1}H-NMR spectrum of compound **2e**

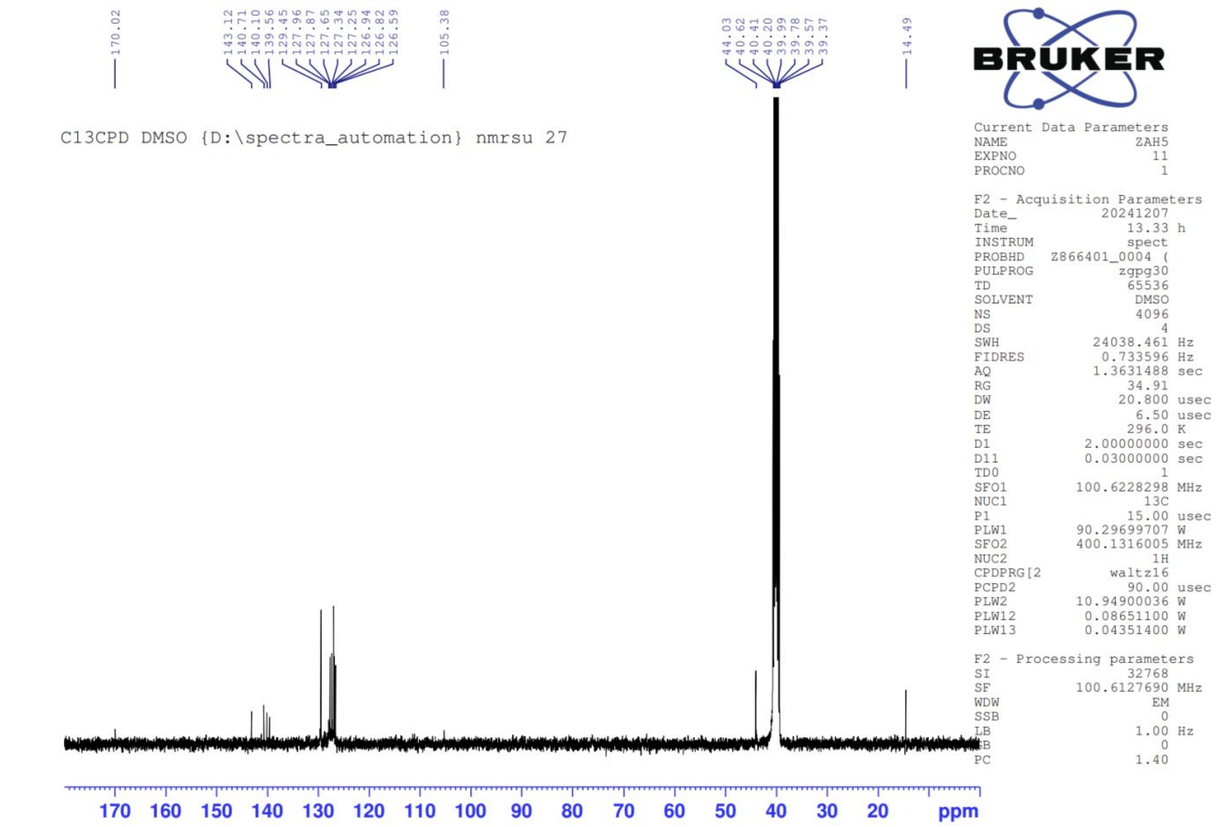

Figure S15. ^{13}C-NMR spectrum of compound **2e**

Data File: C:\LabSolutions\Data\Analiz\uac\ZAH-5_99.lcd

Elmt	Val.	Min	Max	Elmt	Val.	Min	Max	Elmt	Val.	Min	Max	Elmt	Val.	Min	Max	Use Adduct
H	1	5	40	O	2	0	4	S	2	2	2	Ru	2	0	0	H
C	4	5	30	F	1	0	0	Cl	1	0	0	Pd	2	0	0	
N	3	0	5	P	3	0	0	Br	1	0	0	I	3	0	0	

Error Margin (ppm):	5	DBE Range:	12.0 - 25.0	Electron Ions:	both
HC Ratio:	unlimited	Apply N Rule:	yes	Use MSn Info:	yes
Max Isotopes:	3	Isotope RI (%):	1.00	Isotope Res:	9000
MSn Iso RI (%):	10.00	MSn Logic Mode:	AND	Max Results:	200

Event#: 1 MS(E+) Ret. Time : 6.400 -> 7.280 Scan# : 961 -> 1093

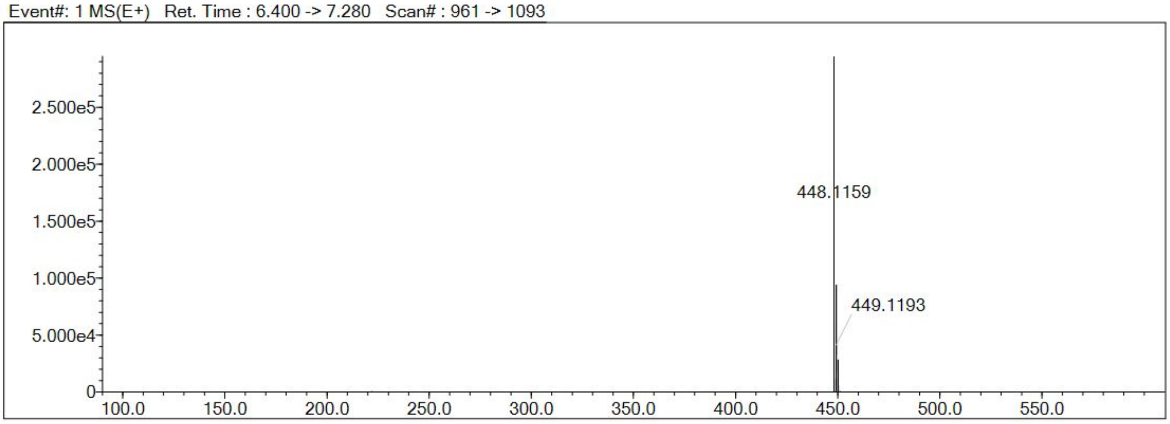

Measured region for 448.1159 m/z

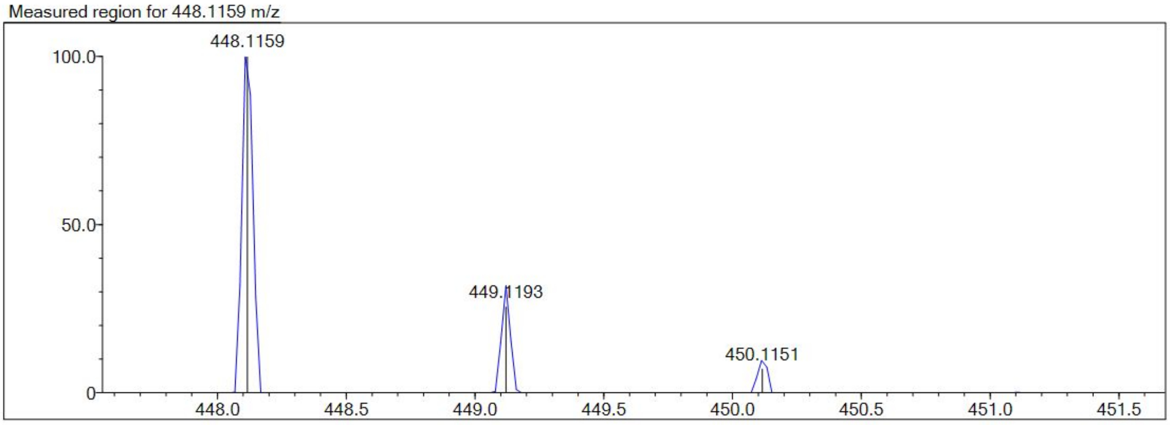

C24 H21 N3 O2 S2 [M+H]+ : Predicted region for 448.1148 m/z

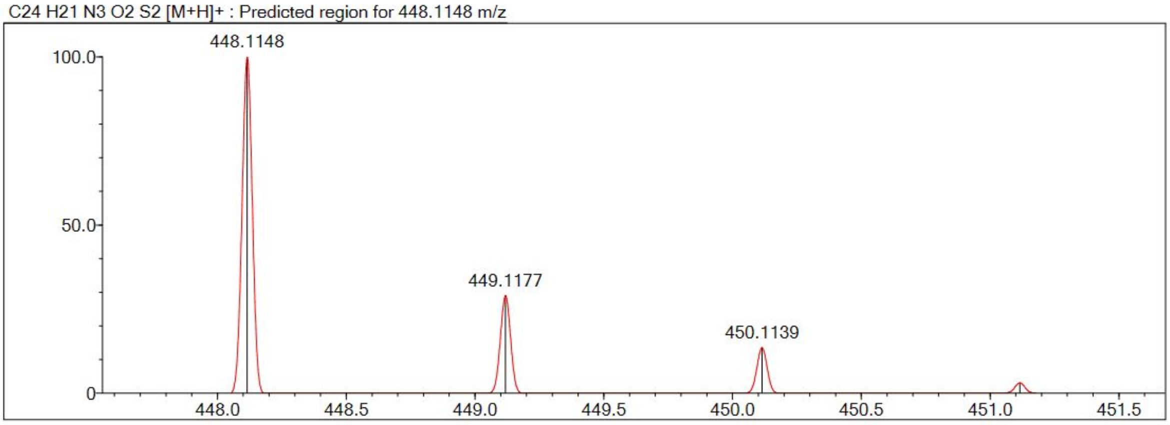

Rank	Score	Formula (M)	Ion	Meas. m/z	Pred. m/z	Df. (mDa)	Df. (ppm)	Iso	DBE
1	66.03	C24 H21 N3 O2 S2	[M+H]+	448.1159	448.1148	1.1	2.45	68.52	16.0

Figure S16. Mass spectrum of compound **2e**

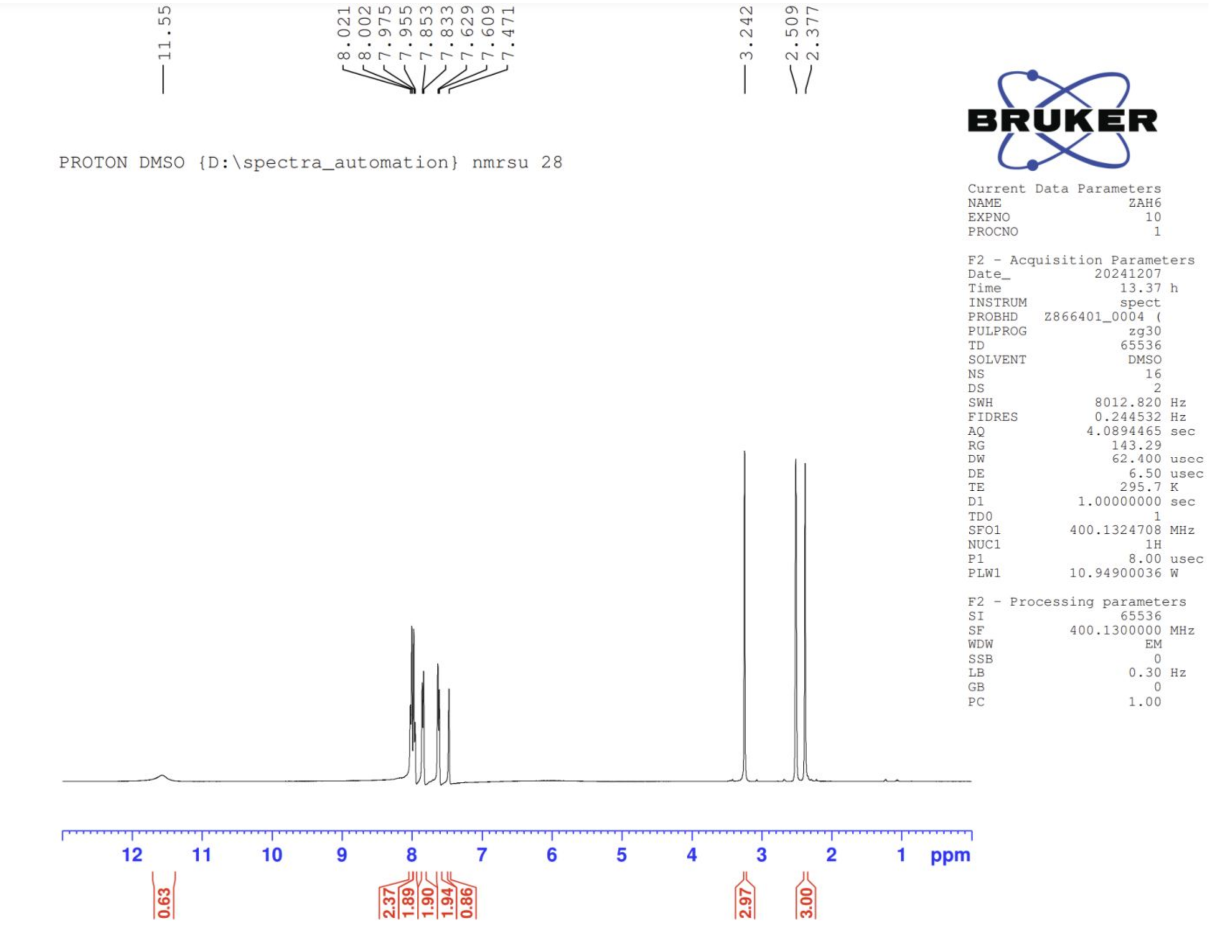

Figure S17. ^{1}H-NMR spectrum of compound **2f**

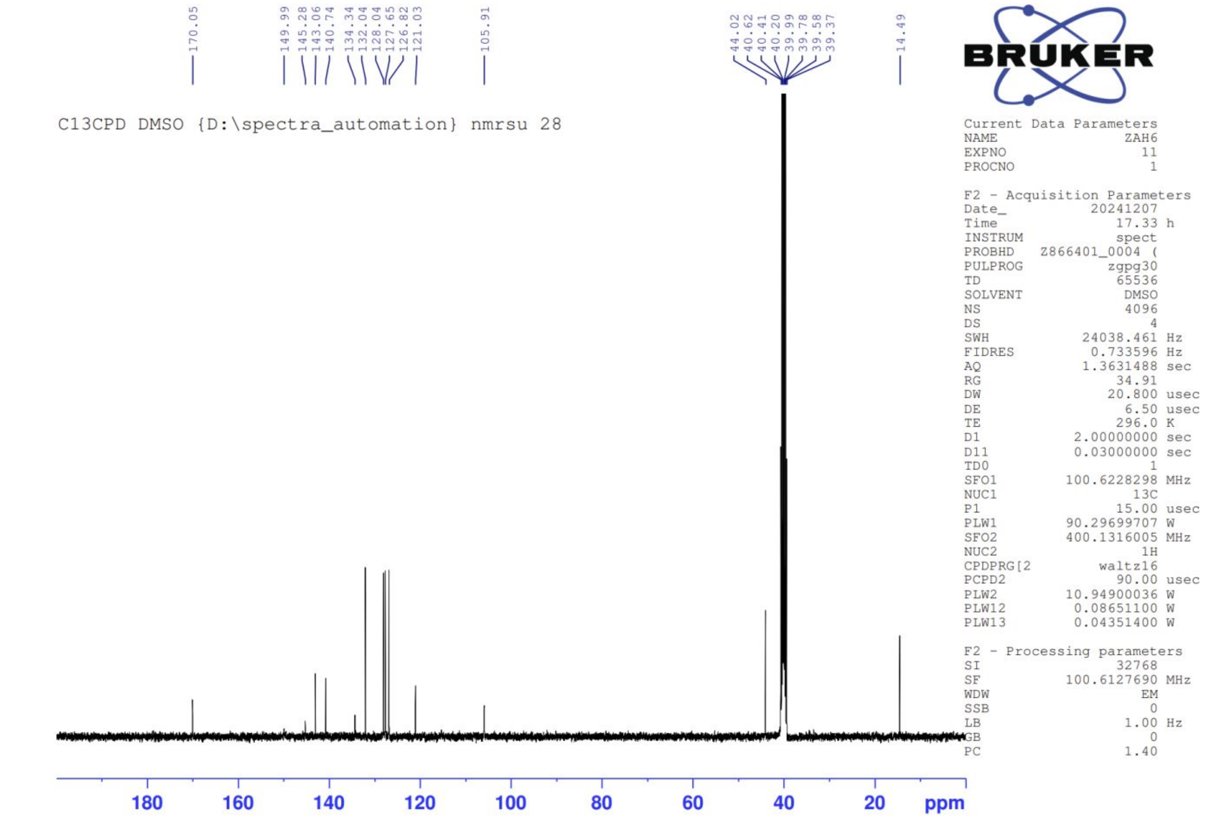

Figure S18. ^{13}C-NMR spectrum of compound **2f**

Data File: C:\LabSolutions\Data\Analiz\uac\ZAH-6_100.lcd

Elmt	Val.	Min	Max	Elmt	Val.	Min	Max	Elmt	Val.	Min	Max	Elmt	Val.	Min	Max	Use Adduct
H	1	5	40	O	2	0	4	S	2	2	2	Ru	2	0	0	H
C	4	5	30	F	1	0	0	Cl	1	0	0	Pd	2	0	0	
N	3	0	5	P	3	0	0	Br	1	1	1	I	3	0	0	

Error Margin (ppm): 5
HC Ratio: unlimited
Max Isotopes: 3
MSn Iso RI (%): 10.00

DBE Range: 12.0 - 25.0
Apply N Rule: yes
Isotope RI (%): 1.00
MSn Logic Mode: AND

Electron Ions: both
Use MSn Info: yes
Isotope Res: 9000
Max Results: 200

Event#: 1 MS(E+) Ret. Time : 5.053 Scan# : 759

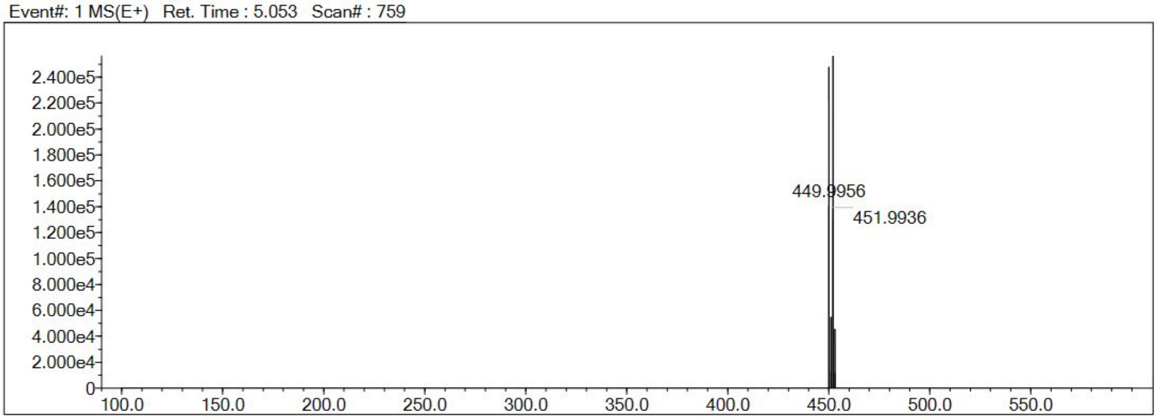

Measured region for 449.9956 m/z

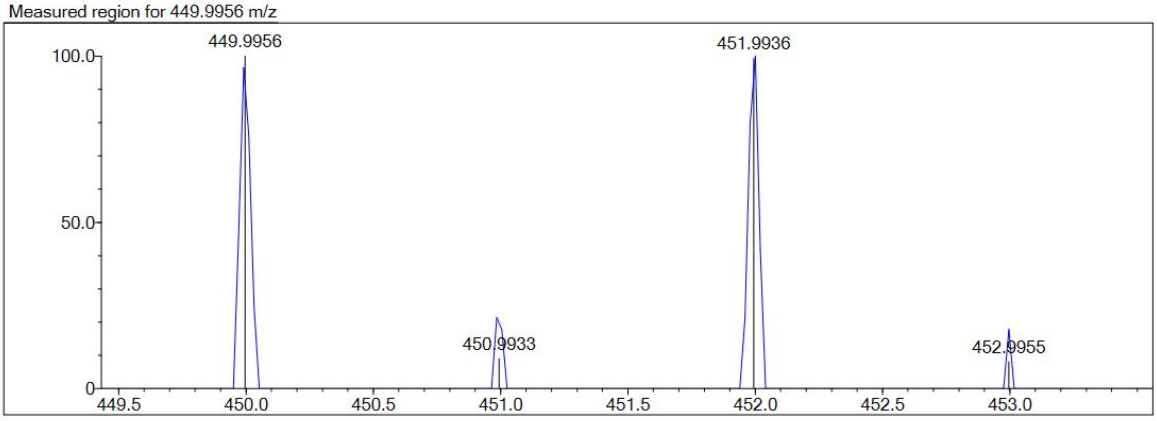

C18 H16 N3 O2 S2 Br [M+H]+ : Predicted region for 449.9940 m/z

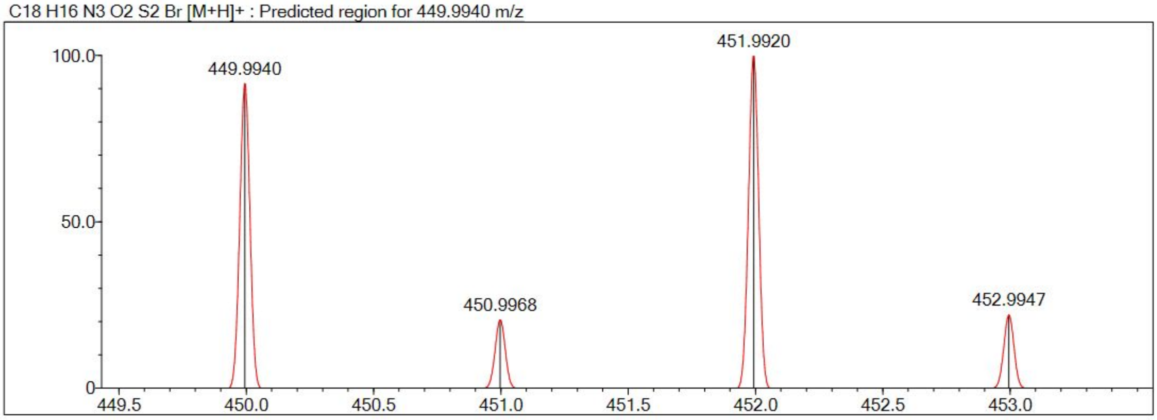

Rank	Score	Formula (M)	Ion	Meas. m/z	Pred. m/z	Df. (mDa)	Df. (ppm)	Iso	DBE
1	64.79	C18 H16 N3 O2 S2 Br	[M+H]+	449.9956	449.9940	1.6	3.56	69.22	12.0

Figure S19. Mass spectrum of compound **2f**

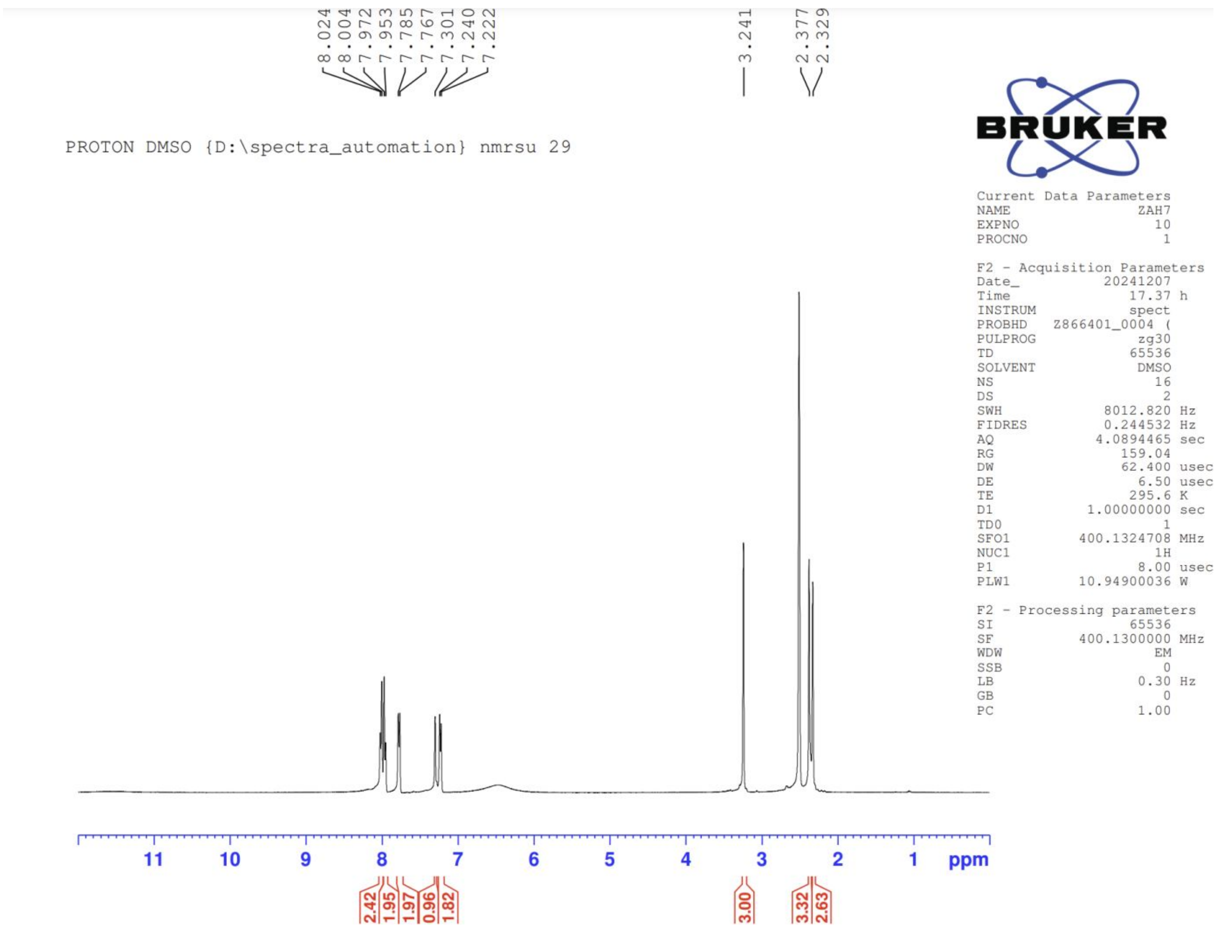

Figure S20. ^{1}H-NMR spectrum of compound **2g**

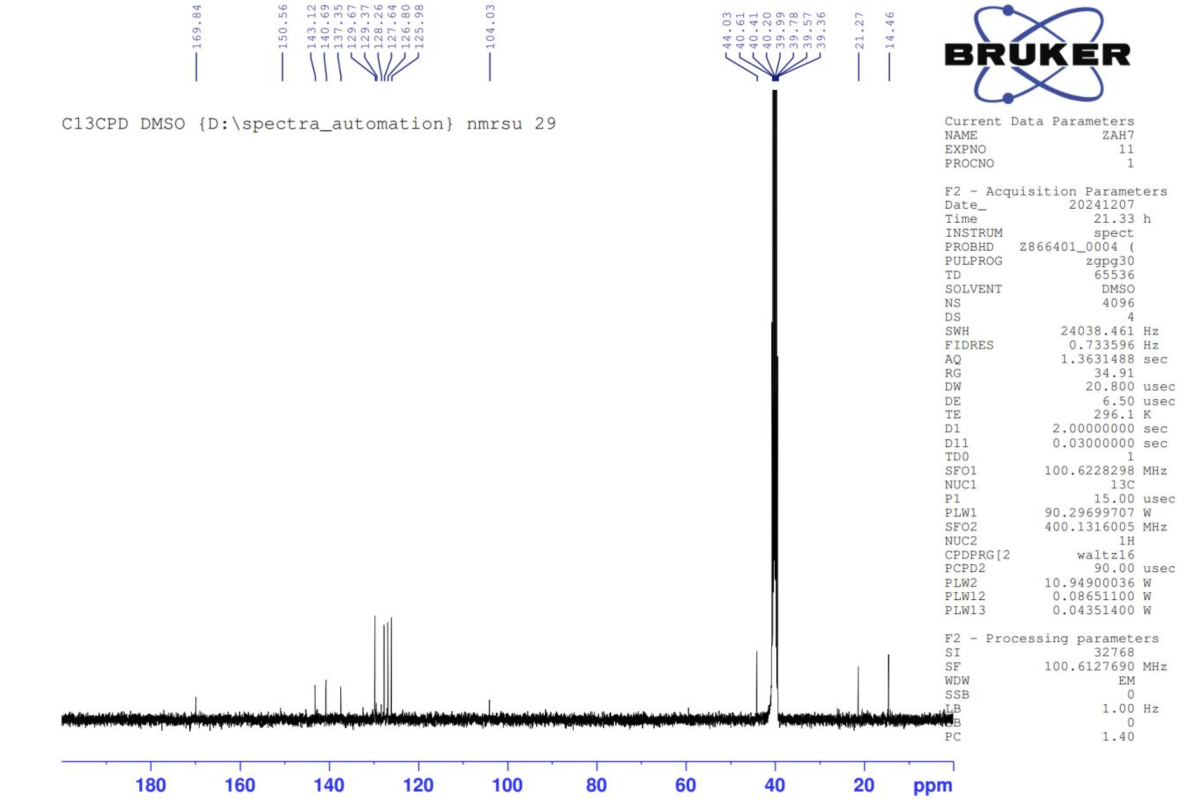

Figure S21. ^{13}C-NMR spectrum of compound **2g**

Data File: C:\LabSolutions\Data\Analiz\uac\ZAH-7_101.lcd

Elmt	Val.	Min	Max	Elmt	Val.	Min	Max	Elmt	Val.	Min	Max	Elmt	Val.	Min	Max	Use Adduct
H	1	5	40	O	2	0	4	S	2	2	2	Ru	2	0	0	H
C	4	5	30	F	1	0	0	Cl	1	0	0	Pd	2	0	0	
N	3	0	5	P	3	0	0	Br	1	0	0	I	3	0	0	

Error Margin (ppm):	5	DBE Range:	12.0 - 25.0	Electron Ions:	both
HC Ratio:	unlimited	Apply N Rule:	yes	Use MSn Info:	yes
Max Isotopes:	3	Isotope RI (%):	1.00	Isotope Res:	9000
MSn Iso RI (%):	10.00	MSn Logic Mode:	AND	Max Results:	200

Event#: 1 MS(E+) Ret. Time : 4.013 -> 4.547 Scan# : 603 -> 683

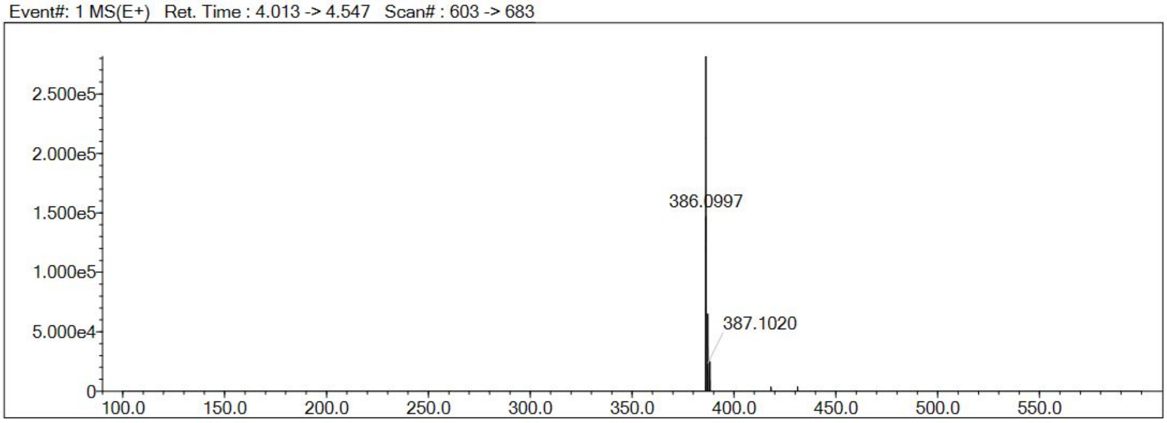

Measured region for 386.0997 m/z

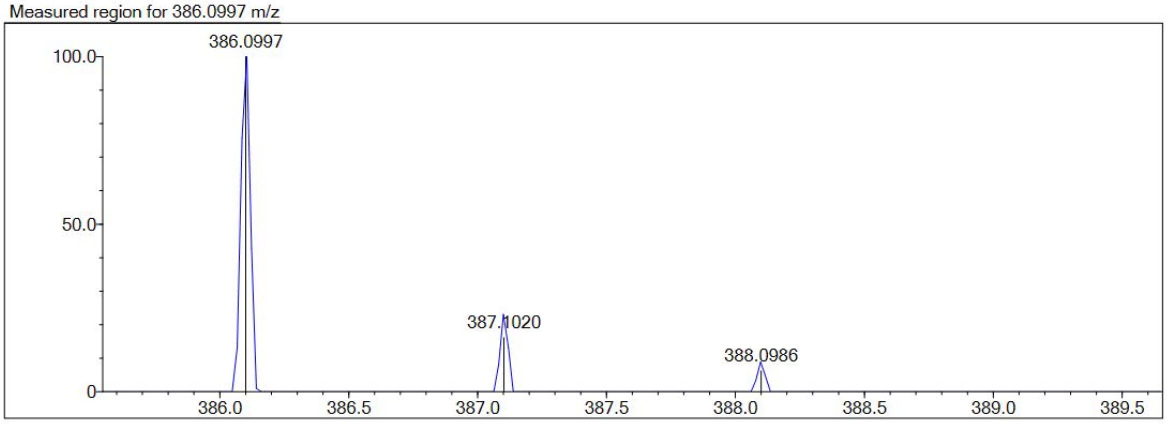

C19 H19 N3 O2 S2 [M+H]+ : Predicted region for 386.0991 m/z

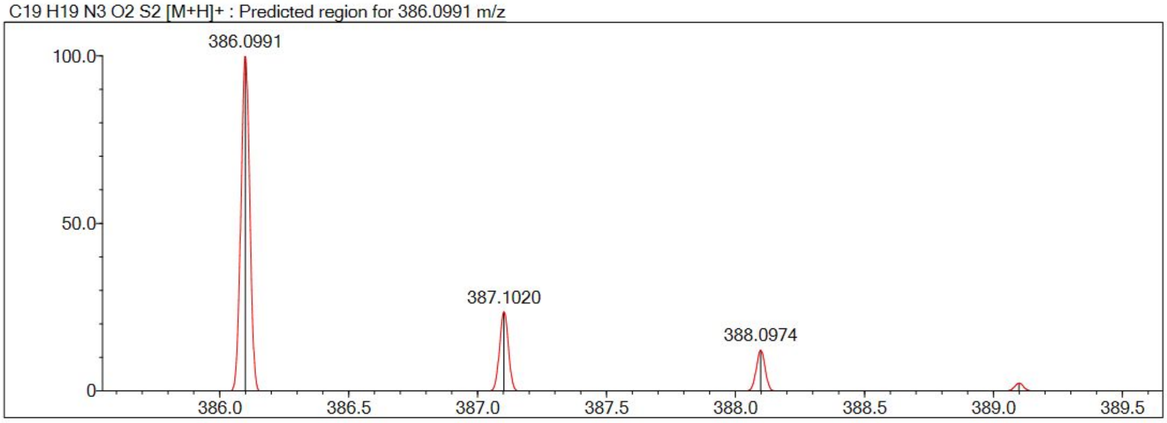

Rank	Score	Formula (M)	Ion	Meas. m/z	Pred. m/z	Df. (mDa)	Df. (ppm)	Iso	DBE
1	77.34	C19 H19 N3 O2 S2	[M+H]+	386.0997	386.0991	0.6	1.55	78.42	12.0

Figure S22. Mass spectrum of compound **2g**

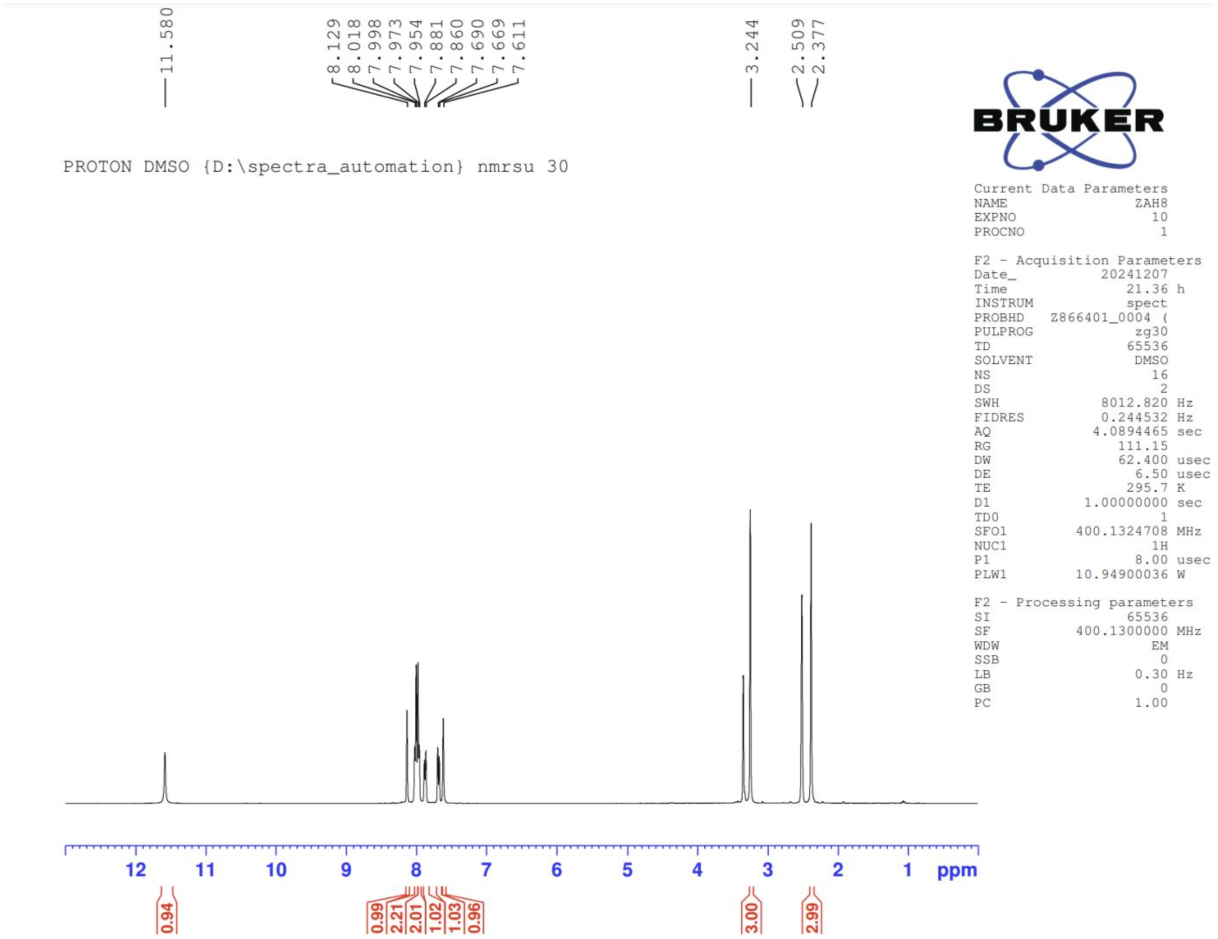

Figure S23. ^{1}H-NMR spectrum of compound **2h**

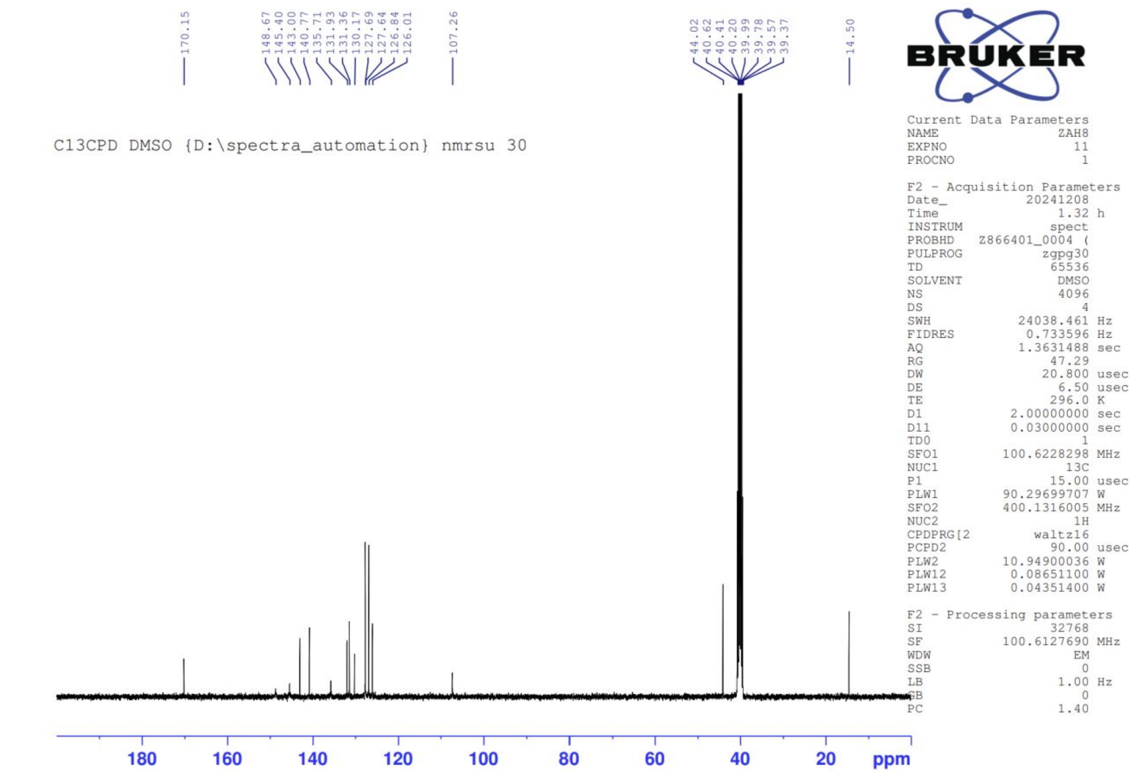

Figure S24. ^{13}C-NMR spectrum of compound **2h**

Data File: C:\LabSolutions\Data\Analiz\uac\ZAH-8_102.lcd

Elmt	Val.	Min	Max	Elmt	Val.	Min	Max	Elmt	Val.	Min	Max	Elmt	Val.	Min	Max	Use Adduct
H	1	5	40	O	2	0	4	S	2	2	2	Ru	2	0	0	H
C	4	5	30	F	1	0	0	Cl	1	2	2	Pd	2	0	0	
N	3	0	5	P	3	0	0	Br	1	0	0	I	3	0	0	

Error Margin (ppm):	5	DBE Range:	12.0 - 25.0	Electron Ions:	both
HC Ratio:	unlimited	Apply N Rule:	yes	Use MSn Info:	yes
Max Isotopes:	3	Isotope RI (%):	1.00	Isotope Res:	9000
MSn Iso RI (%):	10.00	MSn Logic Mode:	AND	Max Results:	200

Event#: 1 MS(E+) Ret. Time : 6.493 -> 7.160 Scan# : 975 -> 1075

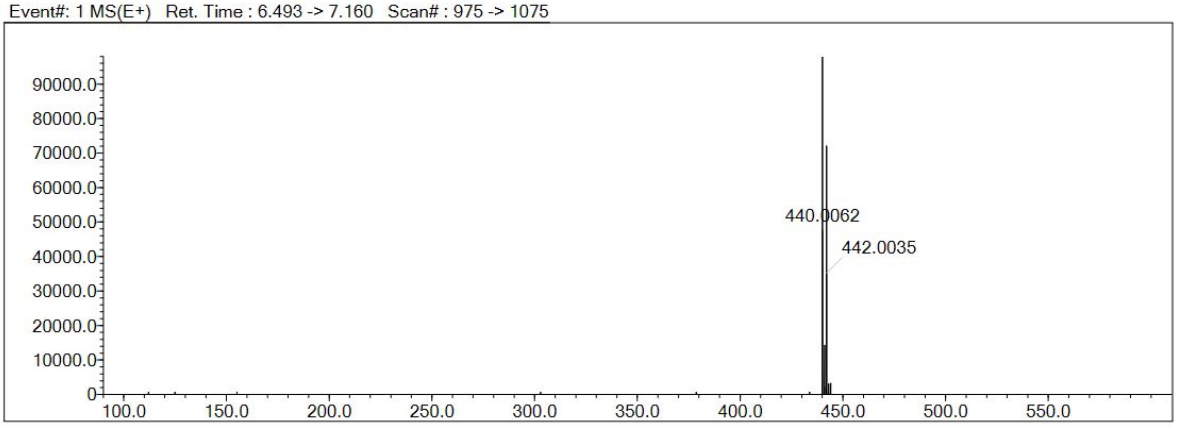

Measured region for 440.0062 m/z

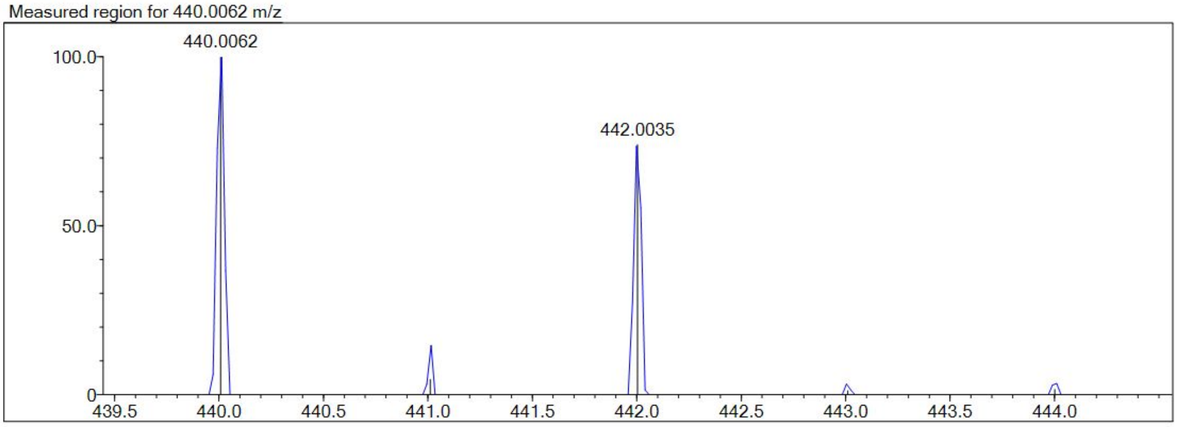

C18 H15 N3 O2 S2 Cl2 [M+H]+ : Predicted region for 440.0056 m/z

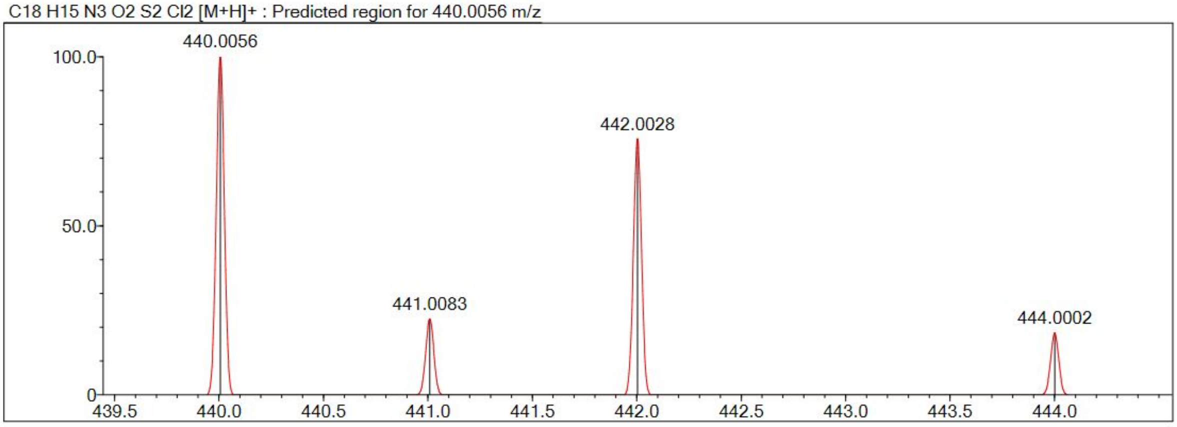

Rank	Score	Formula (M)	Ion	Meas. m/z	Pred. m/z	Df. (mDa)	Df. (ppm)	Iso	DBE
1	48.86	C18 H15 N3 O2 S2 Cl2	[M+H]+	440.0062	440.0056	0.6	1.36	49.31	12.0

Figure S25. Mass spectrum of compound **2h**

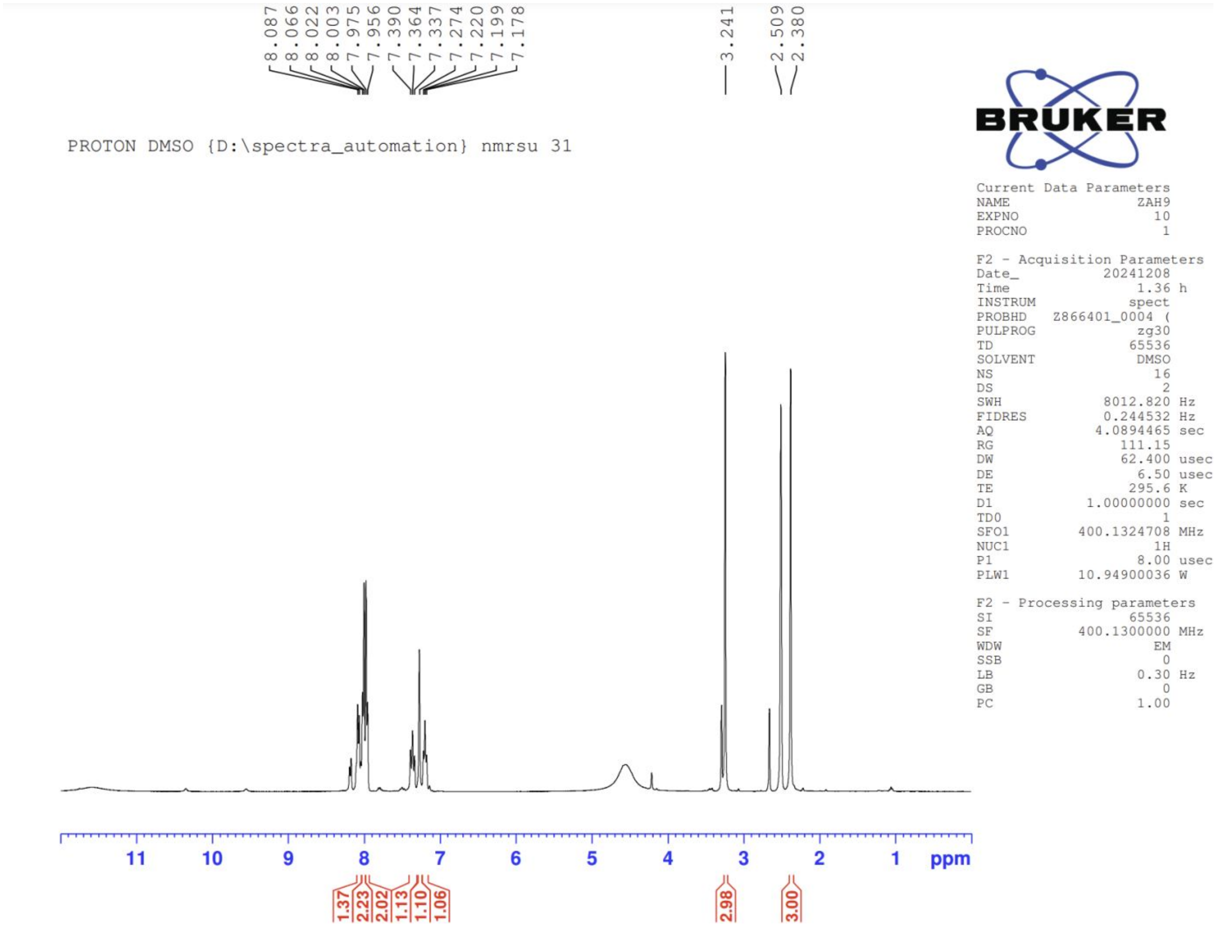

Figure S26. ^{1}H-NMR spectrum of compound **2i**

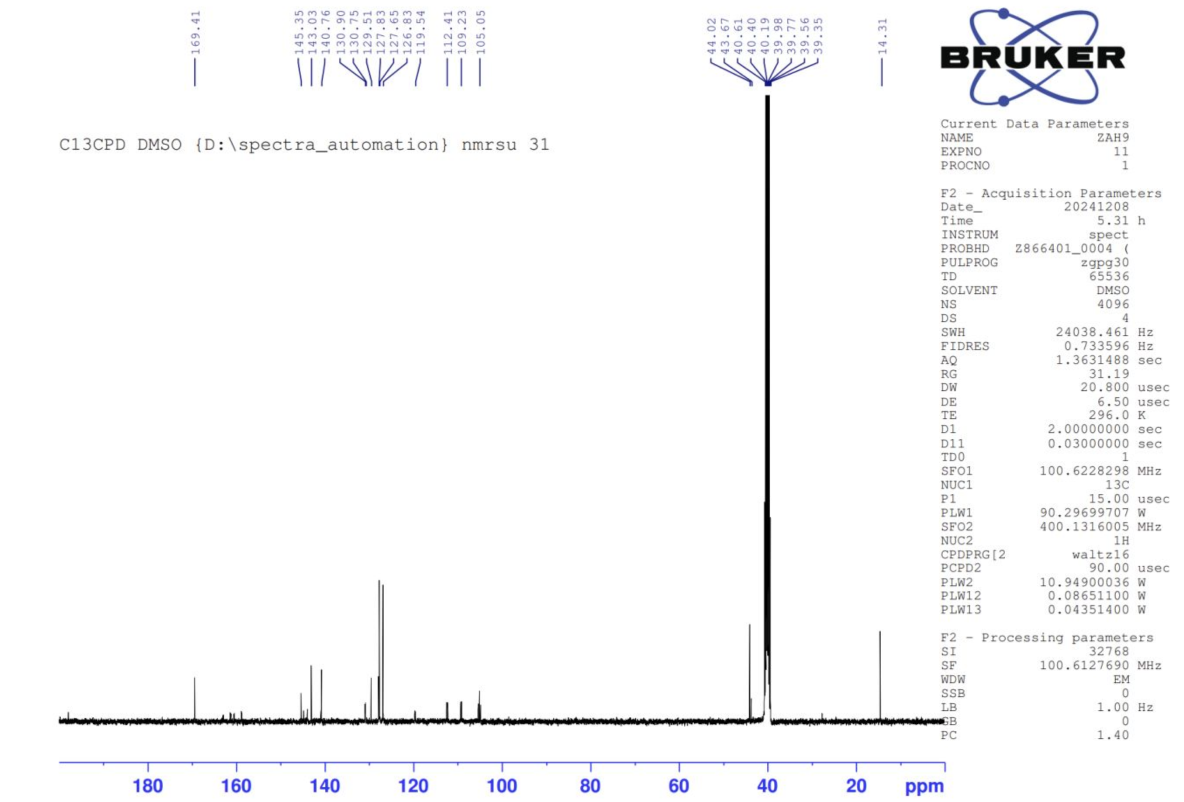

Figure S27. ^{13}C-NMR spectrum of compound **2i**

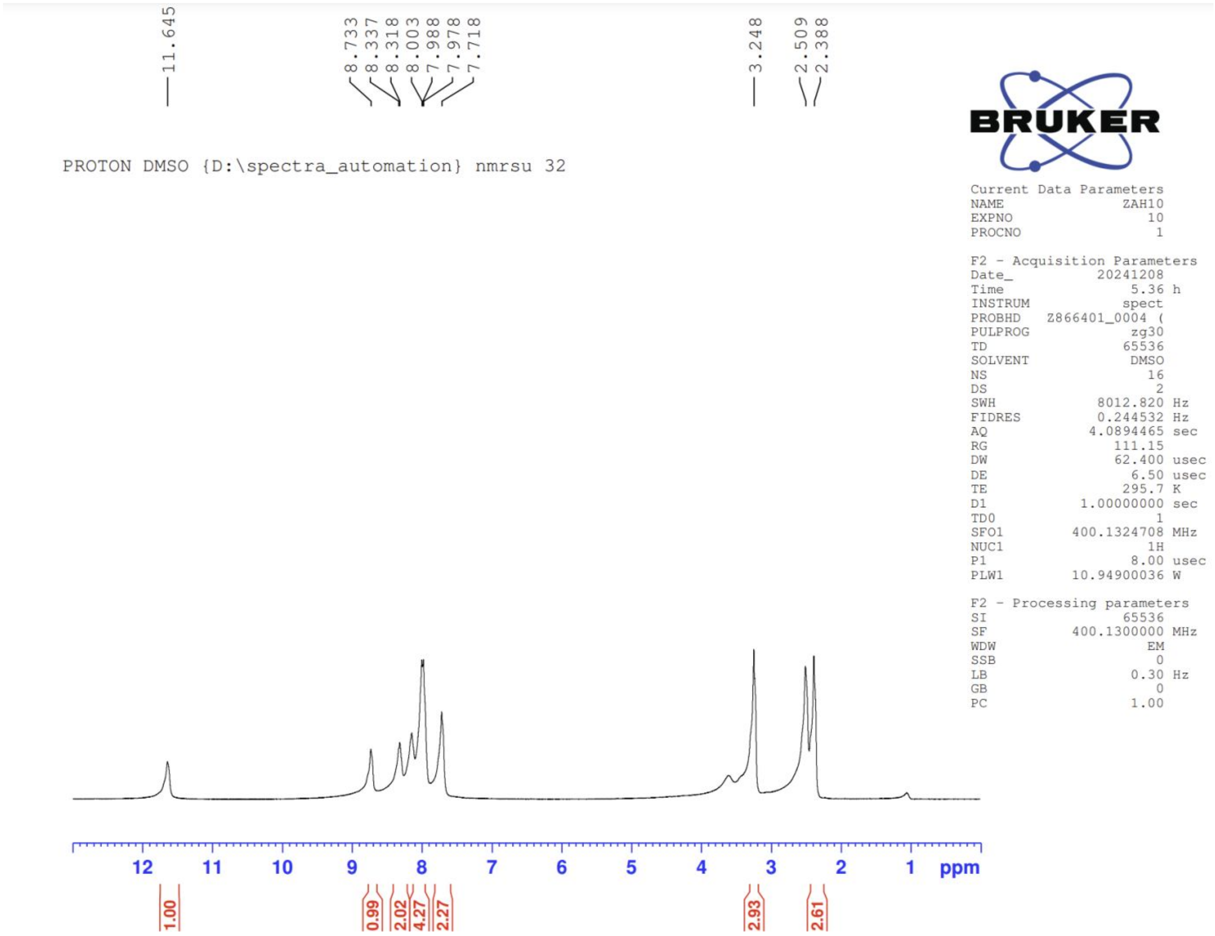

Figure S28. ^{1}H-NMR spectrum of compound **2j**

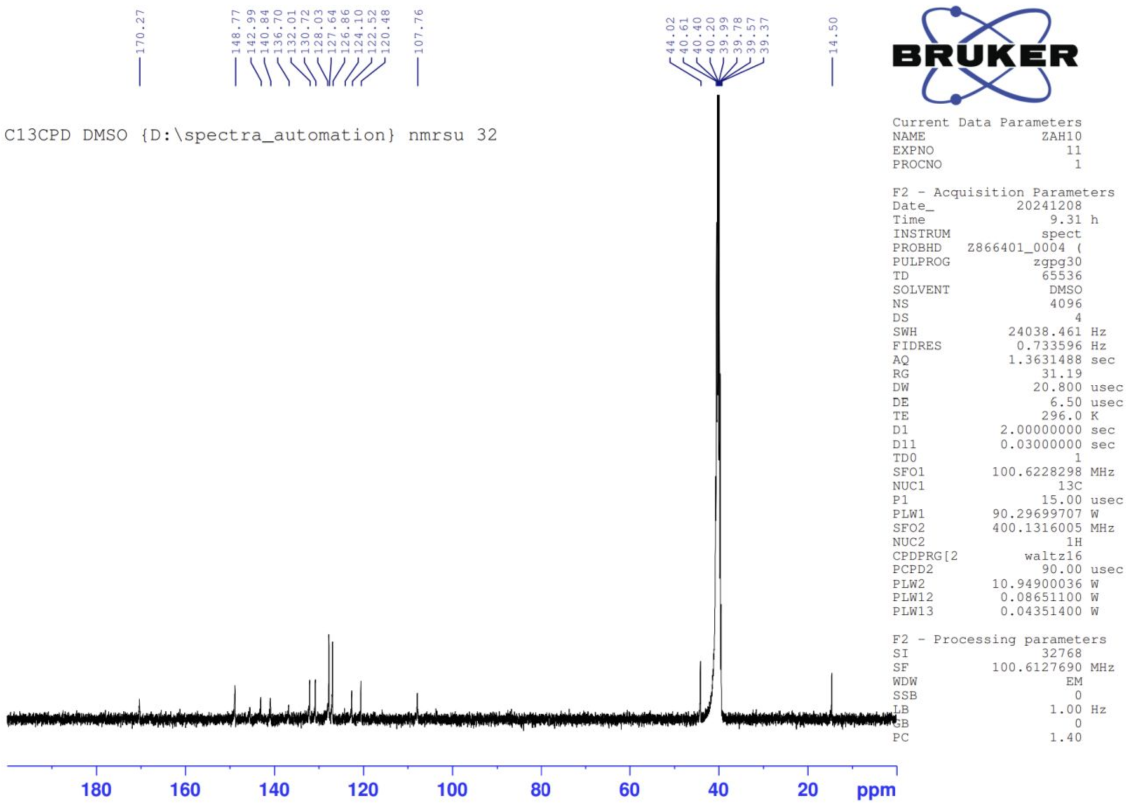

Figure S29. ^{13}C-NMR spectrum of compound **2j**

Formula Predictor Report - ZAH-10_103.lcd

Page 1 of 1

Data File: C:\LabSolutions\Data\Analiz\uac\ZAH-10_103.lcd

Elmt	Val.	Min	Max	Elmt	Val.	Min	Max	Elmt	Val.	Min	Max	Elmt	Val.	Min	Max	Use Adduct
H	1	5	40	O	2	0	4	S	2	2	2	Ru	2	0	0	H
C	4	5	30	F	1	0	0	Cl	1	0	0	Pd	2	0	0	
N	3	0	5	P	3	0	0	Br	1	0	0	I	3	0	0	

Error Margin (ppm): 5
HC Ratio: unlimited
Max Isotopes: 3
MSn Iso RI (%): 10.00

DBE Range: 12.0 - 25.0
Apply N Rule: yes
Isotope RI (%): 1.00
MSn Logic Mode: AND

Electron Ions: both
Use MSn Info: yes
Isotope Res: 9000
Max Results: 200

Event#: 1 MS(E+) Ret. Time : 3.587 Scan# : 539

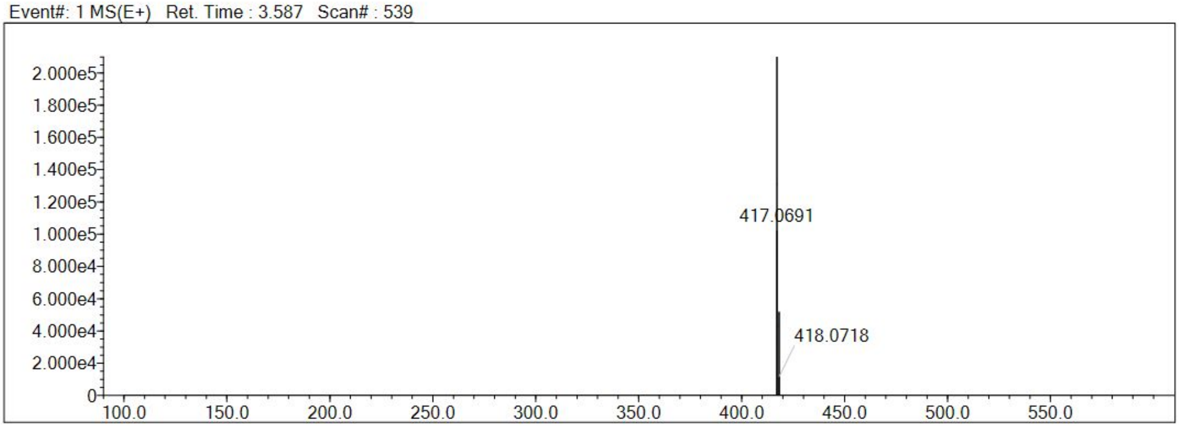

Measured region for 417.0691 m/z

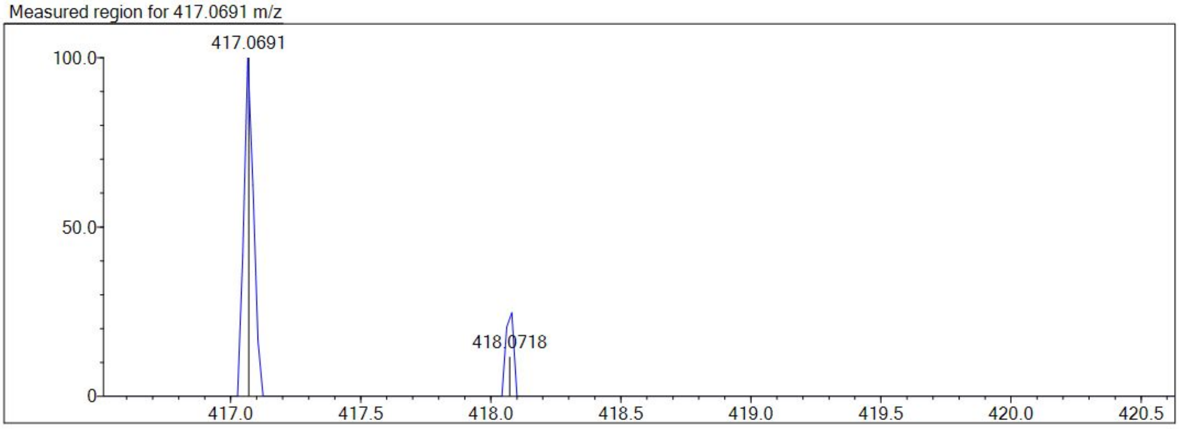

C18 H16 N4 O4 S2 [M+H]+ : Predicted region for 417.0686 m/z

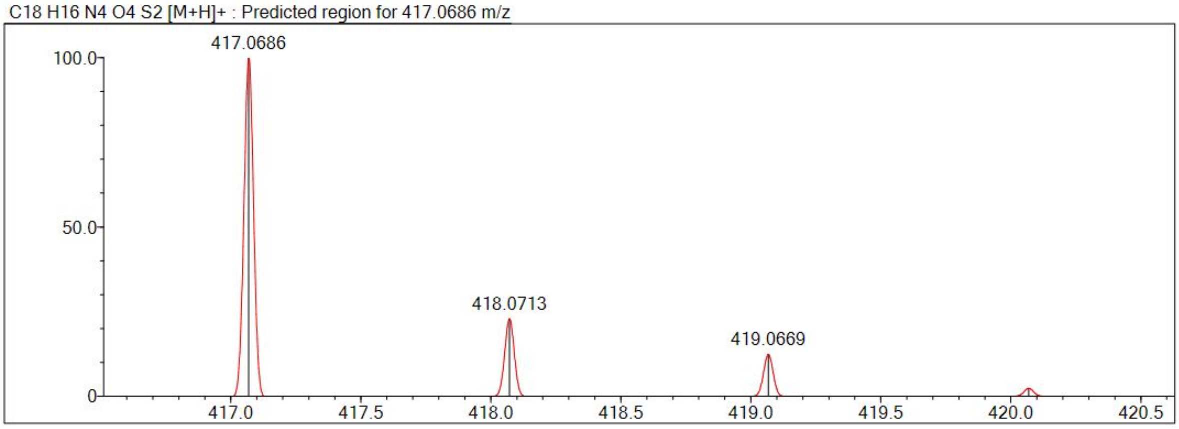

Rank	Score	Formula (M)	Ion	Meas. m/z	Pred. m/z	Df. (mDa)	Df. (ppm)	Iso	DBE
1	0.00	C18 H16 N4 O4 S2	[M+H]+	417.0691	417.0686	0.5	1.20	0.00	13.0

Figure S30. Mass spectrum of compound **2j**

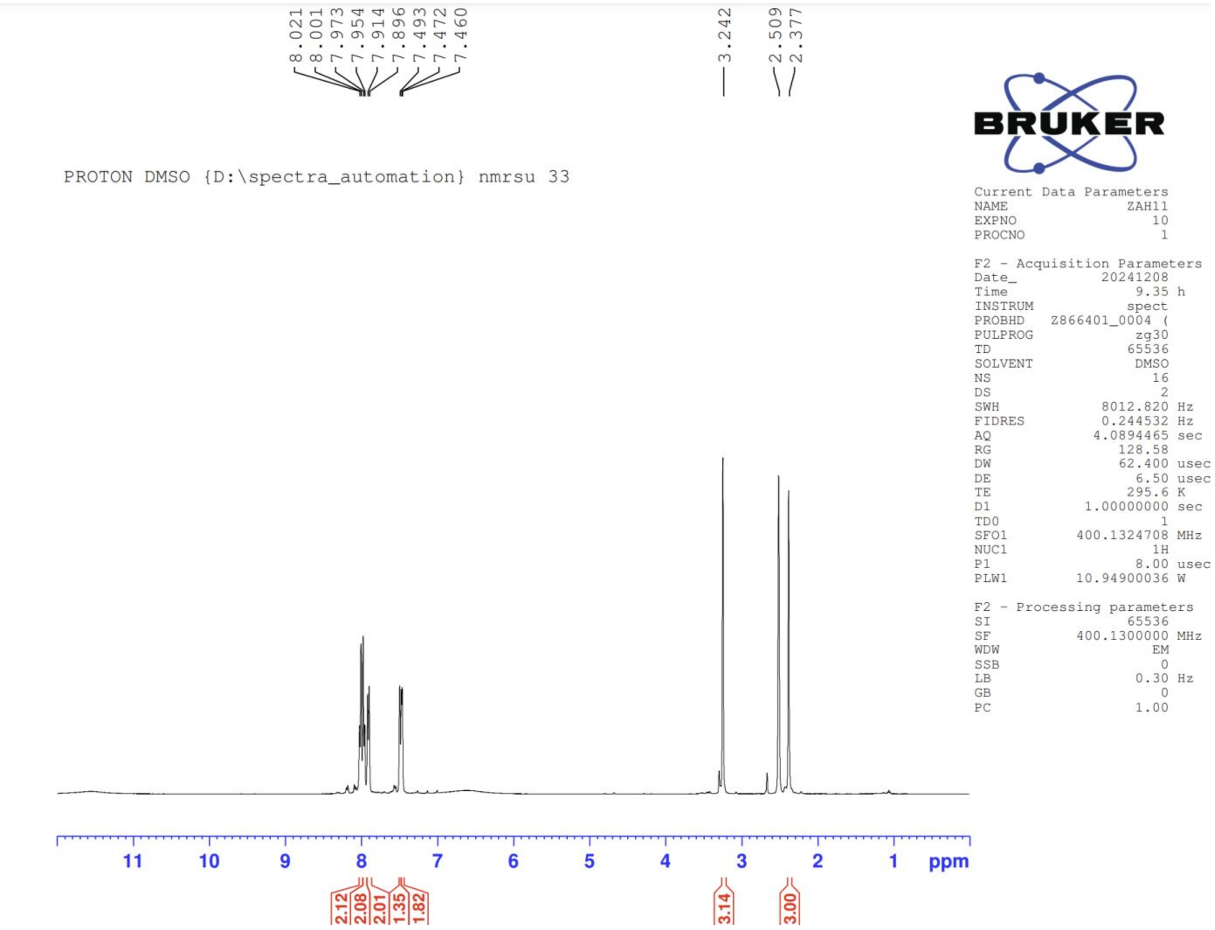

Figure S31. ^{1}H-NMR spectrum of compound **2k**

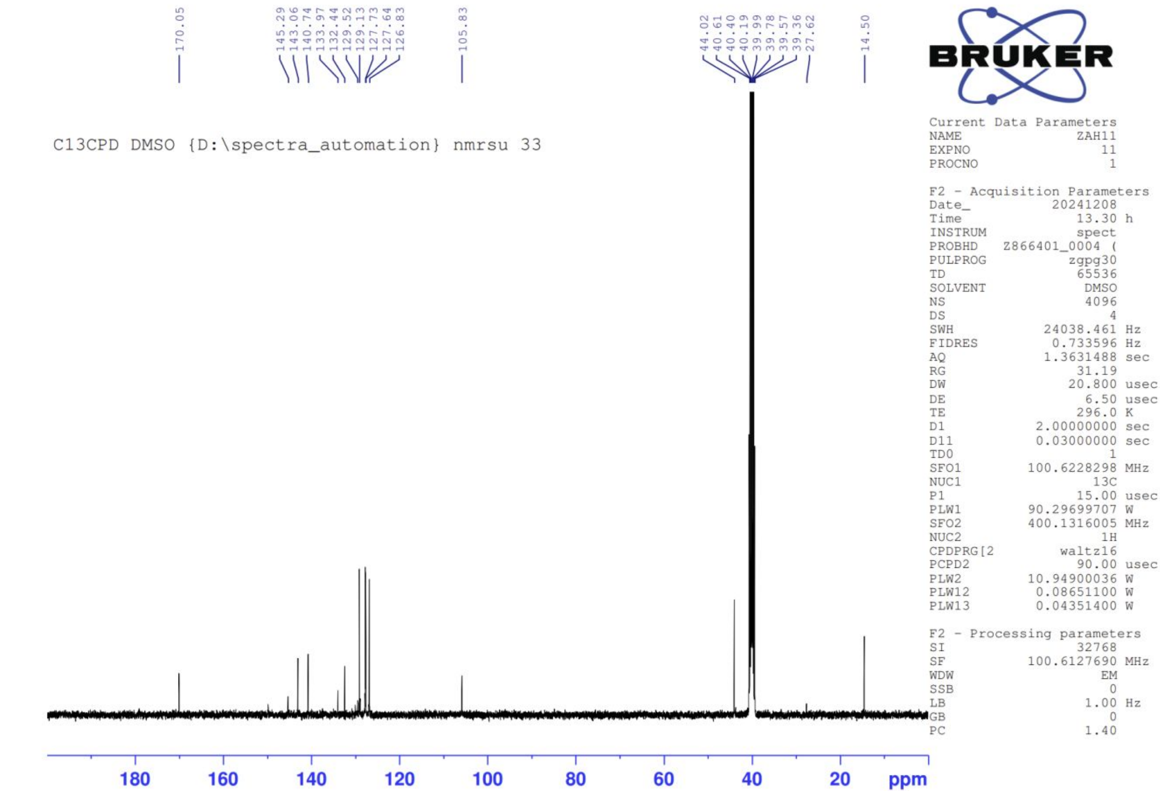

Figure S32. ^{13}C-NMR spectrum of compound **2k**

Formula Predictor Report - ZAH-11_104.lcd

Data File: C:\LabSolutions\Data\Analiz\uac\ZAH-11_104.lcd

Elmt	Val.	Min	Max	Elmt	Val.	Min	Max	Elmt	Val.	Min	Max	Elmt	Val.	Min	Max	Use Adduct
H	1	5	40	O	2	0	4	S	2	2	2	Ru	2	0	0	H
C	4	5	30	F	1	0	0	Cl	1	1	1	Pd	2	0	0	
N	3	0	5	P	3	0	0	Br	1	0	0	I	3	0	0	

Error Margin (ppm):	5	DBE Range:	12.0 - 25.0	Electron Ions:	both
HC Ratio:	unlimited	Apply N Rule:	yes	Use MSn Info:	yes
Max Isotopes:	3	Isotope RI (%):	1.00	Isotope Res:	9000
MSn Iso RI (%):	10.00	MSn Logic Mode:	AND	Max Results:	200

Event#: 1 MS(E+) Ret. Time : 4.427 -> 5.040 Scan# : 665 -> 757

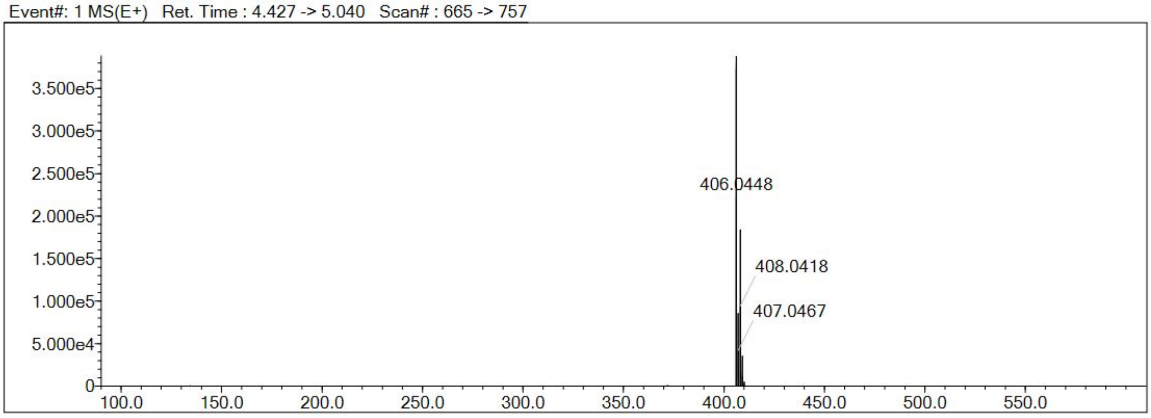

Measured region for 406.0448 m/z

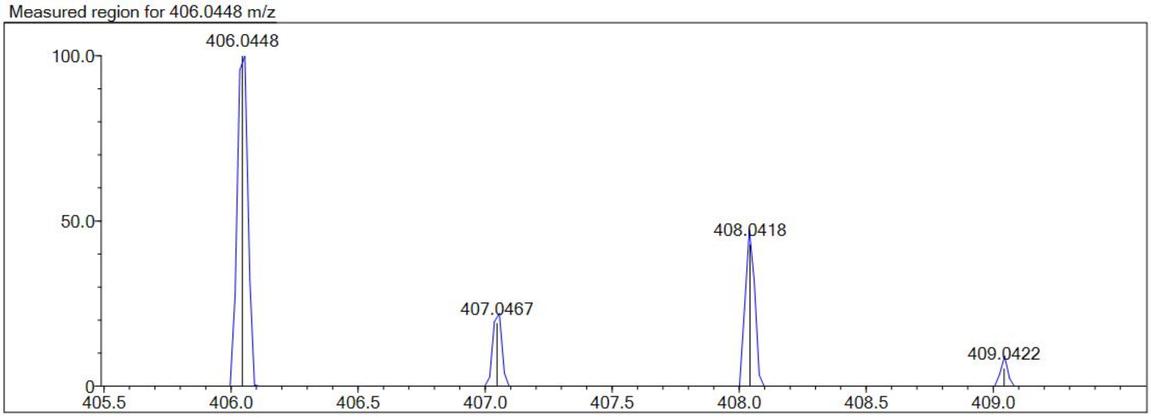

C18 H16 N3 O2 S2 Cl [M+H]+ : Predicted region for 406.0445 m/z

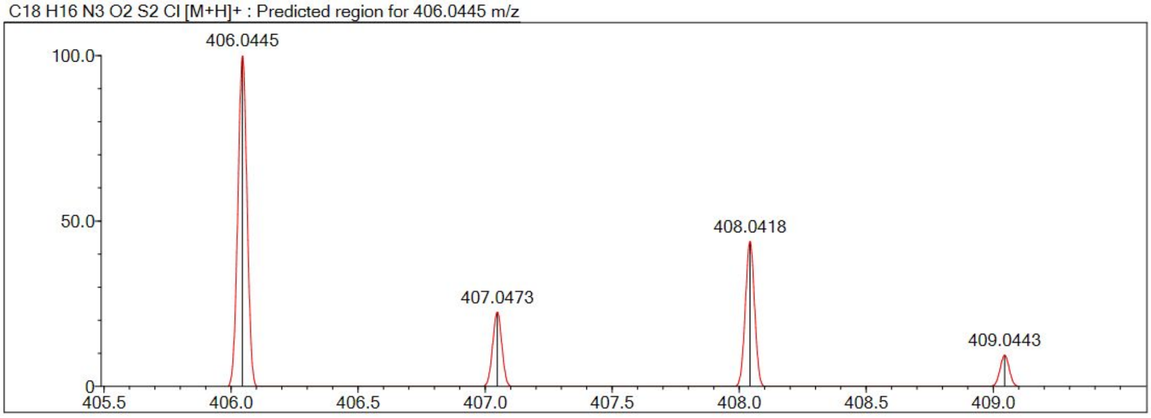

Rank	Score	Formula (M)	Ion	Meas. m/z	Pred. m/z	Df. (mDa)	Df. (ppm)	Iso	DBE
1	100.00	C18 H16 N3 O2 S2 Cl	[M+H]+	406.0448	406.0445	0.3	0.74	100.00	12.0

Figure S33. Mass spectrum of compound **2k**

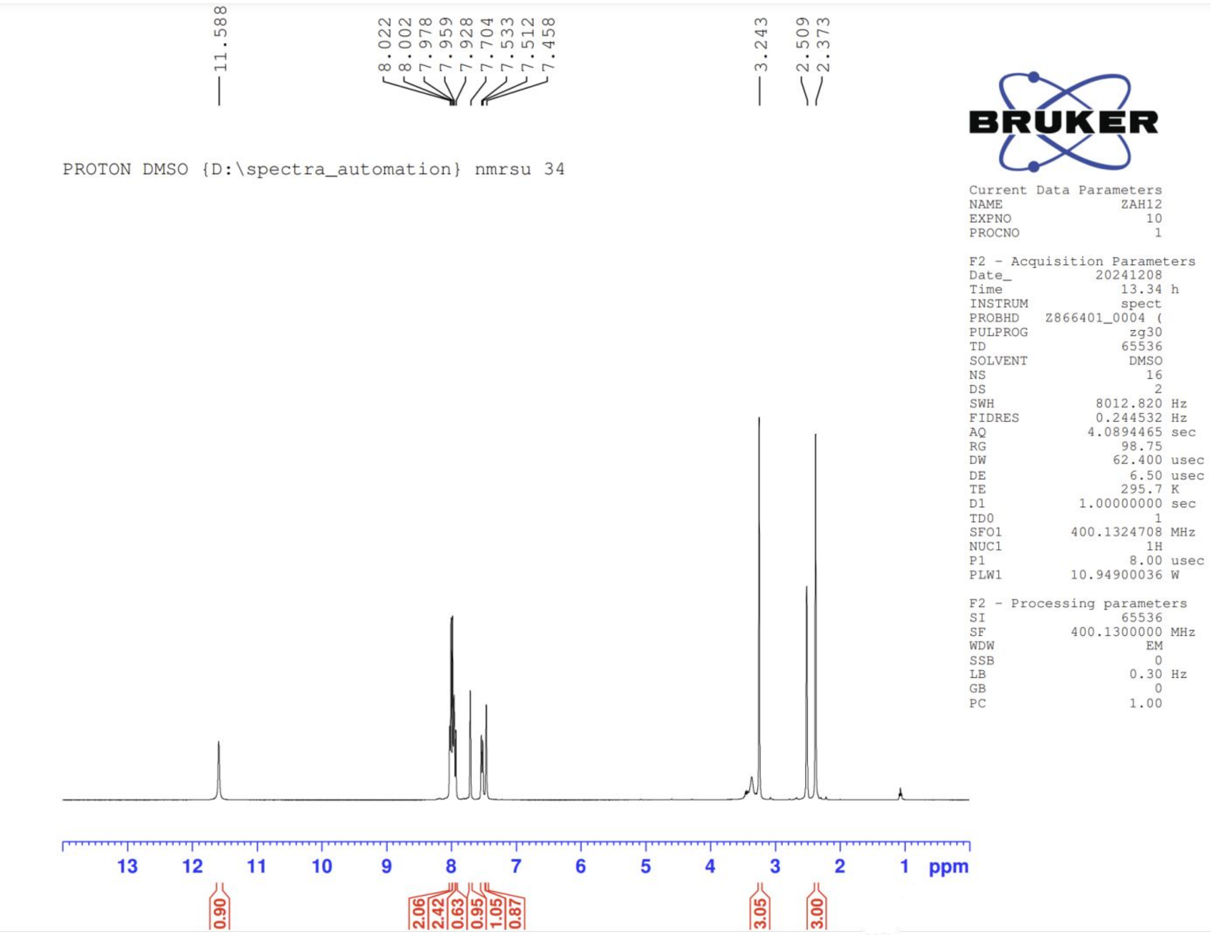

Figure S34. ^{1}H-NMR spectrum of compound **2l**

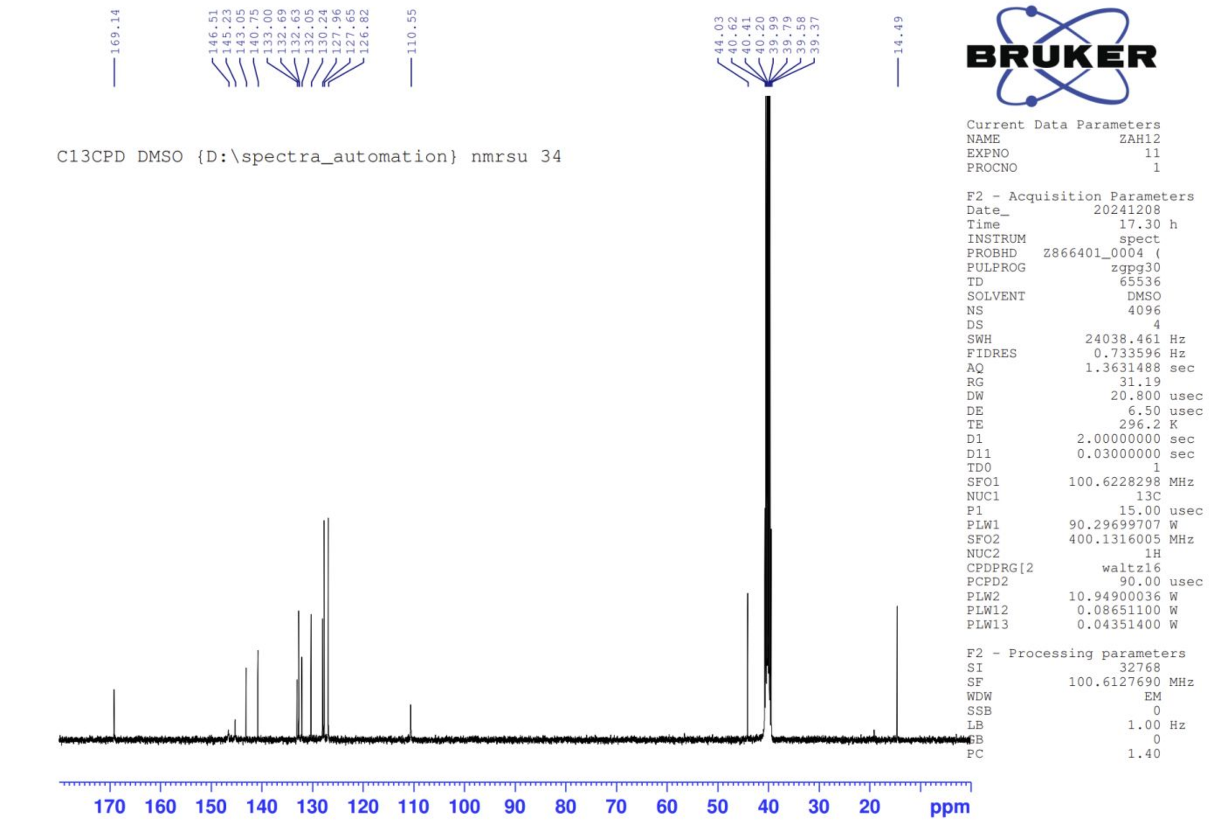

Figure S35. ^{13}C-NMR spectrum of compound **2l**

Data File: C:\LabSolutions\Data\Analiz\uac\ZAH-12_105.lcd

Elmt	Val.	Min	Max	Elmt	Val.	Min	Max	Elmt	Val.	Min	Max	Elmt	Val.	Min	Max	Use Adduct
H	1	5	40	O	2	0	4	S	2	2	2	Ru	2	0	0	H
C	4	5	30	F	1	0	0	Cl	1	2	2	Pd	2	0	0	
N	3	0	5	P	3	0	0	Br	1	0	0	I	3	0	0	

Error Margin (ppm): 5
HC Ratio: unlimited
Max Isotopes: 3
MSn Iso RI (%): 10.00

DBE Range: 12.0 - 25.0
Apply N Rule: yes
Isotope RI (%): 1.00
MSn Logic Mode: AND

Electron Ions: both
Use MSn Info: yes
Isotope Res: 9000
Max Results: 200

Event#: 1 MS(E+) Ret. Time : 7.080 -> 7.080 Scan# : 1063 -> 1063

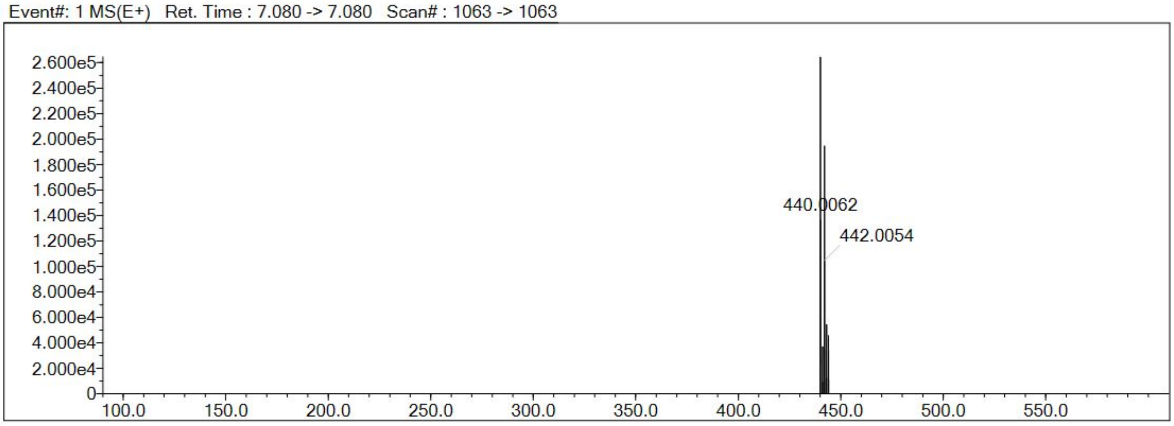

Measured region for 440.0062 m/z

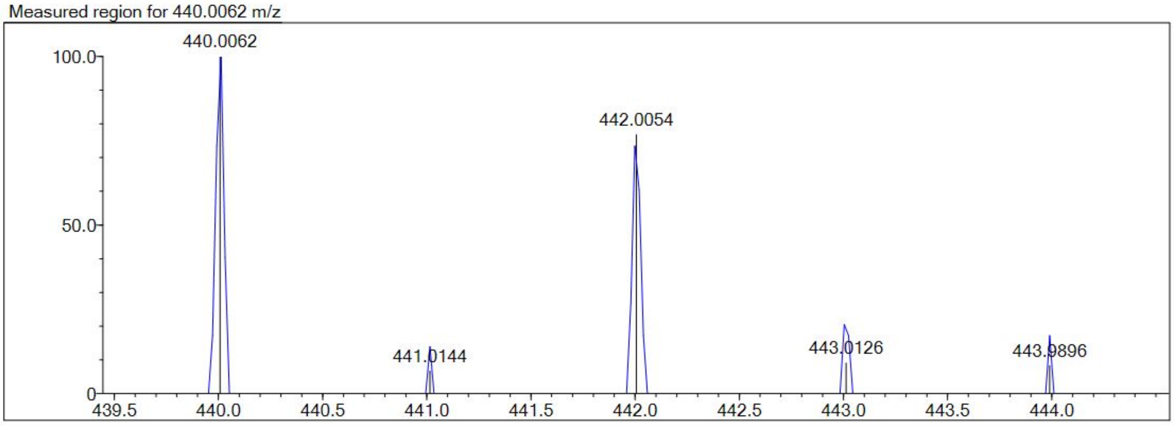

C18 H15 N3 O2 S2 Cl2 [M+H]+ : Predicted region for 440.0056 m/z

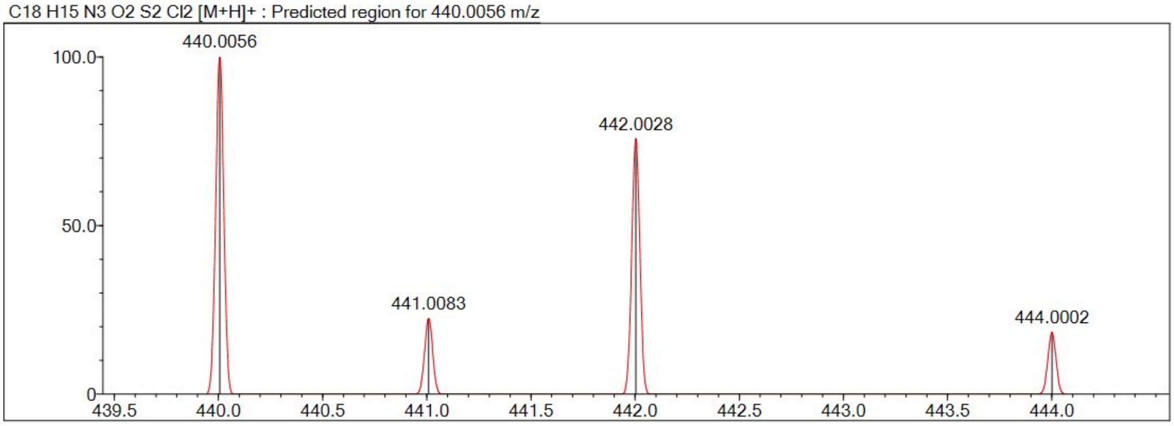

Rank	Score	Formula (M)	Ion	Meas. m/z	Pred. m/z	Df. (mDa)	Df. (ppm)	Iso	DBE
1	38.09	C18 H15 N3 O2 S2 Cl2	[M+H]+	440.0062	440.0056	0.6	1.36	38.43	12.0

Figure S36. Mass spectrum of compound **2l**

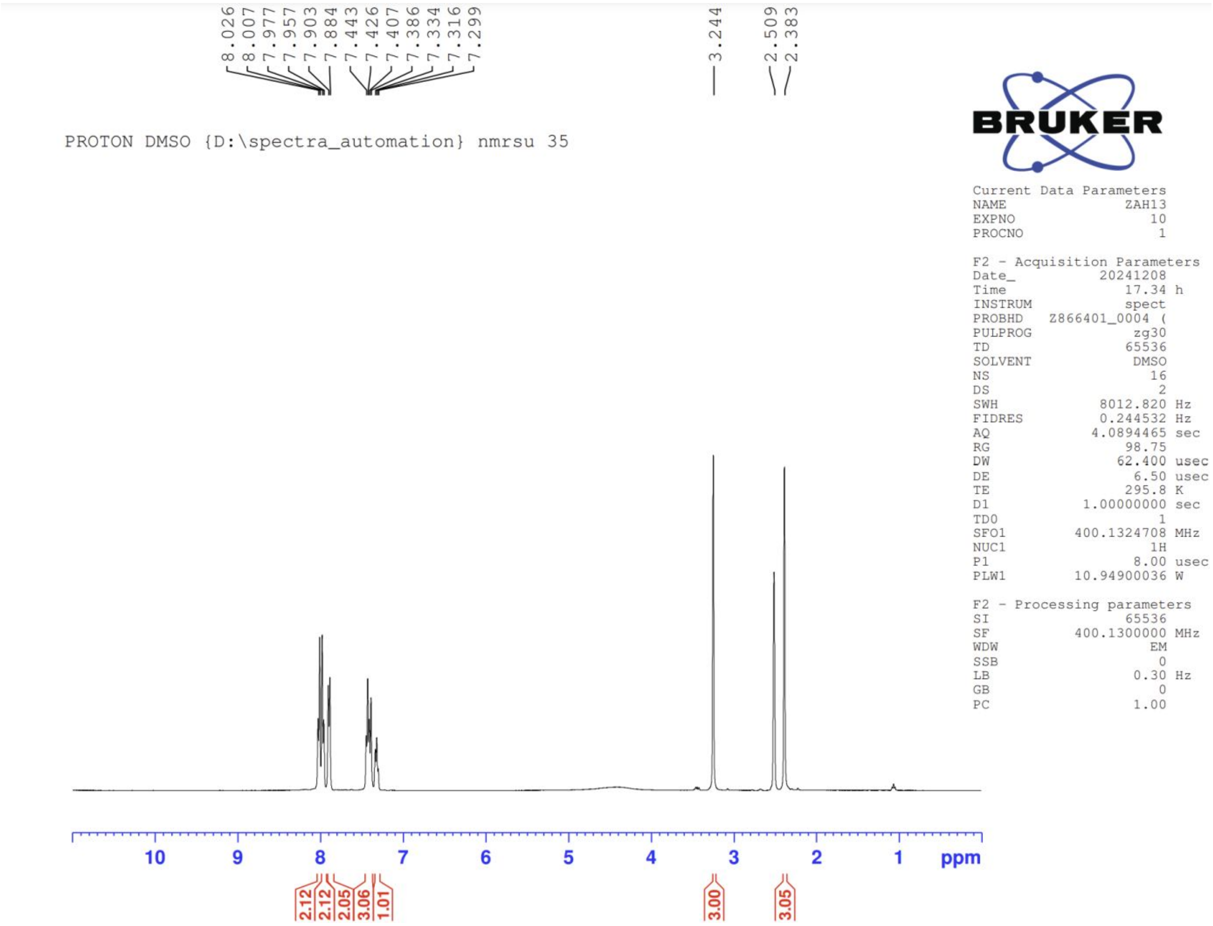

Figure S37. ^{1}H-NMR spectrum of compound **2m**

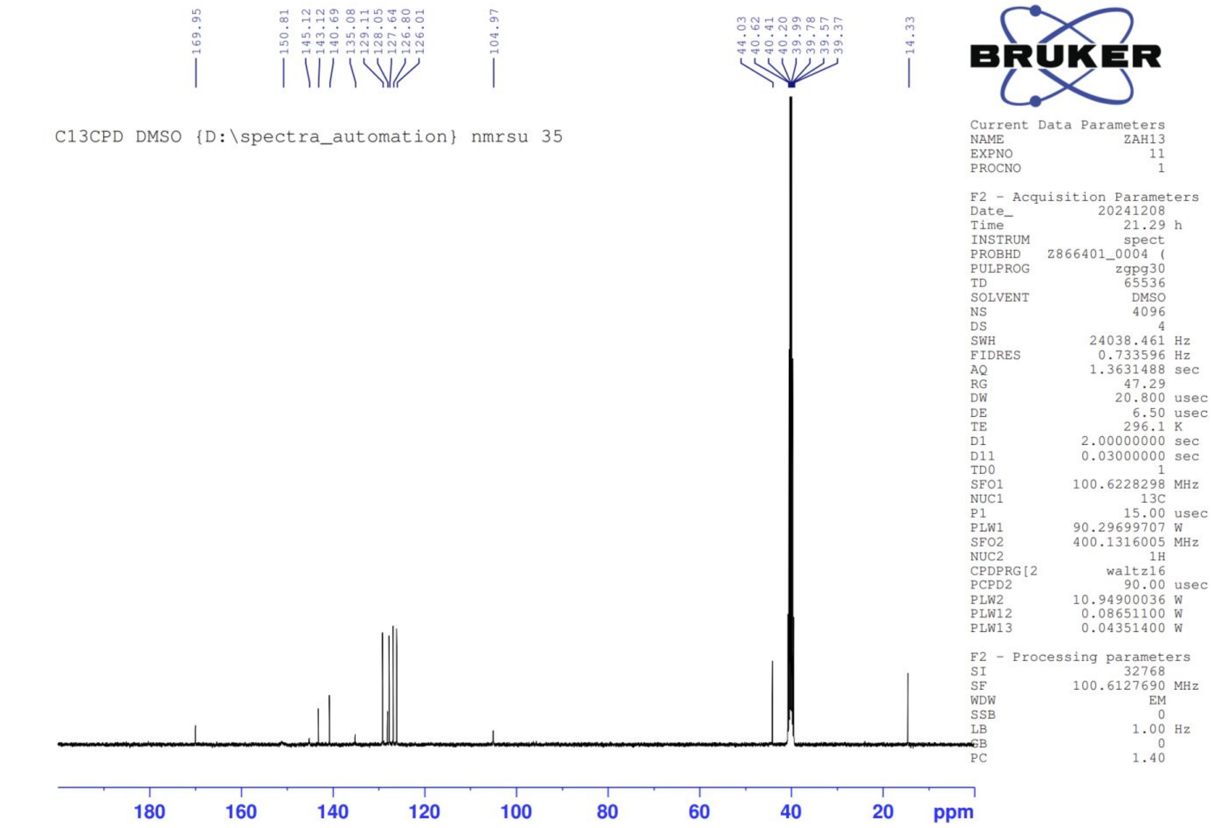

Figure S38. ^{13}C-NMR spectrum of compound **2m**

Data File: C:\LabSolutions\Data\Analiz\uac\ZAH-13_106.lcd

Elmt	Val.	Min	Max	Elmt	Val.	Min	Max	Elmt	Val.	Min	Max	Elmt	Val.	Min	Max	Use Adduct
H	1	5	40	O	2	0	4	S	2	2	2	Ru	2	0	0	H
C	4	5	30	F	1	0	0	Cl	1	0	0	Pd	2	0	0	
N	3	0	5	P	3	0	0	Br	1	0	0	I	3	0	0	

Error Margin (ppm): 5
HC Ratio: unlimited
Max Isotopes: 3
MSn Iso RI (%): 10.00

DBE Range: 12.0 - 25.0
Apply N Rule: yes
Isotope RI (%): 1.00
MSn Logic Mode: AND

Electron Ions: both
Use MSn Info: yes
Isotope Res: 9000
Max Results: 200

Event#: 1 MS(E+) Ret. Time : 3.187 -> 3.880 Scan# : 479 -> 583

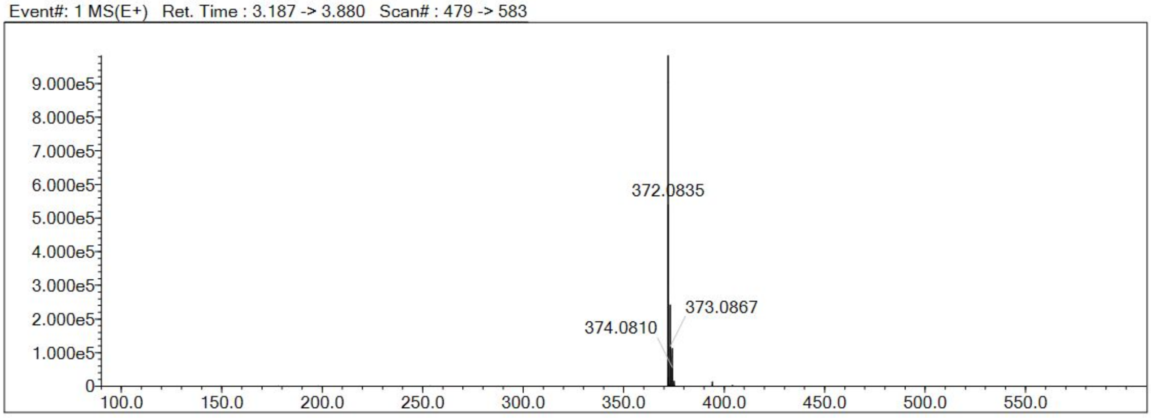

Measured region for 372.0835 m/z

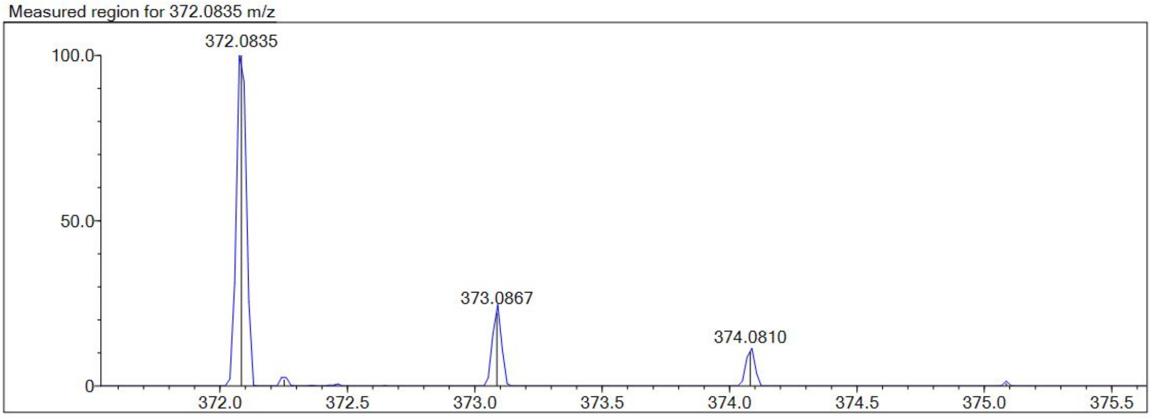

C18 H17 N3 O2 S2 [M+H]+ : Predicted region for 372.0835 m/z

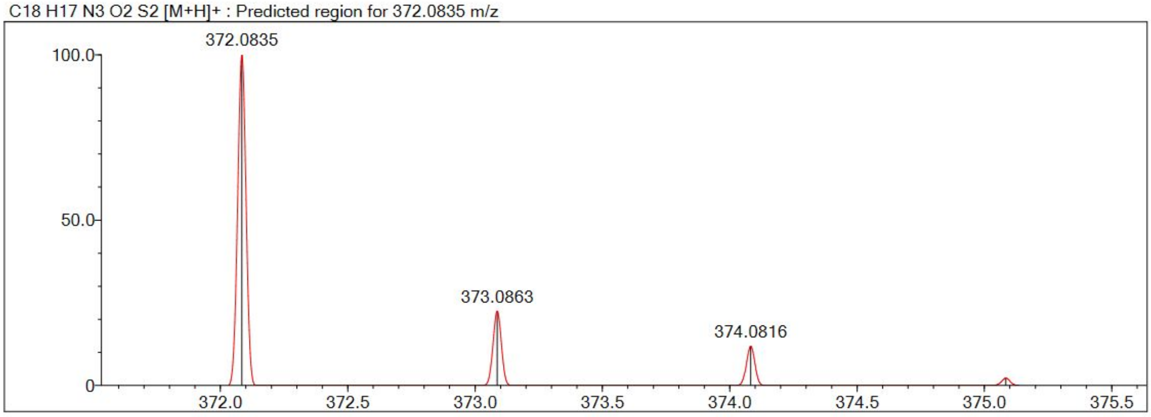

Rank	Score	Formula (M)	Ion	Meas. m/z	Pred. m/z	Df. (mDa)	Df. (ppm)	Iso	DBE
1	99.56	C18 H17 N3 O2 S2	[M+H]+	372.0835	372.0835	0.0	0.00	99.56	12.0

Figure S39. Mass spectrum of compound **2m**
